# Safety, Immunogenicity and Duration of Protection of the RTS,S/AS02_D_ Malaria Vaccine: One Year Follow-Up of a Randomized Controlled Phase I/IIb Trial

**DOI:** 10.1371/journal.pone.0013838

**Published:** 2010-11-04

**Authors:** Pedro Aide, John J. Aponte, Montse Renom, Tacilta Nhampossa, Jahit Sacarlal, Inacio Mandomando, Quique Bassat, Maria Nélia Manaca, Amanda Leach, Marc Lievens, Johan Vekemans, Marie-Claude Dubois, Christian Loucq, W. Ripley Ballou, Joe Cohen, Pedro L. Alonso

**Affiliations:** 1 Centro de Investigação em Saúde da Manhiça (CISM), Maputo, Mozambique; 2 Barcelona Centre for International Health Research, Hospital Clinic, University of Barcelona, Barcelona, Spain; 3 Instituto Nacional de Saúde, Ministério de Saúde, Maputo, Mozambique; 4 Faculdade de Medicina, Universidade Eduardo Mondlane, Maputo, Mozambique; 5 Glaxo-SmithKline Biologicals, Rixensart, Belgium; 6 PATH Malaria Vaccine Initiative, Bethesda, Maryland, United States of America; Swiss Tropical Institute, Switzerland

## Abstract

**Background:**

The RTS,S/AS02_D_ vaccine has been shown to have a promising safety profile, to be immunogenic and to confer protection against malaria in children and infants.

**Methods and Findings:**

We did a randomized, controlled, phase I/IIb trial of RTS,S/AS02_D_ given at 10, 14 and 18 weeks of age staggered with routine immunization vaccines in 214 Mozambican infants. The study was double-blind until the young child completed 6 months of follow-up over which period vaccine efficacy against new *Plasmodium falciparum* infections was estimated at 65.9% (95% CI 42.6–79.8, p<0.0001). We now report safety, immunogenicity and estimated efficacy against clinical malaria up to 14 months after study start. Vaccine efficacy was assessed using Cox regression models. The frequency of serious adverse events was 32.7% in the RTS,S/AS02_D_ and 31.8% in the control group. The geometric mean titers of anti-circumsporozoite antibodies declined from 199.9 to 7.3 EU/mL from one to 12 months post dose three of RTS,S/AS02_D_, remaining 15-fold higher than in the control group. Vaccine efficacy against clinical malaria was 33% (95% CI: −4.3–56.9, p = 0.076) over 14 months of follow-up. The hazard rate of disease per 2-fold increase in anti-CS titters was reduced by 84% (95% CI 35.1–88.2, p = 0.003).

**Conclusion:**

The RTS,S/AS02_D_ malaria vaccine administered to young infants has a good safety profile and remains efficacious over 14 months. A strong association between anti-CS antibodies and risk of clinical malaria has been described for the first time. The results also suggest a decrease of both anti-CS antibodies and vaccine efficacy over time.

**Trial Registration:**

ClinicalTrials.gov NCT00197028

## Introduction

*Plasmodium falciparum* malaria is one of the most serious public health problems worldwide[1]. The need for improved prevention tools cannot be overemphasized. A safe and effective malaria vaccine to be used in malaria-endemic areas, particularly during early stages of life, could greatly contribute to reducing the enormous burden of malaria, and perhaps contribute to future eradication efforts.

The last decade has witnessed important progresses in the development of a first generation malaria vaccine. GlaxoSmithKline's (GSK) RTS,S, formulated with the Adjuvant System AS02 or AS01, is currently the world's most clinically-advanced malaria vaccine candidate. This vaccine has been shown to be safe and efficacious against malaria infection and disease in adult naïve and semi-immune volunteers [2], [3]. In 2004, we reported the first proof-of-concept study in African children aged 1 to 4 years showing that the vaccine was safe, immunogenic and reduced the risk of *P. falciparum* infection, uncomplicated malaria and severe disease, and that protection lasted for at least 45 months [4], [5], [6].

Recognizing that malaria control strategies must prioritize protection in infants [7], [8], [9] led us to a I/IIb proof-of-concept trial to assess the safety, immunogenicity and efficacy of RTS,S/AS02_D_ in children less than 12 months of age. Vaccine efficacy (VE) against malaria infection was 65.9% (95% CI 42.6–79.8, p<0.0001) at the end of 6 months of follow-up [10].

A subsequent trial of the RTS,S/AS02_D_ in Tanzanian infants has recently shown very similar results [vaccine efficacy of 65.2% (95% CI 20.7–84.7, p = 0.01)] [11]. Furthermore, another trial in children 5–17 months old with RTS,S/AS01_E_ in Tanzania and Kenya yielded a 53% (95% CI 28–69, p<0.001) reduction of clinical malaria episodes over an 8 month follow up period [12].

This paper reports the safety, reactogenicity, immunogenicity and efficacy of the complete 14 months follow-up period of the Mozambican phase I/IIb proof-of-concept trial in infants, with particular emphasis on safety and reactogenicity, given that it was the first time that RTS,S formulated with AS02 was administered to infants.

## Methods

The protocol for this trial and supporting CONSORT checklist are available as supporting information; see Checklist S1 and Protocol S1.

### Study site

The study was carried out by the Centro de Investigação em Saúde de Manhiça (CISM) in the rural areas of Taninga and Ilha Josina Machel, 50 Km north of Manhiça village, Mozambique, from June 2005 to December 2007. Detailed description of the area can be found elsewhere [10], [13].

### Study Design

This study was a phase I/IIb, randomized controlled trial to assess the safety, immunogenicity and efficacy of the RTS,S/AS02_D_ vaccine administered to infants at 10, 14 and 18 weeks of age, staggered with EPI vaccines (DTPw/Hib [TETRActHib™ Aventis Pasteur]) at 8, 12 and 16 weeks of age. The study was double-blind until the youngest child completed 6 months of follow-up. After the unblinding, the study was considered single blinded although both participants and field investigators remained blinded. Only a senior statistician had access to the treatment codes allocated to the subjects, and he was not involved in the children follow-up. Data provided to the field investigators did not include information of the allocated treatment per subject during the entire duration of the trial.

A total of 214 children were enrolled and randomized to receive either RTS,S/AS02_D_ or the control hepatitis B vaccine, *Engerix-B*™. Details of the malaria and control vaccines as well as the trial profile for the double-blind phase have been presented elsewhere [10]. Briefly, all women who considered enrolling their infant in the study were screened for hepatitis B surface antigen (HBsAg) and human immunodeficiency virus (HIV) in their third trimester of pregnancy. Written informed consent was obtained before any blood was taken for testing.

Infants were screened between 6 and 12 weeks of age and a second written informed consent was obtained from parents/guardians of all participants. Infants were enrolled if they were born after a normal gestational period and in the absence of obvious medical abnormalities. Children born to Hepatitis B and HIV positive mothers were not included in the trial. Children were excluded as well from participation if BCG vaccine had not been given at least one week before the first study vaccination or if any other vaccinations, other than the first dose of oral polio vaccine (OPV) given at birth with BCG, had been given prior to enrolment. Identification cards were provided soon after recruitment. Study activities were completed on December 27^th^, 2007, when the last recruited child completed 14 months of follow-up.

The protocol (NCT00197028) was approved by the Mozambican National Bioethics Committee, the Hospital Clínic of Barcelona Ethics Review Committee and the PATH Human Subjects Protection Committee and implemented according to the International Conference of Harmonization and Good Clinical Practices guidelines. GSK monitored the study. A Local Safety Monitor and a Data and Safety Monitoring Board oversaw the design, conduct and results of the trial.

The sample size for the original study was based on an evaluation of vaccine safety [10]. A trial with 100 subjects in each group had 80% power to detect a 2.6-fold increase in SAE rates if the rate in controls was at least 10%. The trial also had 90% power to detect an efficacy against malaria infection of 45% or more assuming an attack rate of at least 75% in the control group over the surveillance period. Efficacy against clinical malaria was an exploratory endpoint.

### Evaluation of safety

Safety endpoints included the occurrence of solicited and unsolicited symptoms within 7 and 30 days after each vaccination respectively and the occurrence of serious adverse events (SAEs) during the entire 14 month follow-up period. All SAEs were reported within 24 hours after detection.

Vaccine safety was evaluated using active and passive follow-up. All study participants were observed for at least one hour after each vaccine dose by a physician equipped with an emergency kit. Children were visited daily in their homes for 6 days after vaccination where any adverse events (AEs), local or general, were registered on diary cards. Study physicians evaluated all suspected grade 3 AEs and guided clinical management.

Passive follow-up was done through a health facility based morbidity surveillance system [5], [14]. All AEs irrespective of their severity or relationship to vaccination were recorded during the 30 day period after each dose. SAEs were similarly detected and reported throughout the study. Detailed definitions for solicited and unsolicited AEs and SAEs as well as the classification of the intensity can be found elsewhere [15].

Participants with SAEs were followed-up until events resolved. Deaths occurring at home were investigated by a review of available medical records and by verbal autopsy, as described elsewhere [16].

Safety monitoring of hematological parameters [hemoglobin, hematocrit, whole blood cell (WBC) and platelets] and biochemical parameters [alanine amino transferase (ALT), total bilirubin and creatinine] were measured one week after dose 1 and 1, 3½, and 12 months after dose 3. Normality values considered were: hemoglobin ≥80 g/L, hematocrit ≥25%, WBC 5-17×10^9^/L, platelets ≥100×10^9^/L, ALT ≤60 µmol/L, creatinine ≤45 µmol/L and bilirubin ≤34 µmol/L.

Biochemical, hematological and packed cell volume (PCV) tests were determined as described elsewhere [10].

### Evaluation of immunogenicity

Antibody titres were measured against hepatitis B surface antigen (anti-HBs) and *P. falciparum* circumsporozoite protein (anti-CS) at screening and 1, 3½ and 12 months post dose 3.

Anti-CS antibodies were measured by a standardized ELISA, using plates absorbed with recombinant R32LR with an assay cut-off of 0.5 EU/mL. Anti-HBs antibodies were quantified using the EIA kit from Abbott Laboratories and a GSK validated sandwich ELISA described elsewhere [17]. The cut-off for the anti-HBs ELISA was set at 10 mIU/mL.

### Evaluation of vaccine efficacy

Cases of clinical malaria and malaria infection by *P. falciparum* were ascertained by a combination of passive case detection (PCD) and active detection of infection (ADI) as described elsewhere [10]. Briefly, two weeks prior to dose 3, all children received a combination of amodiaquine and sulfadoxine-pyrimethamine to clear any parasitemia. Two weeks after dose 3, children with negative slides started ADI (performed bi-weekly for 12 weeks). At each ADI visit, axillary temperature was recorded and parasitemia determined. Children with positive results received antimalarial treatment regardless of the presence or absence of symptoms and were withdrawn from further ADI evaluation. PCD was performed at Manhica District Hospital and Ilha Josina and Taninga Health posts as described elsewhere [5], [14].

The primary case definition of clinical malaria was the presence of fever (axillary temperature ≥37.5°C) with a *P. falciparum* asexual parasitemia >500/µL. This definition has a sensitivity and specificity >90% in this age group [18]. The secondary case definition was fever or history of fever in the previous 24 hours plus any asexual *P. falciparum* parasitemia.

Exploratory efficacy endpoints were first or only clinical episode of *P. falciparum* malaria as well as multiple episodes of clinical malaria detected by PCD during 14 months after dose 1. An additional endpoint was first or only clinical episode of *P. falciparum* malaria detected by a combination of ADI and PCD.

### Statistical methods

Analyses were done for intention to treat (ITT) and according to protocol (ATP) cohorts, following a predefined analytical plan. The ITT cohort included all children who received at least one dose of the study vaccine. All safety analyses were based on ITT. The ATP cohort included participants that met all eligibility criteria, completed the vaccination course and contributed to follow-up time during the evaluation period. For exploratory analyses, the ATP cohort was split into two follow-up periods: follow-up over study months 3–9 (ATP_3–9_) and study months 3–14 (ATP_3–14_). VE explored both first or only episode and multiple episodes of clinical malaria detected during the two study periods.

Analysis of immunogenicity was based on the ATP cohort, excluding children that received any blood product, immunosuppressant or immune-modifying therapy. Measurements of anti-CS and anti-HBs antibodies were summarized by Geometric Mean Titres (GMTs) with 95% confidence intervals (95% CI). Titres below the cut-off were assigned an arbitrary value of half the cut-off of the assay for the purpose of GMT calculation.

Person years at risk (PYAR) accounted for absences from the study area and use of antimalarial drugs as previously described [5].

VE was defined as 1 minus the hazard ratio multiplied by 100 [(1 – HR)*100] and was adjusted for distance to health facility [5] and community of residence. The adjusted VE was assessed using Cox regression models (for the first or only episode) and Poisson regression (for multiple episodes).

A test based on the Schoenfeld residuals was performed to assess whether the hazard was constant over the surveillance period, and alternative approaches were applied if the assumption of proportional hazards was not supported.

The risk of clinical malaria as a function of immune response was evaluated by comparing post-vaccination anti-CS titters for RTS,S/AS02_D_ recipients who either did or did not experience at least one episode of clinical malaria meeting the primary case definition over ATP_3–14_ follow-up, using the Wilcoxon Rank Sum test. The hazard rate per 2-fold increase in post-vaccination anti-CS response was calculated for both ATP_3–9_ and ATP_3–14_ follow-up, along with their 95% confidence intervals.

Analyses were performed using SAS version 9.1 (Cary, NC, USA) and STATA version 10 (College Station, TX, USA).

## Results

**Figure 1:**
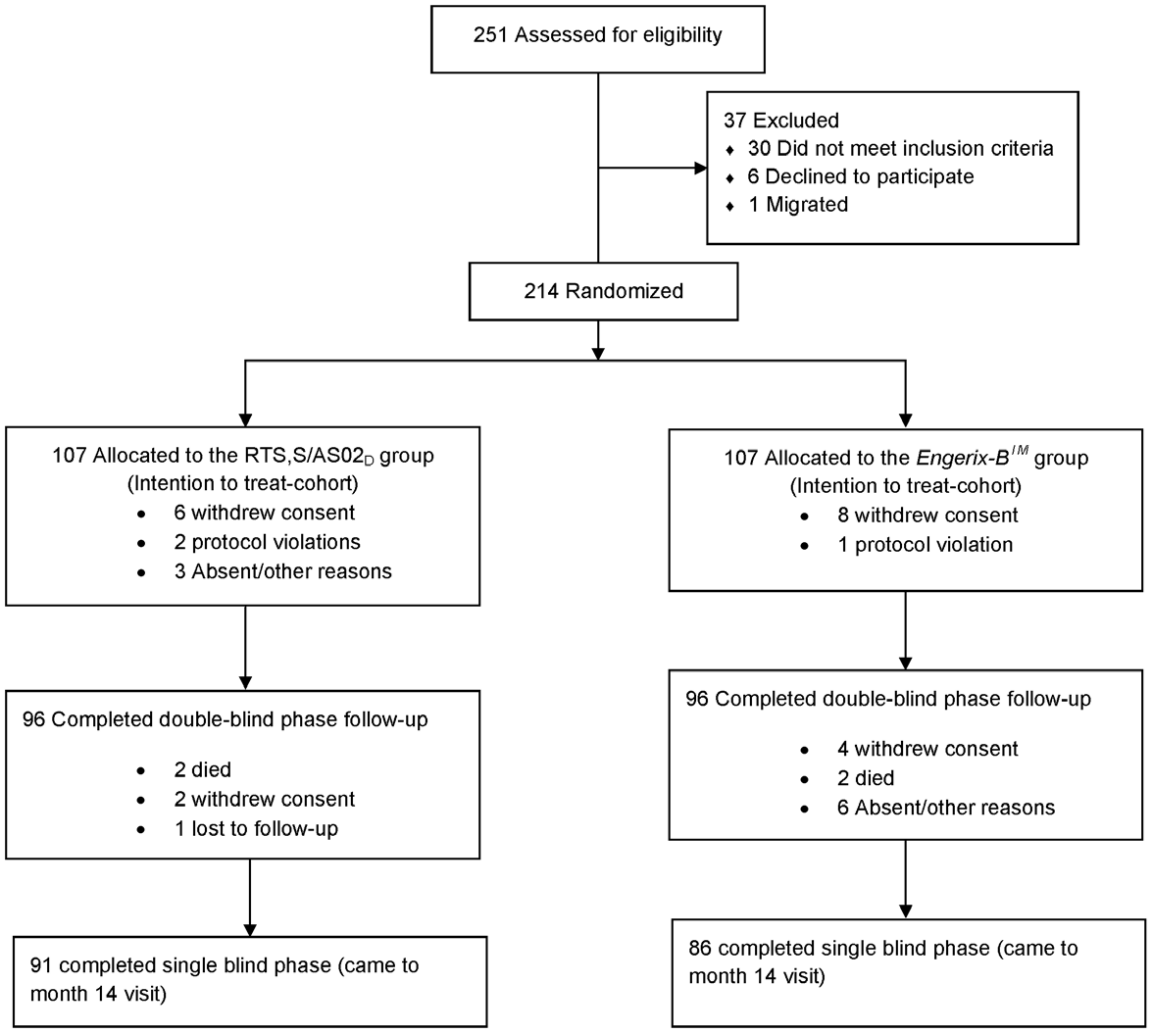
Trial Profile.

Of the 251 infants aged 6 to 12 weeks screened for eligibility, 214 were recruited and randomized to the RTS,S/AS02_D_ group (107) or the control group (107). A total of 177 children completed the 14 months follow-up period: 91 in the RTS,S/AS02_D_ group and 86 in the *Engerix-B*™ group (Fig. 1). Results of the initial 3.5 months of follow-up were reported elsewhere [10].

### Vaccine safety

Safety data was available for 214 children. 107 received 301 doses of RTS,S/AS02_D_ and 309 doses of *TETRActHib*™ and 107 received 303 doses of *Engerix-B*™ and 311 doses of *TETRActHib*™. Compliance for completion of symptoms questionnaires was 100%.

#### Solicited AEs after RTS,S/AS02_D_ or Engerix-B™ vaccinations

Three recipients of the *Engerix-B*™ vaccine reported grade 3 solicited general symptoms, all of them considered to be related to the vaccine but resolving within the 7 day follow-up period (Table 1). None of the RTS,S/AS02_D_ group participants reported grade 3 solicited general events. None of the solicited local symptoms reported in either group were of grade 3 intensity.

**Table 1:** Incidence of solicited general symptoms by dose within the 7-day follow-up after RTS,S/AS02_D_ or *Engerix-B™*.

	After dose 1				After dose 2				After dose 3			
	RTS,S/AS02_D_		*Engerix -B*		RTS,S/AS02_D_		*Engerix -B*		RTS,S/AS02_D_		*Engerix-B*	
	(N = 105)		(N = 106)		(N = 99)		(N = 100)		(N = 97)		(N = 97)	
	n	%	n	%	N	%	n	%	n	%	n	%
**General symptoms**												
Drowsiness												
Any	27	25.7	37	34.9	32	32.3	26	26.0	28	28.9	32	33.0
Related	10	9.5	11	10.4	8	8.1	3	3.0	3	3.1	5	5.2
Fever												
Any	11	10.5	5	4.7	10	10.1	10	10.0	8	8.2	9	9.3
Related	11	10.5	5	4.7	10	10.1	9	9.0	8	8.2	9	9.3
Grade 3	0	-	0	-	0	-	1	1.0	0	-	1	1.0
Grade 3 related	0	-	0	-	0	-	1	1.0	0	-	1	1.0
Irritability												
Any	43	41.0	39	36.8	49	49.5	39	39.0	42	42.3	46	47.4
Related	27	25.7	13	12.3	25	25.3	16	16.0	17	17.5	20	20.6
Grade 3	0	-	0	-	0	-	0	-	0	-	1	1.0
Grade 3 related	0	-	0	-	0	-	0	-	0	-	1	1.0
Loss of appetite												
Any	19	18.1	27	25.5	25	25.3	24	24.0	28	28.9	29	29.9
Related	2	1.9	1	0.9	1	1.0	1	1.0	3	3.1	3	3.1
**Local symptoms**												
Pain												
Any	103	98.1	95	89.6	92	92.9	82	82.0	80	82.5	81	83.5
Grade 3	0	-	0	-	0	-	0	-	0	-	0	-
Swelling												
Any	10	9.5	12	11.3	11	11.1	8	8.0	8	8.2	8	8.2
Grade 3	0	-	0	-	0	-	0	-	0	-	0	-

N =  Number of subjects with at least one symptom sheet completed; n/% =  number and percentage of subjects reporting a specified symptom.

In both groups the most common solicited local symptom was pain at the injection site. There was no apparent trend in incidence of either pain or swelling with subsequent doses of RTS,S/AS02_D_ or *Engerix-B*™.

#### Solicited AEs after TETRActHib™ vaccinations

Five children (4 in the *Engerix-B*™ and 1 in the RTS,S group) experienced grade 3 solicited general symptoms following either the first or the second *TETRActHib*™ dose (Table 2). All of these events were considered to be related to vaccination and the children fully recovered. None of the solicited local symptoms were reported to be of grade 3 intensity.

**Table 2:** Incidence of solicited general symptoms by dose within the 7-days follow-up after *TETRActHib™* according to randomization group.

	After dose 1				After dose 2				After dose 3			
	RTS,S/AS02_D_		*Engerix -B*		RTS,S/AS02_D_		*Engerix -B*		RTS,S/AS02_D_		*Engerix -B*	
	(N = 107)		(N = 107)		(N = 102)		(N = 104)		(N = 100)		(N = 100)	
	n	%	n	%	N	%	n	%	n	%	n	%
**General symptoms**												
Drowsiness												
Any	31	29.0	25	23.4	29	28.4	27	26.0	28	28.0	30	30.0
Related	12	11.2	12	11.2	3	2.9	8	7.7	5	5.0	3	3.0
Grade 3	0	-	1	0.9	0	-	0	-	0	-	0	-
Grade 3 related	0	-	1	0.9	0	-	0	-	0	-	0	-
Fever												
Any	10	9.3	13	12.1	6	5.9	12	11.5	8	8.0	5	30.0
Related	10	9.3	13	12.1	6	5.9	12	11.5	8	8.0	5	3.0
Grade 3	0	-	1	0.9	1	1.0	1	1.0	0	-	0	-
Grade 3 related	0	-	1	0.9	1	1.0	1	1.0	0	-	0	-
Irritability												
Any	59	55.1	59	55.1	44	43.1	48	46.2	49	49.0	50	50.0
Related	40	37.4	47	43.9	18	17.6	29	27.9	26	26.0	23	23.0
Grade 3	0	-	1	0.9	0	-	0	-	0	-	0	-
Grade 3 related	0	-	1	0.9	0	-	0	-	0	-	0	-
Loss of appetite												
Any	22	20.6	15	14.0	26	25.5	20	19.2	27	27.0	30	30.0
Related	3	2.8	2	1.9	0	-	1	1.0	4	4.0	3	3.0
**Local symptoms**												
Pain												
Any	105	98.1	103	96.3	98	96.1	102	98.1	92	92.0	93	93.0
Grade 3	0	-	0	-	0	-	0	-	0	-	0	-
Swelling												
Any	22	20.6	18	16.8	14	13.7	22	21.2	16	16.0	22	22.0
Grade 3	0	-	0	-	0	-	0	-	0	-	0	-

N =  Number of subjects with at least one symptom sheet completed; n/% =  number and percentage of subjects reporting a specified symptom.

Pain at the injection site was the most frequently reported solicited local symptom. The incidence of pain and swelling was similar in both vaccine groups. There was no apparent trend in incidence of either pain or swelling with subsequent doses of *TETRActHib*™.

#### Unsolicited adverse events

Unsolicited AEs occurring within 30 days following vaccination were reported by 86.9% of participants in both vaccine groups. In both groups, the most frequently reported diagnosis was upper respiratory tract infection (49.5% of subjects in the RTS,S/AS02_D_ and 45.8% of subjects in the *Engerix-B*™ group) (data not shown).

No unsolicited AE was considered to be causally related to the study vaccines.

Grade 3 unsolicited events were rare, occurring with similar frequency in both comparison groups. Five subjects (4.7%) reported ten grade 3 unsolicited AEs in the RTS,S/AS02_D_ group [anemia (4), bronchopneumonia (1), *P. falciparum* infection (4), pneumonia (1)] and seven subjects (6.5%) reported twelve grade 3 unsolicited AEs in the *Engerix-B*™ group [anaemia (1), conjunctivitis (1), pyrexia (1), bronchitis (1), bronchopneumonia (1), skin furuncle (1), gastroenteritis (3), pneumonia (2), bronchospasm (1)] (data not shown).

#### SAEs

There were 69 children with at least one SAE (35 in the RTS,S/AS02_D_ and 34 in the *Engerix-B*™ group) as shown in Table 3. The proportion of subjects reporting an SAE was similar in the RTS,S/AS02_D_ (32.7%, 95% CI 24.0–42.5) and the control group (31.8%, 95% CI 23.1–41.5). None of them were considered to be related to vaccination. The total number of SAEs classified according to the Medical Dictionary for Regulatory Activities (MedDRA)[19] preferred terms was 157 (75 in the RTS,S/AS02_D_ and 82 in the control group).

**Table 3:** Percentage of participants reporting SAEs classified by MedDRA primary organ class and preferred term over 14 months follow-up.

	*Engerix-B* (N = 107)			RTS,S/AS02_D_ (N = 107)		
	n	%	95% CI	n	%	95% CI
Number of subjects with at least one SAE reported	34	31.8	23.1–41.5	35	32.7	24.0–42.5
Number of SAEs reported classified by MedDRA preferred term*	82	76.6	67.5–84.3	75	70.1	60.5–78.6

N  =  number of subjects with at least one administered dose and included in ITT cohort.

n/%  =  number/percentage of subject reporting at least once the symptom.

*Symptoms reported by a subject after a given dose and classified by the same Preferred Term are counted once.

During the entire follow-up period, 15 participants in the RTS,S/AS02_D_ group reported *P. falciparum* as an SAE requiring hospitalization, corresponding to 14.0% (95% CI 8.1–22.1). In the control group, there were 13 participants hospitalized with malaria, corresponding to 12.1% (95% CI 6.6–19.9). All cases fully recovered. The other main diagnoses of SAEs requiring admission were anaemia (15.9% vs 12.1%) gastroenteritis (12.1% vs 16.1%) and pneumonia (8.4% vs 7.5%) in the RTS,S/AS02_D_ and *Engerix-B*™ groups, respectively (data not shown).

Four deaths occurred during follow-up (two in each group). None of the deaths was judged to be related to vaccination. In the RTS,S/AS02_D_ group, an eight month old girl died at home four months after having received a study vaccine. The presumptive diagnosis based on the verbal autopsy obtained from the mother was staphylococcal septic shock (recorded history of fever, generalised vesicular eruption, skin peeling and face swelling prior to death).

The second death in this group also occurred at home nine months after the child had received the last study vaccination. The 15 month HIV negative old boy had previously been admitted for a *Streptococcus pneumoniae* pneumonia (confirmed by a positive blood culture) and anaemia. According to the verbal autopsy, the death occurred after about 3 weeks of fever, vomiting, diarrhoea, abdominal pain and pallor. The parents did not seek treatment at any health facility. The final diagnosis following verbal autopsy review was chronic gastroenteritis with severe dehydration.

In the *Engerix-B*™ group, a 10 month old boy died at home six months after receiving the third dose of the vaccine. The child had been seen by a field worker 3 days before he died and he appeared to be in good health. The verbal autopsy performed to the mother revealed that 24 hours prior to the fatal event the child abruptly started with intense vomiting and diarrhoea. The mother took him to a traditional healer who administered him “traditional medication”. The child died shortly after. The probable cause of death was severe dehydration from gastroenteritis. The possibility of an adverse effect secondary to traditional medicine ingestion could not be excluded.

The second death was of an 11 month old girl, who died at home 7 months after the last vaccination with *Engerix-B*™. According to the child's father, the child had diarrhoea and fever for 4 days, stopped eating, and progressively developed sunken eyes and pallor. The child was not brought to the health centre. Clinician's review of the verbal autopsy report concluded that the most probable cause of death was severe dehydration secondary to gastroenteritis.

#### Monitoring of hematological and biochemical parameters

Hematological values outside the normal range were infrequent. The majority of abnormal values of hemoglobin, white blood cells and platelets were of grade 1 intensity and occurred with similar incidence in the two groups. One child with concomitant malaria in the RTS,S/AS02_D_ group had a low platelet count (44×10^9^/L) of grade 2 intensity one month after the last vaccination. This value was within the normal range (167×10^9^/L) at month 6.

Biochemistry values outside the normal range were also infrequent. The majority of out of range ALT and bilirubin values were of grade 1 intensity, occurring with a similar incidence in the two groups. One participant in the RTS,S/AS02_D_ group had a grade 2 ALT value one week after the first dose (162 µmol/L) which dropped to 38 µmol/L one month after the third dose and to 39 µmol/L by study month 6. No creatinine values were outside the normal ranges.

### Vaccine immunogenicity

ATP analysis of vaccine immunogenicity at month 14 included 151 children (73 in the RTS,S/AS02_D_ and 78 in the control group). The anti-CS antibody GMTs declined from 199.9 EU/mL one month post dose 3 to 58.8 EU/mL and 7.3 EU/mL by 3.5 and 12 months post dose 3 respectively in the RTS,S/AS02_D_ group. In the control group, anti-CS antibody GMTs were below the assay cut off (0.5 EU/mL) at all post vaccination time points.

In the RTS,S/AS02_D_ group, the anti-HBs antibody GMTs declined from 10082 mIU/mL one month after dose 3 to 2751 mIU/mL by 12 months post dose 3. In the *Engerix-B*™ group, the anti-HBs GMTs were 392.4 mIU/mL and 263.9 mIU/mL at the same time points. All children of both RTS,S/AS02_D_ and control groups were seroprotected for Hepatitis B at 12 months post dose 3.

### Vaccine efficacy

Results of VE analyzed over three different time periods are summarized in Table 4.

**Table 4:** Vaccine efficacy evaluated for different follow-up periods.

	*Engerix B* (n = 92)				RTS,S/AS02_D_ (n = 93)		Vaccine Efficacy		
	Events	PYAR	Rate	Events	PYAR	Rate		95% CI	p
ATP_(3–9)_									
First or only (FO) episode of fever and parasitemia >500/µl	34	31.5	1.08	21	38.2	0.55	48.8%	11.3–70.4	0.017
FO episode of fever or history of fever* and parasitemia >0/µl	48	27.7	1.74	29	36.4	0.80	54.5%	27.3–71.5	0.001
Multiple episodes of fever and parasitemia>500/µl	45	36.2	1.24	23	40.2	0.57	53.7%	21.4–72.7	0.004
Multiple episodes of fever or history of fever* and parasitemia >0/µl	72	36.0	2.00	34	40.0	0.85	58.9%	35.8–73.6	<0.001
ATP_(3–14)_									
First or only (FO) episode of fever and parasitemia >500/µl	45	51.3	0.88	36	61.7	0.58	33.0%	−4.3–56.9	0.076
FO episode of fever or history of fever* and parasitemia >0/µl	57	41.4	1.38	45	57.1	0.79	41.9%	13.7–60.9	0.007
Multiple episodes of fever and parasitemia>500/µl	74	68.9	1.07	58	72.5	0.80	25.9%	−15.7–52.6	0.187
Multiple episodes of fever or history of fever* and parasitemia >0/µl	120	68.4	1.75	85	72.3	1.18	35.1%	2.2–57.0	0.039
ITT**_(0–14)_									
First or only (FO) episode of fever and parasitemia >500/µl	54	79.4	0.68	46	90.2	0.51	25.9%	−9.9–50.0	0.136
Multiple episodes of fever and parasitemia>500/µl	105	111	0.94	82	113	0.72	24.3%	−12.9–49.2	0.173
Multiple episodes of fever and parasitemia>500/µl (PCD only)	95	112	0.85	80	113	0.71	17.6%	−24.2–45.3	0.355

*History of fever in previous 24 hours.

**ITT: n = 107 for each group.

PYAR =  Persons-years at risk. Vaccine efficacy adjusted estimates for area and distance from health center (km).

ATP =  According to the Protocol; ITT =  Intention to Treat; PCD =  Passive Case Detection.

It should be noted that the trial was not powered for VE against clinical malaria and all analyses herein are exploratory.

VE analysis between months 3 to 9 of follow-up (ATP_3–9_) was 48.8% (95% CI 11.3–70.4, p = 0.017) against first or only clinical episodes and 53.7% (95% CI 21.4–72.7, p = 0.004) against multiple episodes.

**Figure 2:**
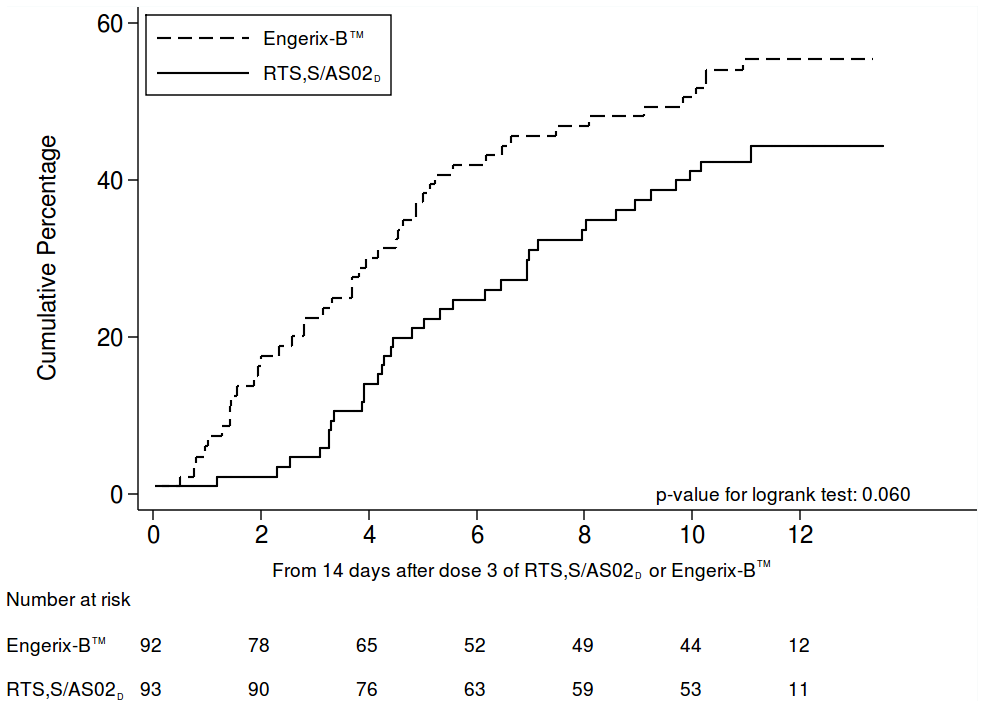
Kaplan-Meier curves for the cumulative proportion of children with at least one episode of clinical malaria between study months 3 to14 (ATP _3–14_).

VE against first or only episodes of clinical malaria over the entire follow-up period up to month 14 (ATP_3–14_) was 33.0% (95% CI -4.3–56.9, p = 0.076) and VE against multiple malaria episodes was 25.9% (95% CI -15.7–52.6, p = 0.167). Figure 2 shows Kaplan-Meier curves of the cumulative incidence of first or only episodes of clinical malaria in both groups. A test based on the Schoenfeld residuals (p = 0.049) suggested that the hazard was not proportional over the follow-up period, consistent with the notion that VE waned over the course of the study.

Analysis of the relationship between anti-CS antibody levels and VE against clinical malaria suggested that within RTS,S recipients, the hazard rates of disease per 2 fold increase anti-CS titres at one month post dose 3 were significantly reduced by 84.1% (95% CI 43.5–95.5, p = 0.004) and 72.4% (95% CI 35.1–88.2, p = 0.003) for the two follow-up periods (ATP_3–9_ and ATP_3–14_), respectively.

## Discussion

This is the first comprehensive safety and reactogenicity report of RTS,S/AS malaria vaccine in infants. We previously reported that VE against new *P. falciparum* infections was 65.9% during the initial three months after dose 3 in infants immunized in a staggered schedule with routine EPI vaccines [10]. This report goes further to include an exploratory analysis of VE against clinical malaria observed during the study.

The RTS,S/AS02_D_ reactogenicity profile was similar to that recorded in previous trials in older age groups [2], [5]. The safety profile in infants remained promising during the extended follow-up, with no significant differences between groups in the frequency of SAEs. No safety signals were found in the monitoring of hematologic and biochemistry data.

We observed no strong evidence of significant differences in the immunological responses to vaccination with RTS,S/AS02_D_ in infants in this trial compared to older children. Anti-CS antibody titters decayed over time. However, there is no evidence that they did so more precipitously in this trial compared to other pediatric trials, where antibody decay profiles are consistent with a half-life of 6 to 8 weeks. In this trial, anti-CS responses at 12 months post-dose 3 remain 15 fold higher in the RTS,S/AS02_D_ group than in the control group. Where longer follow-up data are available, low but persistently elevated anti-CS responses have been reported (10–30 times higher than in controls) also [6]. This is consistent with the induction of long term T cell and B cell memory responses by RTS,S/AS02. Such persistence of antibody responses is likely to be seen in this infant population but this can only be confirmed by longer follow-up as planned in the ongoing Phase 3 trials.

Anti-HBs responses were higher throughout the follow-up in recipients of RTS,S/AS02_D_ than of the licensed Hepatitis B control vaccine probably reflecting the use of a different adjuvant system. HBs antibody titters also decayed over time. However, all children vaccinated with both RTS,S/AS02_D_ and *Engerix-B*™ vaccines reached seroprotection levels for anti-HBs 12 months post Dose 3.

Vaccine efficacy against clinical malaria over the 12 months follow-up period after dose three was 33% (95% CI -4.3–56.9, p = 0.076), whereas during the initial 3.5 months of double-blind follow-up the efficacy was 65.8% (95% CI 25.3–84.4, p = 0.007). This difference could be due to chance as the confidence intervals of the two estimates overlap, and the study is underpowered for such analyses. Nevertheless, together with the data showing that the hazard was not proportional over the follow-up periods, the results suggest that VE against clinical malaria may have waned over the 14 months follow-up period.

Caution is needed when attempting to compare the results of this study with data reported from a previous phase IIb trial conducted in this same area among children aged 1 to 4 years [5]. Cohort 2 of that trial had a very similar design to the infant study that we are reporting, including the administration of presumptive treatment with effective antimalarials between dose 2 and 3 and an initial follow-up through intense active detection of infections (ADI). In both studies, VE against clinical malaria appeared to wane over time [20]. This is in sharp contrast to cohort 1 of the phase IIb trial where children were only followed-up by passive case detection and did not have presumptive treatment. Among these children VE persisted at 30% for 45 months [6]. Reasons for this apparent differences in the duration of protection are discussed elsewhere [20].

While several previous trials have shown a relationship between anti-CS antibody responses and risk of malaria infection, this study provides the first evidence of a similar relationship between anti-CS antibodies and protection against clinical malaria. It is probable that in trials in older populations similar analyses have been confounded by the superimposed naturally acquired immunity.

In summary, these results confirm the good safety and immunogenicity profile of RTS,S/AS02_D_ malaria vaccine in African infants, as well as confirm protection against clinical malaria for at least one year. Together they support the rationale for the ongoing Phase III trial.

## Supporting Information

Protocol S1 Trial Protocol
(0.92 MB PDF)

Checklist S1 CONSORT Checklist
(7.60 MB RTF)
