# Supplementary material for: Safety, Immunogenicity and Duration of Protection of the RTS,S/AS02D Malaria Vaccine: One Year Follow-Up of a Randomized Controlled Phase I/IIb Trial
Source: PLoS One. 2010 Nov 4;5(11):e13838. doi: 10.1371/journal.pone.0013838 (PMC2973956; doi:10.1371/journal.pone.0013838)
Supplement: Protocol S1 — Trial Protocol (0.92 MB PDF) [file pone.0013838.s001.pdf]

GlaxoSmithKline Biologicals
Rue de l'Institut 89, 1330 Rixensart,
Belgium.

**Study vaccines**	GlaxoSmithKline Biologicals' candidate *Plasmodium falciparum* malaria vaccine RTS,S/AS02D, GlaxoSmithKline Biologicals hepatitis B vaccine, Engerix-B® and Aventis Pasteur's DTPw/Hib vaccine TETRActHib™
**eTrack study number**	103967
**eTrack abbreviated title**	Malaria-038
**Investigational New Drug (IND) number**	BB-IND 10514
**Date of approval**	04 November 2004 (Final)
**Amendment 1**	17 January 2005 (Final)
**Amendment 2**	12 April 2005 (Final)
Amendment 3	***29 June 2005 (Final)***
**Title**	A Phase I/IIb randomized, double-blind, controlled study of the safety, immunogenicity and proof-of-concept of RTS,S/AS02D, a candidate malaria vaccine in infants living in a malaria-endemic region

GlaxoSmithKline Biologicals

Co-ordinating author Conor Cahill, Scientific Writer.

Contributing authors

- Ripley Ballou, Vice President – Clinical Research & Development, Early Development
- Ozzie Berger, US Regulatory Affairs
- Adelheid Davis, Local GSK Monitor, South Africa
- Joe Cohen, Director Research & Development – Vaccines For Emerging Diseases Program
- ***Sabine Corachan, Central Study Coordinator (Amended 29 June 2005)***
- Marie Claude Dubois, Project Manager – Disease Area Programme Vaccines for Emerging Diseases
- Marguerite Koutsoukos, Scientist Clinical Immunology Manager, Disease Area Programme Vaccines for Emerging Diseases
- Amanda Leach, Clinical Development Manager
- Phillipe Moris, Scientist – TP Human Cellular Immunology, Vaccines for emerging diseases
- Andreas Pakendorf, Local GSK Monitor, South Africa
- Bart Spiessens, Statistician
- Joelle Thonnard, Manager, Post Marketing Surveillance
- Marie Chantal Uwamwezi, Scientist – Regulatory Development Unit III
- Laurence Vigneron, Central Study Coordinator

**eTrack study number**	103967
**eTrack abbreviated title**	Malaria-038
**IND number**	BB-IND 10514
**Date of approval**	04 November 2004 (Final)
**Amendment 1**	17 January 2005 (Final)
**Amendment 2**	12 April 2005 (Final)
Amendment 3	***29 June 2005 (Final)***
**Title**	A Phase I/IIb randomized, double-blind, controlled study of the safety, immunogenicity and proof-of-concept of RTS,S/AS02D, a candidate malaria vaccine in infants living in a malaria-endemic region

Centro de Investigação em Saúde de Manhiça (CISM), Mozambique and Hospital Clinic (University of Barcelona), Spain

Contributing Authors

- Pedro Aide, Clinician
- Pedro Alonso, Director
- John Aponte, Medical Epidemiologist
- ***Arnoldo Barbosa Immunologist (Amended 29 June 2005)***
- Enrique Bassat, Pediatrician
- ***Anna Berenguera (Amended 29 June 2005)***
- Carlota Dobaño, Immunologist
- Mateu Espasa, Laboratory Scientist
- Caterina Guinovart, Epidemiologist
- Antonio Langa, Data Manager
- Eusebio Macete, Clinician
- Inacio Mandomando, Laboratory Scientist
- ***Denise Naniche, Immunologist (Amended 29 June 2005)***
- Margarita Navia, Project Manager
- ***Montse Renom, Project Manager (Amended 12 April 2005)***.
- Jahit Sacarlal, Clinician
- Ricardo Thompson, Epidemiologist

Malaria Vaccine Initiative at Program for Appropriate Technology in Health

Contributing Author

- Jessica Milman, Program Officer

GlaxoSmithKline Biologicals will act as sponsor for this trial

**eTrack study number**	103967
**eTrack abbreviated title**	Malaria-038
**IND number**	BB-IND 10514
**Date of approval**	04 November 2004 (Final)
**Amendment 1**	17 January 2005 (Final)
**Amendment 2**	12 April 2005 (Final)
Amendment 3	***29 June 2005 (Final)***
**Title**	A Phase I/IIb randomized, double-blind, controlled study of the safety, immunogenicity and proof-of-concept of RTS,S/AS02D, a candidate malaria vaccine in infants living in a malaria-endemic region

~~Sponsor signatory approval~~

**~~Sponsor signatory:~~**	~~Amanda Leach~~<br>~~Clinical Development Manager~~
**~~Signature:~~**	______________________________
**~~Date:~~**	______________________________

Sponsor Information

eTrack study number 103967

eTrack abbreviated title Malaria-038

IND number BB-IND 10514

1. Principal Investigator

Pedro Alonso MD,
Centro de Investigação Em Saude Da Manhiça,
Scientific Director,
Manhiça, Moçambique,
CP 1929, Maputo,
Mozambique.
Tel: +258.1.81.00.02 / 01.81
Fax/Phone: +258.1.81.00.02
email: palonso@clinic.ub.es

2. Medical Monitor

Amanda Leach,
Clinical Development Manager,
GlaxoSmithKline Biologicals,
Rue de l'Institut 89, 1330 Rixensart,
Belgium.
Tel: +32.2.656.77.88
Fax: +32.2.656.61.60
email: amanda.leach@gskbio.com

3. Central Study Coordinator

Sabine Corachan,
Central Study Coordinator,
GlaxoSmithKline Biologicals,
Rue de l'Institut 89, 1330 Rixensart,
Belgium.
Tel: +32.2.656.92.33
Fax: +32.2.656.61.60
email: sabine.corachan@gskbio.com

eTrack study number 103967

eTrack abbreviated title Malaria-038

IND number BB-IND 10514

4. Study Contacts for Reporting of a Serious Adverse Event

Central Study Coordinator

Sabine Corachan,
Central Study Coordinator,
GlaxoSmithKline Biologicals,
Rue de l'Institut 89, 1330 Rixensart,
Belgium.
Tel: +32.2.656.92.33
Fax: +32.2.656.61.60
email: sabine.corachan@gskbio.com

Local GSK Study Monitor

~~Andreas Pakendorf &~~ Adelheid Davis,
GlaxoSmithKline,
Private Bag X173,
Bryanston 2021,
South Africa.
Tel: +27.11.745.63.24
Fax: +27.11.745.74.95
email: ~~andreas.m.pakendorf@gsk.com &~~
adelheid.a.davis@gsk.com

(Amended 29 June 2005)

Malaria Vaccine Initiative

Filip Dubovsky,
6290 Montrose Road, Rockville,
Maryland 20852,
USA.
Tel: +1.301.770.53.77
Fax: +1.301.770.53.22
email: sae@malariavaccine.org

alternate email:
fdubovsky@malariavaccine.org

Jessica Milman,
6290 Montrose Road, Rockville,
Maryland 20852,
USA.
Tel: +1.301.770.53.77
Fax: +1.301.770.53.22
email: sae@malariavaccine.org

alternate email:
jmilman@malariavaccine.org

Back-up Study Contact at GSK Biologicals for Reporting Serious Adverse Events
Manager Clinical Safety Vaccines

GSK Biologicals Clinical Safety Physician,
GlaxoSmithKline Biologicals,
Rue de l'Institut 89, 1330 Rixensart,
Belgium.
Tel: +32.2.656.87.98
Fax: +32.2.656.80.09
Mobile phone for 7/7 day availability: +32.477.40.47.13

eTrack study number 103967

eTrack abbreviated title Malaria-038

IND number BB-IND 10514

5. Study Contact for Emergency Code Break

GSK Biologicals Clinical Safety Physician,
GlaxoSmithKline Biologicals,
Rue de l'Institut 89, 1330 Rixensart,
Belgium.
Tel: +32.2.656.87.98
Fax: +32.2.656.80.09
Mobile phone for 7/7 day availability: +32.477.40.47.13

6. Study Center

Centro de Investigação em Saúde de Manhiça (CISM), Mozambique.

Investigator Agreement

**eTrack study number**	103967
**eTrack abbreviated title**	Malaria-038
**IND number**	BB-IND 10514
**Title**	A Phase I/IIb randomized, double-blind, controlled study of the safety, immunogenicity and proof-of-concept of RTS,S/AS02D, a candidate malaria vaccine in infants living in a malaria-endemic region

I agree:

- To assume responsibility for the proper conduct of the study at this site.
- To conduct the study in compliance with this protocol, any mutually agreed future protocol amendments, and with any other study conduct procedures provided by GlaxoSmithKline Biologicals (GSK Biologicals).
- Not to implement any changes to the protocol without agreement from the sponsor and prior review and written approval from the Institutional Review Board (IRB) or Independent Ethics Committee (IEC), except where necessary to eliminate an immediate hazard to the subjects, or for administrative aspects of the study (where permitted by all applicable regulatory requirements).
- That I am thoroughly familiar with the appropriate use of the vaccine(s), as described in this protocol, and any other information provided by the sponsor, including, but not limited to, the following: the current Investigator's Brochure (IB) or equivalent document, IB supplement (if applicable), prescribing information (in the case of a marketed vaccine) and/or Master Data Sheet (if the Master Data Sheet exists and serves as reference document for the vaccine in the case of a marketed vaccine).
- That I am aware of, and will comply with, "Good Clinical Practices" (GCP) and all applicable regulatory requirements.
- That I have been informed that certain regulatory authorities require the sponsor to obtain and supply, as necessary, details about the investigator's ownership interest in the sponsor or the investigational product, and more generally about his/her financial ties with the sponsor. GSK Biologicals will use and disclose the information solely for the purpose of complying with regulatory requirements.

Hence I:

- Agree to supply GSK Biologicals with any necessary information regarding ownership interest and financial ties (including those of my spouse and dependent children).
- Agree to promptly update this information if any relevant changes occur during the course of the study and for 1 year following completion of the study.

- Agree that GSK Biologicals may disclose any information it has about such ownership interests and financial ties to regulatory authorities.
- Agree to provide GSK Biologicals with an updated Curriculum Vitae and other FDA required documents.

~~Investigator name:~~ ~~Pedro Alonso MD~~

~~Investigator signature~~	~~Date~~

Synopsis

**Title**	A Phase I/IIb randomized, double-blind, controlled study of the safety, immunogenicity and proof-of-concept of RTS,S/AS02D, a candidate malaria vaccine in infants living in a malaria-endemic region
**Study Population**	Healthy male and female infants aged 6 to 12 weeks at enrollment. Those determined to be eligible, based on the inclusion and exclusion criteria will be enrolled in the study.
**Rationale**	The RTS,S/AS02 candidate malaria and hepatitis B vaccine consists of sequences of the circumsporozoite (CS) protein and hepatitis B surface antigen (HBsAg) adjuvanted with AS02 (proprietary oil-in-water emulsion, MPL® and QS21 immunostimulants). The vaccine is being developed for the routine immunization of infants and children living in malaria endemic areas. This vaccine would offer protection against malaria disease due to the parasite *Plasmodium falciparum*. The vaccine would also provide protection against infection with hepatitis B virus (HBV).

This study is being initiated following the conclusive demonstration of efficacy in the trial of RTS,S/AS02A in children aged 1 to 4 years in Mozambique. The vaccine demonstrated 30% (95% CI 11% to 45%) protection against clinical malaria disease, 58% (95% CI 16% to 81%) against severe malaria disease and 45.0% (31% to 56%) protection against infection.

RTS,S/AS02D (0.5 mL dose) is composed of the same active constituents in the same quantities as in a 0.25 mL dose of RTS,S/AS02A. A bridging study was conducted comparing RTS,S/AS02D to RTS,S/AS02A. Non-inferiority of the anti-CS and anti-HBs immune response was demonstrated. The safety profile of RTS,S/AS02D was comparable to that of RTS,S/AS02A.

RTS,S/AS02D is being developed to be integrated into the Expanded Program on Immunization (EPI) in malaria endemic regions. This trial is the first administration of RTS,S/AS02D in infants. The primary objective of this study is to assess vaccine safety in young infants. It will also provide key development information on the comparison of the hepatitis B response induced by RTS,S/AS02D compared to a licensed hepatitis B vaccine, and proof-of-concept of efficacy. This product development plan is conducted under a partnership agreement with the Malaria Vaccine Initiative at PATH (MVI) and is guided by a joint MVI/GSK Steering Committee.

Objectives **Primary; Safety**

- To describe the safety and reactogenicity of RTS,S/AS02D administered as 3 doses intramuscularly in the left thigh to infants at 10, 14 and 18 weeks of age, staggered with the administration of 3 doses of TETRActHib intramuscularly in the right thigh at 8, 12 and 16 weeks of age.

- **Secondary; Immunogenicity**
- To demonstrate the non-inferiority of antibody responses to Hepatitis B, when administered as 3 doses of RTS,S/AS02D at 10, 14 and 18 weeks of age compared to the regimen of 3 doses of Engerix-B at the same ages.

- To describe antibody responses to the circumsporozoite (CS) antigen of the RTS,S/AS02D malaria candidate vaccine administered as 3 doses intramuscularly in the left thigh to infants at 10, 14 and 18 weeks of age

- **Secondary; Proof-of-concept**
- To assess the efficacy of RTS,S/AS02D against infection (defined as *P. falciparum* asexual parasitemia > 0) with *P. falciparum* malaria in infants immunized with RTS,S/AS02D as 3 doses at 10, 14 and 18 weeks of age

Study Design

- Phase I/IIb, single-center, double blind (observer blind, participant blind), randomized controlled trial with two groups in one study site.

Vaccine	Age (weeks)	Number of infants to be enrolled	Estimated number evaluable
TETRActHib	8, 12, 16	110	100
RTS,S/AS02D	10, 14, 18		
TETRActHib	8, 12, 16	110	100
Engerix-B	10, 14, 18		

- Two hundred and twenty children will be enrolled.

- Prior to study start, a community information program will inform the local population of the study. Throughout the period of enrollment study information will be presented at antenatal clinics to expectant mothers.
- Infants at high risk of vertical transmission of hepatitis B infection will be excluded from this trial of an experimental hepatitis B vaccine. These infants will receive a licensed hepatitis B vaccine in a schedule beginning at birth. The woman's full signed informed consent will be required for antenatal HBsAg testing.
- The HIV infection rates of women presenting to antenatal clinics in the Manhiça area is currently approximately 20%. Infants of HIV positive women will be excluded to avoid obscuring the safety pattern associated with the investigational vaccine. The woman's full signed informed consent will be required for antenatal HIV testing.
- Healthy male and female infants aged 6 to 12 weeks will be screened. Those determined to be eligible, based on the inclusion and exclusion criteria, will be enrolled in the study.
- Infants enrolled for the study attending for the first injection of DTPw will be identified at EPI clinics and, if they meet the inclusion criteria, their parent(s)/guardian(s) asked for consent to the trial.
- Route of administration: all vaccines will be administered by the intramuscular route to the antero-lateral thigh.

  Left thigh: RTS,S/AS02D or Engerix-B

  Right thigh: TETRActHib

- Each infant will be observed for at least 60 minutes after vaccination with RTS,S/AS02D or Engerix-B to evaluate and treat any acute adverse events
- Oral Polio Vaccine will be provided and administered according to the recommended national guidelines (at birth, and coadministered with TETRActHib at 8, 12 and 16 weeks during the study).
- After vaccination, trained field workers will visit the children to detect adverse events (AEs) occurring after vaccination for one week (days 1, 2, 3, 4, 5 and day 6 after each vaccination). Data will be collected after all doses of RTS,S/AS02D or Engerix-B and all doses of TETRActHib. Diary cards and thermometers will be provided for the field workers to record axillary temperature and any local (at the injection site) or general adverse events.

- There will be a 14 day follow-up period after each Dose of TETRActHib and Dose 1 and Dose 2 of RTS,S/AS02D or Engerix-B, and a one month (30 day) follow-up period after Dose 3 of RTS,S/AS02D or Engerix-B for reporting unsolicited symptoms
- Infants will be followed for 14 months after administration of the first dose of TETRActHib.
- Recording of serious adverse events will be throughout the study period. They will be captured through the morbidity surveillance system at Ilha Jossina Health Center***, Taninga Health Center*** and Manhiça District Hospital. In addition all enrolled children will be visited at home monthly by field workers until study conclusion to ensure complete identification of all SAEs ***(Amended 29 June 2005)***.
- Bloods for safety monitoring of hematology, renal and hepatic function will be measured at 1 week post Dose 1, one month post Dose 3, 3½ months post Dose 3 and 12 months post Dose 3 of RTS,S/AS02D or Engerix-B.
- Non inferiority of hepatitis B response will be determined one month post Dose 3 of RTS,S/AS02D or Engerix-B.
- Anti-CS antibody titers will be determined at baseline, one month post Dose 3, 3½ months post Dose 3 and 12 months post Dose 3 of RTS,S/AS02D or Engerix-B.
- Anti-HBs antibody titers will be determined at baseline, one month post Dose 3 and 12 months post Dose 3 of RTS,S/AS02D or Engerix-B.
- Two weeks prior to Dose 3 of ~~TETRActHib~~ ***RTS,S/AS02D***, children will be treated with sulfadoxine-pyrimethamine and Amodiaquine for presumptive clearance of parasitemia ***(Amended 12 April 2005)***.
- All children will have a blood slide prepared and read to check for asexual *P. falciparum* parasitemia on the day of receiving Dose 3 of RTS,S/AS02D. Any children who test positive will be excluded from ADI.
- The total period of surveillance for ADI is 12 weeks. Seven visits will contribute to the ADI, made up of four field-worker visits and three clinic visits.
- At each contact for ADI, history of fever will be recorded and axillary temperature taken; a blood sample will be taken for malaria parasite genotyping and a smear will be taken for detection of malaria parasites.

- Subjects who are symptomatic at the time of ADI contact (i.e. history of fever within previous 24 hours or axillary temperature >37.5°C) will have a blood slide read and treated within the same day if the blood slide is positive.
- Infants will be followed passively for the occurrence of clinical malaria for the duration of their participation in the study. Cases will be detected at all health facilities in the study area.
- Infection with malaria parasites is defined as *P. falciparum* asexual parasitemia > 0
- Data collection will be by conventional CRF.
- Duration of the study will be 14 months per subject.
- The final analysis of the study for all primary and secondary endpoints will take place on all data collected up to the end of the surveillance for ADI (Clinic Visit 11)
- All children will be followed for safety for a period that extends to 12 months post Dose 3. All data collected after the Final Analysis (Clinic Visit 11) will be reported in an Annex Report.
- This study is overseen by a formally constituted DSMB operating under a charter. During the vaccination phase of the study full safety reports will be sent to the DSMB at 3 points corresponding to; after 30, 60 and 100 subjects have been enrolled and have completed the first 7 days of follow-up post Dose 1 of RTS,S/AS02D or Engerix-B.
- This is the first administration of RTS,S/AS02D to infants and therefore the sample size of this Phase 1/2 safety study is limited to 200. Therefore only large differences in the frequencies of SAEs can be detected with reasonable power. The data set will be examined, comparing the rates of SAEs at the Medical Dictionary for Regulatory Activities (MedDRA) preferred term level. A sample size of 200 has the power to detect differences in the rates of safety endpoints (SAEs at the MedDRA preferred term level) as shown in the table below:

Frequency of events for Engerix-B	Frequency of events for RTS,S/AS02D	Power to detect difference (number per group equals 100)
1%	12%	80%
3%	15%	80%
5%	19%	80%
10%	26%	80%

- This sample size will have 90% power at a 2.5% significance level using a one-sided equivalence test of proportions when the proportion that are seroprotected in the control group (subjects vaccinated with Engerix-B) is at least 0.97 and the proportion in the RTS,S/AS02D group is 0.97 and the maximum allowable difference between these proportions that still results in equivalence (the range of equivalence) is 0.10 (PASS 2000, one sided equivalence test of proportions, alpha = 2.5%).
- Assuming an attack rate of 75% in the Control Group over the 3-month surveillance period, 100 evaluable subjects per group are needed in order to have 90% power to show a Vaccine Efficacy (=1-hazard ratio) of 45%. This is equivalent to an attack rate in the Vaccinated Group of 53.3%. Using proportion of subjects with malaria infection over the surveillance period, a Vaccine Efficacy (=1-risk ratio) of 29% can be detected with 90% power.

Number of subjects

The necessary number of infants will be screened to enroll approximately 220 infants to the study. It is anticipated that approximately 10% of enrolled infants will drop out of the study, resulting in approximately 200 subjects evaluable.

Primary endpoint

Safety

- Occurrence of SAEs from the time of first TETRActHib vaccination until month 6 post Dose 1

Secondary endpoints

Safety

- Occurrence of unsolicited AEs after Dose 1, 2 and 3 of TETRActHib over a 14 day follow-up period (day of vaccination and 13 subsequent days)
- Occurrence of unsolicited AEs after Dose 1 and 2 of RTS,S/AS02D or Engerix-B over a 14 day follow-up period (day of vaccination and 13 subsequent days)
- Occurrence of unsolicited AEs after Dose 3 of RTS,S/AS02D or Engerix-B over a 30 day follow-up period (day of vaccination and 29 subsequent days)
- Occurrence of solicited general and local reactions over a 7 day follow-up period (day of vaccination and 6 subsequent days) after TETRActHib
- Occurrence of solicited general and local reactions over a 7 day follow-up period (day of vaccination and 6 subsequent days) after RTS,S/AS02D or Engerix-B.

Immunogenicity

Endpoints assessed one month post Dose 3 of RTS,S/AS02D or Engerix-B; assessment of non-inferiority:

- Anti-HBs antibody titers; difference between groups in percent seroprotection <10%

Endpoints assessed prior to vaccination, 1 month post Dose 3 of RTS,S/AS02D or Engerix-B:

- Anti HBs antibody titers

Endpoints assessed prior to vaccination, 1 month post Dose 3, 3½ months post Dose 3 of RTS,S/AS02D or Engerix-B:

- Anti-CS antibody titers.

Endpoints assessed 1 month post Dose 3 of TETRActHib:

- Anti-diphtheria antibody titers measured by ELISA
- Anti-tetanus antibody titers measured by ELISA
- Anti-BPT antibody titers measured by ELISA
- Anti-PRP antibody titers measured by ELISA

Proof-of-concept

- The time to first malaria infection (first recording of infection of asexual stage falciparum parasites detected by the active detection of infection surveillance) over a period starting 14 days after last vaccination with RTS,S/AS02D extending for 12 weeks.
- The asexual *P. falciparum* parasitemia (prevalence and density) at 3½ months post Dose 3 (Month 6).

Tertiary endpoints

Safety

- Occurrence of serious adverse events during the single-blind surveillance period (from Month 7 to Month 14 inclusive).

Immunogenicity

Endpoints assessed 12 months post Dose 3:

- Anti HBs antibody titers
- Anti-CS antibody titers.

**Exploratory Endpoints**	*From Day 0 and extending to 3½ months post Dose 3 of Engerix-B or RTS,S/AS02D*<br><br>• The time to the first clinical episode of symptomatic *P. falciparum* malaria (defined as the presence of *P. falciparum* asexual parasitemia above 500 per µL on Giemsa stained thick blood films AND the presence of fever [axillary temperature ≥ 37.5°C] at the time of presentation) detected by passive case detection.<br><br>• The time to the first clinical episode of symptomatic *P. falciparum* malaria (defined as the presence of *P. falciparum* asexual parasitemia above 500 per µL on Giemsa stained thick blood films AND the presence of fever [axillary temperature ≥ 37.5°C] at the time of presentation) detected by passive case detection and active case detection.<br><br>*From Day 0 and extending to 14 months post Dose 1 of Engerix-B or RTS,S/AS02D*<br><br>• The time to the first clinical episode of symptomatic *P. falciparum* malaria (defined as the presence of *P. falciparum* asexual parasitemia above 500 per µL on Giemsa stained thick blood films AND the presence of fever [axillary temperature ≥ 37.5°C] at the time of presentation) detected by passive case detection.<br><br>• The total number of clinical episodes of symptomatic *P. falciparum* malaria (defined as the presence of *P. falciparum* asexual parasitemia above 500 per µL on Giemsa stained thick blood films AND the presence of fever [axillary temperature ≥ 37.5°C] at the time of presentation) detected by passive case detection.<br><br>*Endpoint assessed at screening, 1 month post Dose 3 and 3½ months post Dose 3 of Engerix-B or RTS,S/AS02D*<br><br>• The frequency of CS-specific CD4+ and CD8+ T cells , as measured by intracellular cytokine staining.

TABLE OF CONTENTS

PAGE

SYNOPSIS **9**

LIST OF ABBREVIATIONS **22**

GLOSSARY OF TERMS **25**

1. INTRODUCTION **28**
1.1. Malaria 28
1.2. Hepatitis B 29
1.3. Rationale for vaccine development 29
1.3.1. AS02 Adjuvant 29
1.3.2. Rationale for the formulation of RTS,S/AS02D (0.5 mL dose) 30
1.3.3. The RTS,S/AS02 candidate malaria and hepatitis B vaccine; data collected to date 31
1.3.3.1. Adult trials 31
1.3.3.2. Phase I/II pediatric trials of RTS,S/AS02A 31
1.3.3.3. Phase IIb pediatric trial of RTS,S/AS02A (Malaria-026) 38
1.3.3.3.1. Primary Efficacy Endpoint, Malaria-026 39
1.3.3.3.2. Secondary Efficacy Endpoints of clinical malaria disease, Malaria-026 39
1.3.3.3.3. Incidence of moderate anemia, Malaria-026 40
1.3.3.3.4. All clinical episodes meeting Primary Case Definition for Malaria, Malaria-026 40
1.3.3.3.5. Exploratory Efficacy Endpoints of more severe disease, Malaria-026 41
1.3.3.3.6. Association of CS response with efficacy, Malaria-026 42
1.3.3.3.7. Efficacy analysis in the ITT Cohort from Day 0, Malaria-026 43
1.3.3.3.8. Efficacy against infection, Malaria-026 43
1.3.3.3.9. Immunogenicity, Malaria-026 43
1.3.3.4. Phase II pediatric trial of RTS,S/AS02D; Bridging Study (Malaria-034) 47
1.3.4. Concurrent protection against hepatitis B 50
1.4. Rationale for the study design 50
1.4.1. Safety monitoring plan 51
1.4.1.1. Safety data collection on TETRActHib 51
1.4.2. Assessment of proof of concept 51
~~1.5. Rationale for studying the determinants of vaccine response~~ 52
1.6. Rationale for testing cell-mediated immunity 53

2. OBJECTIVE(S) 54
2.1. Primary: Safety 54
2.2. Secondary: Immunogenicity..54
2.3. Secondary: Proof-of-concept..54

3. STUDY DESIGN OVERVIEW..55

4. STUDY COHORT & STUDY SITE...57
4.1. Study Site 57
4.1.1. Centro de Investigação em Saude de Manhiça 57
4.1.2. Study site; geography 58
4.1.3. Study site; malaria transmission...58
4.1.4. Study site; population..59
4.1.5. Study site; health care provision in the study area 60
4.1.5.1. Manhiça District Hospital 60
4.1.5.2. Ilha Josina Health Center...61
4.1.5.3. Taninga Health Center..61
4.1.5.4. Malaria control 61
4.1.5.5. EPI services...62
4.1.5.6. HIV services 62
4.1.5.6.1. HIV management; Adults 62
4.1.5.6.2. HIV management; Children.....................62
4.1.5.6.3. HIV management; Prevention of vertical transmission 62
4.2. Number of subjects 63
4.3. Inclusion criteria 63
4.4. Exclusion criteria for enrollment 63
4.5. Elimination criteria during the study..64
4.6. Contraindications to any vaccination dose 65
4.7. Contraindications to subsequent vaccination dose...................................65

5. CONDUCT OF STUDY..65
5.1. Ethics and regulatory considerations..65
5.1.1. Institutional Review Board/Independent Ethics Committee (IRB/IEC) 66
5.1.2. Informed consent 67
5.1.3. Data Safety Monitoring Board (DSMB)..69
5.1.3.1. Data Reviewed by the DSMB......................................70
5.1.4. Local Safety Monitor (LSM)..70
5.1.4.1. Data Reviewed by the LSM...71
5.1.5. Safety monitoring plan 71
5.1.6. Process for the suspension of vaccination 72
5.1.6.1. Indications for immediate suspension of all vaccination...72
5.1.6.2. Indications for suspension of enrolment to the trial...72
5.1.6.3. Process if the trial is suspended 72
5.1.7. Completion of immunization course for infants withdrawn during vaccination course 73
5.1.8. Rescue plan for Hepatitis B response 73
5.1.9. Rescue plan for D, T, Pw, and Hib responses..............................73
5.1.10. Recruitment 74

5.1.10.1. Community information 74
5.1.10.2. Antenatal visits 74
5.1.10.2.1. Management of infants born to HBsAg positive mothers 74
5.1.10.2.2. Management of HIV positive mothers and their infants 74
5.1.10.3. Screening of infants 75
5.1.11. Vaccination process 75
5.1.12. Home follow-up visits for assessment of reactogenicity (7-day follow-up period) 77
5.1.13. Monitoring of hematological and biochemical laboratory parameters 77
5.1.14. Surveillance for SAEs (all subjects) 78
5.1.14.1. At health facilities 78
5.1.14.2. In the community 79
5.1.15. Provision of health care 79
5.1.15.1. Management and treatment of malaria in all subjects 80
5.1.16. Passive case detection of clinical cases of malaria 80
5.1.17. Active detection of infection 81
5.2. Subject identification 82
5.3. Outline of study procedures 83
5.4. Detailed description of study stages/visits 85
5.5. Sample handling and analysis 93
5.5.1. Treatment and storage of biological samples 93
5.5.2. Laboratory assays 93
5.5.3. Serology plan 97

6. INVESTIGATIONAL PRODUCTS AND ADMINISTRATION 98
6.1. Study vaccines 98
6.1.1. RTS,S/AS02D candidate malaria and hepatitis B vaccine 98
6.1.2. TETRActHib 98
6.1.3. Engerix-B 99
6.2. Dosage and administration 99
6.2.1. RTS,S/AS02D (0.5 mL dose) 99
6.2.2. TETRActHib (0.5 mL dose) 99
6.2.3. Engerix-B (0.5 mL dose) 100
6.3. Storage 100
6.4. Treatment allocation and randomization 100
6.4.1. Randomization of supplies 100
6.4.2. Randomization of subjects 100
6.5. Method of blinding and breaking the study blind 100
6.6. Replacement of unusable vaccine doses 101
6.7. Packaging 102
6.8. Vaccine accountability 102
6.9. Concomitant medication/treatment 102

7. HEALTH ECONOMICS 103

8. ADVERSE EVENTS AND SERIOUS ADVERSE EVENTS 103
8.1. Definition of an adverse event 103
8.2. Definition of a serious adverse event 104

8.3. Clinical laboratory parameters and other abnormal assessments qualifying as adverse events and serious adverse events 105
8.4. Time period, frequency, and method of detecting adverse events and serious adverse events 105
8.4.1. Solicited adverse events 107
8.5. Evaluating adverse events and serious adverse events 107
8.5.1. Assessment of intensity 107
8.5.2. Assessment of causality 109
8.6. Follow-up of adverse events and serious adverse events and assessment of outcome 110
8.7. Prompt reporting of serious adverse events to GSK Biologicals 111
8.7.1. Time frames for submitting serious adverse event reports to GSK Biologicals 111
8.7.2. Completion and transmission of serious adverse event reports to GSK Biologicals 111
8.8. Regulatory reporting requirements for serious adverse events 113
8.9. Post study adverse events and serious adverse events 114
8.10. Pregnancy 114
8.11. Treatment of adverse events 115

9. SUBJECT COMPLETION AND WITHDRAWAL 115
9.1. Subject completion 115
9.2. Subject withdrawal 115
9.2.1. Subject withdrawal from the study 115
9.2.2. Subject withdrawal from investigational product 116

10. DATA EVALUATION: CRITERIA FOR EVALUATION OF OBJECTIVES 116
10.1. Primary Endpoints 116
10.1.1. Primary endpoint; safety 116
10.2. Secondary endpoints 116
10.2.1. Secondary endpoints for Safety 116
10.2.2. Secondary endpoints for Immunogenicity 117
10.2.3. Secondary endpoints for Proof of concept 117
10.3. Tertiary Endpoints 118
10.3.1. Tertiary endpoints for Safety 118
10.3.2. Tertiary endpoints for Immunogenicity 118
10.4. Exploratory Endpoints 118
10.5. Estimated sample size 119
10.5.1. Safety 119
10.5.2. Sample size for secondary endpoint immunology of Hepatitis B response 119
10.5.3. Sample size for secondary endpoint proof of concept 120
10.6. Study cohorts to be evaluated 120
10.6.1. Total Vaccinated Cohort 120
10.6.2. According to protocol (ATP) cohort for analysis of safety 120
10.6.3. According to protocol (ATP) cohort for analysis of immunogenicity 121
10.6.4. According to protocol (ATP) cohort for analysis of efficacy 121
10.7. Derived and transformed data 121
10.8. Planned Interim Analysis 122
10.8.1. Analysis of demographics/baseline characteristics 122
10.8.2. Analyses of safety 122

10.8.3. Analyses of immunogenicity 123
10.8.3.1. Assessment of non-inferiority Clinic Visit 10 (one month post Dose 3 of RTS,S/AS02D or Engerix-B)...123
10.8.3.2. Anti-HBs antibodies 123
10.8.3.3. Anti-CS antibodies 124
10.8.3.4. Anti-BPT antibodies 124
10.8.3.5. Anti-diphtheria toxoid antibodies 124
10.8.3.6. Anti-tetanus toxoid antibodies 124
10.8.3.7. Anti-PRP antibodies..124
10.9. Final Analyses...125
10.9.1. Analyses of safety 125
10.9.1.1. Primary endpoint..125
10.9.1.2. Other safety endpoints 125
10.9.2. Analyses of immunogenicity 125
10.9.2.1. Anti-CS antibodies 125
10.9.3. Analyses of efficacy 125
10.9.3.1. Calculation of the time at risk 125
10.9.3.2. Vaccine efficacy...126
10.9.3.3. Adjustment for covariates 127
10.9.4. Exploratory analysis 127
10.10. Annex analysis at study conclusion..128
10.10.1. Analyses of safety 128
10.10.2. Analyses of immunogenicity 128
10.10.2.1. Anti-HBs antibodies 128
10.10.2.2. Anti-CS antibodies 128
10.10.3. Analyses of efficacy 129
10.10.3.1. Exploratory analysis...129

11. ADMINISTRATIVE MATTERS 129

12. REFERENCES 129

APPENDICES

Appendix A World Medical Association Declaration of Helsinki 133

Appendix B Administrative Matters 137

Appendix C Overview of the Recruitment Plan 143

Appendix D Handling of Biological Samples Collected by the Investigator...............144

Appendix E Shipment of Biological Samples 147

Appendix F Laboratory Assays...149

Appendix G Vaccine supplies, packaging and accountability 151

Appendix H Amendments and Administrative Changes to the Protocol 153

List of Abbreviations

ADI	Active Detection of Infection
AE	Adverse event
ALT	Alanine aminotransferase
anti-CS	antibody to the *P. falciparum* circumsporozoite (CS) repeat protein
anti-HBsAg	antibody to the hepatitis B surface antigen
ATP	According to protocol
BCG	Bacillus Calmette-Guérin tuberculosis vaccine.
CI	Confidence interval
CISM	Centro de Investigação Em Saude de Manhiça, Mozambique.
CMI	Cell-mediated immunity
CRA	Clinical research associate
CRF	Case report form
CS	Circumsporozoite protein
CSC	Central study coordinator
CTL	Cytotoxic T-lymphocyte
cyr	Child years at risk
D	Diphtheria
DSMB	Data safety monitoring board
DTCOQ	Adsorbed diphtheria, tetanus and pertussis vaccine, licensed by Aventis Pasteur, Lyon
DTPw/Hib	Diphtheria, tetanus, whole-cell pertussis and *Hemophilus influenzae* type b vaccine
EIA	Enzyme immunosorbent assay
EIR	Entomological inoculation rate
EISR	Expedited investigator safety report
ELISA	Enzyme linked immunosorbent assay

EPI	Expanded program on immunization
FDA	Food and Drug Administration, United States
GCP	Good clinical practice
GMT	Geometric mean titer
GSK	GlaxoSmithKline
HBsAg	Hepatitis B surface antigen
HBV	Hepatitis B virus
Hep B	Hepatitis B
Hib	*Hemophilus influenzae* type B
HIV	Human immunodeficiency virus
IB	Investigator's brochure
ICF	Informed consent form
IEC	Independent ethics committee
IFN-γ	Interferon gamma
IM	Intramuscular
IND	Investigational new drug
IRB	Institutional review board
IU	International unit
kg	Kilogram
LSM	Local safety monitor
MedDRA	Medical Dictionary for Regulatory Activities
mg	Milligram
mL	Milliliter
MPL®	3-deacylated monophosphoryl lipid A
MVI	Malaria Vaccine Initiative
P. falciparum	*Plasmodium falciparum*

PATH	Program for Appropriate Technology in Health
PBS	Phosphate buffered saline
PCR	Polymerase chain reaction
PFS	Pre-filled syringe
PRP	The polyribosyl ribitol phosphate capsule of the *Hemophilus influenzae* bacterium
Pw	Whole-cell pertussis
QS 21	‘*Quillaja saponaria* 21’: a triterpene glycoside purified from the bark of *Quillaja saponaria*
RAP	Report and Analysis Plan
RF1	A protective epitope against HBsAg
RTS	Hybrid protein comprising S (hepatitis B surface antigen) and CSP portions
RTS,S	Particulate antigen, containing both RTS and S (hepatitis B surface antigen) proteins
SAE	Serious adverse event
SOP	Standard operating procedure
SP	sulfadoxine-pyrimethamine
T	Tetanus
WRAIR	Walter Reed Army Institute of Research

Glossary of Terms

Adverse event:	Any untoward medical occurrence in a patient or clinical investigation subject, temporally associated with the use of a medicinal product, whether or not considered related to the medicinal product.<br><br>An AE can therefore be any unfavorable and unintended sign (including an abnormal laboratory finding), symptom, or disease (new or exacerbated) temporally associated with the use of a medicinal product. For marketed medicinal products, this also includes failure to produce expected benefits (i.e. lack of efficacy), abuse or misuse.
Active detection of Infection (ADI):	The detection of malaria parasitemia on a blood sample taken at predefined time points, i.e. regardless of whether a child has signs or symptoms of infection.
Blinding:	A procedure in which one or more parties to the trial are kept unaware of the treatment assignment in order to reduce the risk of biased study outcomes. In a single-blind trial, the investigator and/or his staff are aware of the treatment assignment but the subject is not. In an observer-blind study, the subject and the study personnel involved in the clinical evaluation of the subjects are blinded while other study personnel may be aware of the treatment allocation. When the investigator and sponsor staff who are involved in the treatment or clinical evaluation of the subjects and review/analysis of data are also unaware of the treatment assignments, the study is double blind. Partially blind is to be used for study designs with different blinding levels between different groups, e.g. double blinded consistency lots which are open with respect to the control group. The level of blinding is maintained throughout the conduct of the trial, and only when the data are cleaned to an acceptable level of quality will appropriate personnel be unblinded or when required in case of a serious adverse event.
Central Study Coordinator:	An individual assigned by and centrally located at GSK Biologicals at Rixensart who is responsible for assuring proper conduct of a clinical study.
Data Safety Monitoring Board (DSMB):	The DSMB is an independent committee appointed to oversee ethical and safety aspects of the conduct of the study. See Section 5.1.3 for a full overview of the role and structure of the DSMB.
Eligible:	Qualified for enrollment into the study based upon strict adherence to inclusion/exclusion criteria.
eTrack	GSK’s clinical trials tracking tool

Evaluable:	Meeting all eligibility criteria, complying with the procedures defined in the protocol, and, therefore, included in the according-to-protocol (ATP) analysis (see Sections 4.3 and 4.4 for details on criteria for evaluability).
Investigational product:	A pharmaceutical form of an active ingredient or placebo being tested or used as a reference in a clinical trial, including a product with a marketing authorization when used in a way different from the approved form, or when used for an unapproved indication, or when used to gain further information about an approved use. The investigational products for this study are RTS,S/AS02D, Engerix-B and TETRActHib.
Local Safety Monitor (LSM):	The overall role of the Local Safety Monitor, an experienced physician based in-country, will be to support the study investigators and to act as a link between the investigators and the Data Safety Monitoring Board (DSMB) (see Section 5.1.4 for further details).
Medical Monitor:	An individual medically qualified to assume the responsibilities of the sponsor (GSK Biologicals) especially in regards to the ethics, clinical safety of a study and the assessment of adverse events.
Protocol amendment:	ICH defines a protocol amendment as: “A written description of a change(s) to or formal clarification of a protocol.” GSK Biologicals further details this to include a change to an approved protocol that affects the safety of subjects, scope of the investigation, study design, or scientific integrity of the study.
Protocol administrative change:	A protocol administrative change addresses changes to only logistical or administrative aspects of the study.<br><br>N.B. Any change that falls under the definition of a protocol amendment (e.g. a change that affects the safety of subjects, scope of the investigation, study design, or scientific integrity of the study) MUST be prepared as an amendment to the protocol.
Randomization:	Process of random attribution of treatment to subjects in order to reduce bias of selection
Solicited adverse event:	Adverse events (AEs) to be recorded as endpoints in the clinical study. The presence/occurrence/intensity of these events is actively solicited from the subject or an observer during a specified post-vaccination follow-up period.

Study Monitor:	An individual assigned by the sponsor who is responsible for assuring proper conduct of a clinical study.
Subject:	Term used throughout the protocol to denote an individual that has been contacted in order to participate in the clinical study, either as a recipient of the investigational product(s) or as a control.
Treatment:	Term used throughout the clinical study to denote a set of investigational product(s) or marketed product(s) or placebo intended to be administered to a subject, identified by a unique number, according to the study randomization or treatment allocation.
Treatment number:	A unique number identifying a treatment to a subject, according to the study randomization or treatment allocation.
Unsolicited adverse event:	Any adverse event (AE) reported in addition to those solicited during the clinical study. Also any “solicited” symptom with onset outside the specified period of follow-up for solicited symptoms will be reported as an unsolicited adverse event.

1. INTRODUCTION

The RTS,S/AS02 candidate malaria and hepatitis B vaccine consists of sequences of the circumsporozoite (CS) protein and hepatitis B surface antigen (HBsAg) adjuvanted with AS02 (proprietary oil in water emulsion, MPL® and QS21 immunostimulants). The vaccine is being developed for the routine immunization of infants and children living in malaria endemic areas. This vaccine would offer protection against malaria disease due to the parasite *Plasmodium falciparum*. The vaccine would also provide protection against infection with hepatitis B virus (HBV).

This study is being initiated following the conclusive demonstration of efficacy in the trial of RTS,S/AS02A in children aged 1 to 4 years in Mozambique (Malaria-026: see Section 1.3.3.3 below). The vaccine demonstrated 30% (95% CI 11 to 45) protection against clinical malaria disease and 58% (95% CI 16 to 81) against severe malaria disease. The vaccine also demonstrated 45% (95% CI 31 to 56) delay in the time to first infection with *P. falciparum* parasites.

RTS,S/AS02D (0.5 mL dose) is composed of the same active constituents in the same quantities as in a 0.25 mL dose of RTS,S/AS02A. A bridging study was conducted comparing RTS,S/AS02D to RTS,S/AS02A. Non-inferiority of the anti-CS and anti-HBs immune response was demonstrated. The safety profile of RTS,S/AS02D was comparable to that of RTS,S/AS02A (see Section 1.3.2 for the rationale behind the development of the RTS,S/AS02D vaccine and Section 1.3.3.4 for a summary of the results of the Malaria-034 trial.

RTS,S/AS02D is being developed to be integrated into the Expanded Program on Immunization (EPI) in malaria endemic regions. This trial is the first administration of RTS,S/AS02D in infants. The primary objective of this study is to assess vaccine safety in young infants. It will also provide key development information on the comparison of the hepatitis B response induced by RTS,S/AS02D compared to a licensed hepatitis B vaccine, and proof-of-concept of efficacy against infection. This product development plan is conducted under a partnership agreement with PATH's Malaria Vaccine Initiative (MVI) and is guided by a joint MVI/GSK Steering Committee.

1.1. Malaria

Four species of the *Plasmodium* protozoan parasite are the etiologic agents of malaria in humans (*P. falciparum, P. vivax, P. ovale* and *P. malariae*). Of these four parasites, *P. falciparum* is the major cause of severe morbidity and mortality.

There can be no doubt of the importance of *P. falciparum* malaria as a major cause of human suffering and economic drain across sub-Saharan Africa [Breman 2001a, Gallup 2001]. In this region, it causes the deaths of between 0.5 and 2.0 million children every year and is the most common reason for admission to hospital, leading each year to about 300 million clinical episodes in children under five years [Breman 2001a].

The incidence of malaria in much of Africa is increasing for a variety of reasons: changes in agricultural practices, armed conflicts, migration of refugees, increasing drug

resistance to conventional anti-malarial drugs, and insecticide resistance of the anopheline mosquito vectors. It is estimated that the number of cases of clinical malaria will more than double over the next 20 years without effective control. The burden of malaria at the country level correlates closely with the rate of economic development even after adjustment for confounding factors, indicating that malaria is an important constraint on economic progress [Breman 2001b].

1.2. Hepatitis B

Hepatitis B is an infection of the liver due to hepatitis B virus (HBV); it is an important public health problem across the developing world. World-wide approximately 350 million people carry HBV and about 1 million chronic carriers die annually [Vryheid 2001]. In sub-Saharan Africa infection primarily occurs by one of three routes: perinatally from mother to infant, during childhood through close personal contact and from adolescence onwards through sexual contact. The likelihood of the infection becoming chronic is dependent upon the age at infection: 90% if infected in infancy, 30% to 50% if infected between the ages of 1 to 4 years, and low in adulthood. For those that become chronically infected during childhood the risk of death from HBV-related liver cancer or cirrhosis in adult life is approximately 25% [World Health Organization 2003].

1.3. Rationale for vaccine development

The RTS,S/AS02 vaccine is hypothesized to reduce frequency and severity of clinical disease episodes through its impact on the initial sporozoite and liver stage parasite burden, leading to a significant reduction of the infectious inoculum of liver stage merozoites released into the blood stream. This hypothesis is fully consistent with the data observed in clinical trials to date. In the homologous sporozoite challenge model the vaccine prevents approximately 40% of individuals from becoming infected and a non-protected delay in the prepatent period is observed. [Kester, 2001]. In the field efficacy study in children (Malaria-026; refer to Section 1.3.3.3) protection against severe malaria disease tended to be higher than protection against all clinical disease although confidence intervals were widely overlapping; 58% (95% CI 16% to 81%) compared to 30% (95% CI 11% to 45%). A vaccine that induces partial immunity against pre-erythrocytic stages of disease may provide protection to vulnerable young children from the severe forms of the disease, whilst continuing exposure allows them to build up natural immunity. Acquisition of natural immunity may be important to prevent a shift of severe disease burden to older age groups upon waning of vaccine-induced immunity.

1.3.1. AS02 Adjuvant

The vaccine antigen, RTS,S is formulated in the GSK proprietary adjuvant system 2 (AS02); containing a proprietary oil-in-water emulsion and the immunostimulants QS21 (a triterpene glycoside purified from the bark of *Quillaja saponaria*) and MPL®. The choice of AS02 as the formulation for a malaria pre-erythrocytic vaccine was based on extensive pre-clinical experimentation conducted by GSK Biologicals and collaborators. Pre-clinical immunogenicity studies in mice and in rhesus monkeys demonstrated conclusively that this formulation is a powerful inducer of both humoral and CMI

responses, including IFN-γ-secreting T-helper 1 lymphocytes [GSK data on file, Malaria Investigator's Brochure 2003].

A confirmation of the critical importance of adequate formulation and of the value of AS02 as an adjuvant system for pre-erythrocytic malaria vaccine was obtained in a clinical trial conducted in collaboration with the Walter Reed Army Institute of Research (WRAIR), WRMAL-003 [Stoute 1997]. In this Phase I/IIa trial, a vaccine consisting of the RTS,S antigen formulated in AS02 protected six out of seven volunteers (86% vaccine efficacy; (95% CI 0.02 to 0.88; $p < 0.005$) from infection following a sporozoite challenge delivered by the bites of laboratory-reared infectious *Anopheles* mosquitoes. In this trial, two other vaccine formulations (adjuvanted with AS04 and AS03) of the same RTS,S antigen were also evaluated but afforded only marginal protection against challenge.

The AS02 formulation to be used in malaria vaccine trials no longer contains any thiomersal as preservative and is supplied as monodose pre-filled syringes.

1.3.2. Rationale for the formulation of RTS,S/AS02D (0.5 mL dose)

The vaccine under investigation in this study, RTS,S/AS02D (0.5 mL dose) is a diluted formulation of RTS,S/AS02A (0.25 mL dose), which was the vaccine used in the recent proof-of-concept study (Malaria-026). This new formulation was necessary to make the vaccine presentation compatible with the established delivery devices of the Expanded Program of Immunization (EPI) of the World Health Organization (WHO). Currently all intramuscular vaccines in the EPI schedule are administered at a dose volume of 0.5 mL. A recent advance has been the introduction of auto-disable syringes (to prevent reuse and the possible spread of blood-borne infection) with a standard volume of 0.5 mL. RTS,S/AS02D (0.5 mL dose) is composed of the same active constituents in the same quantities as in a 0.25 mL dose of RTS,S/AS02A. However the final volume of the vaccine is 0.5 mL. Some of the formulations of RTS,S/AS02 are detailed in Table 1.

Prior to the initiation of this trial, a bridging study of RTS,S/AS02D to RTS,S/AS02A (0.25 mL dose) was conducted in Mozambican children aged 3 to 5 years [GSK Data on file, Malaria-034 2003]. This study demonstrated that the CS and HBs responses of RTS,S/AS02D (0.5 mL dose) were non-inferior to those of RTS,S/AS02A (0.25 mL dose) and that RTS,S/AS02D is safe and has an acceptable reactogenicity profile. A summary of the results of this trial can be found in Section 1.3.3.4.

Table 1 Formulations of RTS,S/AS02

Formulation	RTS,S (µg)	MPL® (µg)	QS21 (µg)	Volume (mL)	Comments
RTS,S/AS02A (0.5 mL dose)	50	50	50	0.5	Efficacy demonstrated in adults in an endemic country (Malaria-005 [a])
RTS,S/AS02A (0.25 mL dose)	25	25	25	0.25	Efficacy demonstrated in children in an endemic area (Malaria-026 [b])
RTS,S/AS02D (0.5 mL dose)	25	25	25	0.5	Bridged to RTS,S/AS02A (0.25 mL dose) (Malaria-034) [c]

a GSK data on file, Malaria-005 Clinical Study Protocol, 1997
b GSK data on file, Malaria-026 Clinical Study Protocol, 2003
c GSK data on file, Malaria-034 Clinical Study Protocol, 2003

1.3.3. The RTS,S/AS02 candidate malaria and hepatitis B vaccine; data collected to date

1.3.3.1. Adult trials

GlaxoSmithKline (GSK) Biologicals is developing a candidate vaccine against *P. falciparum* malaria, the RTS,S/AS02A malaria vaccine, which has been shown to be safe and immunogenic in malaria-experienced adult populations in The Gambia and Kenya and malaria-naïve adult populations in Belgium and the USA [Stoute 1997, Doherty 1999, Bojang 2001].

The vaccine has been shown to protect between 42% and 86% of healthy non-immune volunteers against infection in homologous sporozoite challenge studies, when given according to a 2 or 3 dose vaccination schedule [GSK data on file, Malaria Investigator’s Brochure 2003, Kester 2001]. In addition a prolongation of the pre-patent period was observed in the majority of non-protected volunteers [Kester 2001].

In a double-blind, randomized, controlled Phase IIb study in an endemic region of The Gambia, 306 men were enrolled of which 250 men received 3 doses of the vaccine (n = 131) or a control (rabies vaccine, n = 119). The vaccine was safe and well tolerated, and efficacy against infection was 34% (95% CI: 8% to 53%; p = 0.014) over one malaria transmission season. Protection was high initially but waned over time; during the first 9 weeks of surveillance efficacy was 71% (95% CI: 46% to 85%; p < 0.0005), decreasing to 0% (95% CI: -52% to 34%) over the subsequent 6 weeks [Bojang 2001]. A single booster dose was given prior to the next malaria season. The efficacy against infection measured was 47% (95% CI 4% to 71%; p = 0.037).

1.3.3.2. Phase I/II pediatric trials of RTS,S/AS02A

Following demonstration of the safety, immunogenicity and efficacy of the RTS,S/AS02A vaccine in adults, the candidate vaccine progressed to clinical evaluation in children. Two Phase I safety and immunogenicity studies evaluating 0.1 mL, 0.25 mL

and 0.5 mL doses of RTS,S/AS02A have been conducted in Gambian children aged 1 to 11 years (Malaria-015 [GSK data on file] and Malaria-020 [GSK data on file])—a 0.5 mL dose of RTS,S/AS02A contains 50 µg RTS,S, 50 µg MPL®, 50 µg QS21 and oil-in-water emulsion. Each study included a control arm vaccinated with Imovax® human diploid cell rabies vaccine (Aventis Pasteur, Lyon).

These studies were to aid in the selection of the dose level to be used in the future pediatric development of the RTS,S/AS02 candidate vaccine.

The solicited symptoms are displayed in Table 2 and Table 3 (corresponding to Malaria-015 and Malaria-020 respectively). Pain at the injection site was a frequent symptom across all study groups. The incidence of swelling increased with dose level. The majority of general solicited symptoms were mild to moderate in intensity and short lasting. In children aged 6 to 11 years headache was the most frequently occurring general solicited symptom in subjects receiving RTS,S/AS02A (any dose level). In the younger children (aged 1 to 5 years), the most frequently occurring general symptom in subjects receiving 0.1 mL dose RTS,S/AS02A was fever, reported after 11% of doses compared to 26% of doses in the rabies control group. In subjects receiving 0.25 mL dose RTS,S/AS02A, loss of appetite was the most frequent symptom, being reported after 12% of doses, which compared to 20% of doses in the control group. In subjects receiving 0.5 mL dose RTS,S/AS02A, irritability/fussiness was most frequently reported, occurring after 27% of doses compared to 12% of doses in the control group. Grade 3 general solicited symptoms were infrequent in both studies and resolved or decreased in intensity within 24 hours.

Unsolicited symptoms were recorded with similar (6 to 11 year-olds) or lower frequency (1 to 5 year-olds) in the study vaccine groups compared to the control vaccine groups. The majority of unsolicited symptoms were mild to moderate in intensity and unrelated to vaccination.

Hematocrit values were generally low but comparable between study groups. Six children in the RTS,S/AS02A groups (one in 0.1 mL dose group, five in 0.25 mL dose group) experienced moderate anemia, defined as a hematocrit of 15% to 24% during the period to 30 days post Dose 3. There were no cases of anemia in the 0.5 mL or control groups. All these children had documented malaria episodes before or at the time that anemia was recorded.

All children were monitored for liver function. Among the 6 to 11 year-olds, two children in the RTS,S/AS02A groups experienced a transient rise in ALT levels judged not to be related to vaccination and not clinically relevant. In the younger children, increases in ALT levels from pre-vaccination were observed in 2 subjects in RTS,S/AS02A groups and 2 subjects in the rabies groups. Of these, only one case in the rabies control group was judged to be clinically relevant and vaccination was discontinued after Dose 1. No other clinically relevant abnormalities of hematological or biochemical laboratory parameters were observed.

In the interval to 30 days post Dose 3, 6 serious adverse events (SAEs) in total occurred. One SAE occurred in the 6 to 11 year old study; a case of bronchopneumonia was

reported in a subject in the rabies-control group. The event was not considered causally related to vaccination and the subject made a full recovery. Five SAEs were reported in the 1 to 5 year old study. Among the RTS,S/AS02A recipients one subject suffered acute malaria with acute upper respiratory tract infection (0.1 mL dose) and one subject suffered cerebral malaria (0.25 mL dose). In the rabies control group three SAEs were reported: acute severe malaria with urinary tract infection and salmonella septicemia, bronchopneumonia with bronchial asthma and accidental death due to drowning. All SAEs were considered not to be related to study vaccines. Apart from the fatal SAE, all subjects made a full recovery and were not withdrawn from the study.

A total of 20 subjects out of 135 subjects enrolled in the Malaria-015 study were under 2 years of age. The safety and reactogenicity profiles of these subjects receiving the RTS,S/AS02A candidate vaccine were comparable to that seen in the older children.

Table 4 details the antibody responses to anti-CS and Table 5 the antibody responses to anti-HBs for these two studies. All dose levels of RTS,S/AS02A (0.1 mL, 0.25 mL and 0.5 mL doses) were highly immunogenic for anti-CS and anti-HBs antibodies, irrespective of pre-vaccination HBsAg serostatus.

For both Malaria-015 and Malaria-020, all subjects receiving RTS,S/AS02A were seropositive for anti-CS antibodies post Dose 2. Post Dose 3 the lowest anti-CS antibody geometric mean titers (GMTs) occurred in the 0.1 mL RTS,S/AS02A dose group (Table 4 below). In the 0.25 mL and 0.5 mL RTS,S/AS02A dose groups, similar GMT values were recorded. All subjects in the RTS,S/AS02A groups were seroprotected for anti-HBs antibodies post Dose 2 in Malaria-015 and post Dose 3 (first post-vaccination assessment) in Malaria-020; the highest GMTs were observed in the 0.25 mL RTS,S/AS02A dose group in Malaria-015 and in the 0.5 mL RTS,S/AS02A dose group in Malaria-020 (Table 5 below). Overall, the GMT values observed in this population of children aged 1 to 11 years were within the ranges seen in previous studies with the RTS,S/AS02A vaccine in malaria-naïve adult subjects [Kester 2001].

Table 2 Frequency of solicited symptoms per dose during the 4-day follow-up period for each vaccine group (Total Cohort, Malaria-015)

		RTS,S/AS02A (0.1 mL) N=60		Imovax N=30		RTS,S/AS02A (0.25 mL) N=59		Imovax N=30		RTS,S/AS02A (0.5 mL) N=60		Imovax N=30	
Symptom		n	%	n	%	n	%	n	%	n	%	n	%
Injection site Pain	Any	53	88.3	21	70.0	53	89.8	27	90.0	59	98.3	26	86.7
	Grade 3	0	0.0	0	0.0	4	6.8	0	0.0	8	13.3	0	0.0
Injection site Swelling	Any	5	8.3	1	3.3	10	16.9	0	0.0	12	20.0	0	0.0
	Grade 3	1	1.7	0	0.0	1	1.7	0	0.0	4	6.7	0	0.0
Limited arm Motion	Any	5	8.3	1	3.3	12	20.3	2	6.7	11	18.3	0	0.0
	Grade 3	0	0.0	0	0.0	2	3.4	0	0.0	2	3.3	0	0.0
Fever*	Any*	6	10.0	0	0.0	5	8.5	3	10.0	6	10.0	2	6.7
	Grade 3	1	1.7	0	0.0	0	0.0	0	0.0	0	0.0	0	0.0
	Grade 3/Rel	0	0.0	0	0.0	0	0.0	0	0.0	0	0.0	0	0.0
Malaise	Any	5	8.3	0	0.0	8	13.6	2	6.7	11	18.3	2	6.7
	Grade 3	0	0.0	0	0.0	0	0.0	0	0.0	1	1.7	0	0.0
	Grade 3/Rel	0	0.0	0	0.0	0	0.0	0	0.0	1	1.7	0	0.0
Nausea	Any	4	6.7	1	3.3	2	3.4	1	3.3	6	10.0	0	0.0
	Grade 3	0	0.0	0	0.0	0	0.0	0	0.0	0	0.0	0	0.0
	Grade 3/Rel	0	0.0	0	0.0	0	0.0	0	0.0	0	0.0	0	0.0
Headache	Any	11	18.3	4	13.3	14	23.7	4	13.3	12	20.0	3	10.0
	Grade 3	0	0.0	0	0.0	0	0.0	0	0.0	2	3.3	0	0.0
	Grade 3/Rel	0	0.0	0	0.0	0	0.0	0	0.0	1	1.7	0	0.0

N = number of documented doses.
n/% = number/percentage of doses followed by a local/general symptom.
*axillary body temperature ≥ 37.5°C.
Grade 3 limited arm motion; abduction at the shoulder < 30°.
Grade 3 injection site pain; spontaneously painful.
Grade 3 swelling; > 50 mm and persisting for more than 24 hours.
Grade 3 fever; axillary body temperature ≥ 39.0°C.
For other symptoms; adverse event that prevents normal activity.
Rel = related to study vaccine.
Note: all local injection site symptoms were considered related to study vaccine.

Table 3 Frequency of solicited symptoms per dose during the 4-day follow-up period for each vaccine group (Total Cohort, Malaria-020)

		RTS,S/AS02A (0.1 mL) N=89		Imovax N=39		RTS,S/AS02A (0.25 mL) N=89		Imovax N=45		RTS,S/AS02A (0.5 mL) N=89		Imovax N=41	
Symptom		n	%	n	%	n	%	n	%	n	%	n	%
Injection site Pain	Any	70	78.7	34	87.2	75	84.3	38	84.4	82	92.1	32	78.0
	Grade 3	1	1.1	0	0.0	5	5.6	1	2.2	13	14.6	0	0.0
Injection site Swelling	Any	5	5.6	2	5.1	23	25.8	2	4.4	26	29.2	3	7.3
	Grade 3	5	5.6	1	2.6	16	18.0	2	4.4	24	27.0	1	2.4
Fever*	Any*	10	11.2	10	25.6	8	9.0	9	20.0	15	16.9	5	12.2
	Grade 3	1	1.1	1	2.6	1	1.1	1	2.2	0	0.0	1	2.4
	Grade 3/Rel	0	0.0	1	2.6	1	1.1	1	2.2	0	0.0	1	2.4
Drowsiness	Any	8	9.0	2	5.1	7	7.9	4	8.9	5	5.6	2	4.9
	Grade 3	1	1.1	0	0.0	0	0.0	1	2.2	0	0.0	0	0.0
	Grade 3/Rel	0	0.0	0	0.0	0	0.0	0	0.0	0	0.0	0	0.0
Loss of Appetite	Any	7	7.9	1	2.6	11	12.4	9	20.0	13	14.6	4	9.8
	Grade 3	1	1.1	0	0.0	2	2.2	2	4.4	2	2.2	0	0.0
	Grade 3/Rel	0	0.0	0	0.0	2	2.2	1	2.2	2	2.2	0	0.0
Irritability/ Fussiness	Any	4	4.5	0	0.0	9	10.1	6	13.3	24	27.0	5	12.2
	Grade 3	0	0.0	0	0.0	0	0.0	1	2.2	3	3.4	0	0.0
	Grade 3/Rel	0	0.0	0	0.0	0	0.0	1	2.2	3	3.4	0	0.0

N = number of documented doses.
n/% = number/percentage of doses followed by a local/general symptom.
*axillary body temperature ≥ 37.5°C.
Grade 3 injection site pain; cries when limb is moved/spontaneously painful.
Grade 3 swelling; > 20 mm.
Grade 3 fever; axillary body temperature ≥ 39.0°C.
Grade 3 drowsiness; drowsiness that prevents normal activity.
Grade 3 loss of appetite; not eating at all.
Grade 3 irritability/fussiness; crying that cannot be comforted/prevents normal activity.
Rel = related to study vaccine.
Note: all local injection site symptoms were considered related to study vaccine.

Table 4 Geometric Mean Titers (GMT) for anti-CS antibody titers (ATP Cohort for Immunogenicity for each study) post Dose 3

	RTS,S/AS02 dose volume			RTS,S/AS02D	Imovax (95% CI)	Engerix-B (95% CI)	Prevnar & Hiberix
Study	0.1 mL (95% CI)	0.25 mL (95% CI)	0.5 mL (95% CI)				
Malaria-015 [a]	30.5 (18.4—50.4)	73.7 (42.4 —128.0)	62.3 (38.7 —100.5)	--	1.0 (0.4—1.0)	--	--
Malaria-020 [a]	69.6 (49.8—97.2)	90.3 (60.7 —134.4)	84.1 (56.4 —125.4)	--	0.8 (0.7—1.0)	--	--
Malaria-025 [a]	--	270.4 (182.7—400.3)	--	--	--	0.6 (0.5—0.7)	--
Malaria-026 [b] (subj. < 24 m)	--	273.9 (228.7—328.1)	--	--	--	--	0.3 (0.3—0.3)
Malaria-026 [b] (subj. ≥ 24 m)	--	158.1 (141.9—176.2)	--	--		0.3 (0.3—0.4)	--
Malaria-034 [b]	--	179.6 (145.9—221.0)	--	190.9 (150.3—242.4)	--	--	--

[a]: GMTs for anti-CS antibodies were measured at WRAIR in µg/mL for these studies
[b]: GMTs for anti-CS antibodies were measured at GSK Biologicals in EL.U/mL for these studies

Table 5 Geometric Mean Titers (GMT) for anti-HBs antibody titers (ATP Cohort for Immunogenicity for each study) post Dose 3

	RTS,S/AS02 dose volume			RTS,S/AS02D	Imovax (95% CI)	Engerix-B (95% CI)	Prevnar & Hiberix
Study	0.1 mL (95% CI)	0.25 mL (95% CI)	0.5 mL (95% CI)				
Malaria-015	16442 (9204—29372)	32374 (14418—72694)	16604 (8627—31955)	--	27 (9—74)	--	--
Malaria-020	34925 (18482—65999)	54711 (27784—107737)	68041 (39221—118036)	--	87 (31—239)	--	--
Malaria-025	--	10386 (4872—22142)	--	--	--	328 (108—99631)	--
Malaria-026 (subj. < 24 m)	--	51035 (27918—93291)	--	--	--	--	67 (33—135)
Malaria-026 (subj. ≥ 24 m)	--	11368 (8518—15171)	--	--		349 (236—517)	--
Malaria-034	--	23977 (17895—32126)	--	17410 (13322—22751)	--	--	--

The 0.25 mL dose RTS,S/AS02A was selected for future pediatric development, because it exhibited comparable immunogenicity to the 0.5 mL dose, with a tendency to a lower reactogenicity profile.

Subsequent to these studies, RTS,S/AS02A (0.25 mL dose) was evaluated in a double-blind, randomized, controlled Phase I study under a new schedule (0, 1, 2 month) in children aged 1 to 4 years in Mozambique (Malaria-025 [GSK data on file]). Subjects received RTS,S/AS02A (0.25 mL dose) or Engerix-B hepatitis B vaccine (GSK Biologicals, Rixensart, Belgium). The frequency of solicited symptoms per dose during the 4-day follow-up period for each vaccine group during the study is summarized in Table 6.

As previously observed in Malaria-020, local reactions at the site of injection were common. Unlike Malaria-020 where swelling at the injection site was the most frequently observed local reaction, pain was the most frequently observed reaction in Malaria-025. Grade 3 swelling was reported after 23% of doses of RTS,S/AS02A (0.25 mL dose), but did not occur following administration of Engerix-B. The incidence of swelling in the RTS,S/AS02A group decreased after Dose 2 of vaccine, but increased after Dose 3.

Few solicited general adverse events were reported. In the RTS,S/AS02A group the most frequently reported solicited adverse events related to vaccination were fever (9.5% after all doses) followed by loss of appetite (7.5% after all doses). In the Engerix-B comparator group, fever (1.2%) was the only solicited general adverse event related to vaccination. There was no trend in increase in solicited general adverse events related to vaccination with subsequent doses. Only one Grade 3 solicited general adverse event was reported in the study; one subject suffered fever in the RTS,S/AS02A group. All solicited general symptoms reported resolved within the 4 day follow-up period after vaccination. The frequency of solicited symptoms was similar to that previously observed in Malaria-020.

Unsolicited adverse events were recorded over a 30-day follow-up period after vaccination. Unsolicited symptoms were recorded with similar frequency in the study vaccine group compared to the control vaccine group. No unsolicited event was considered by the investigator to be related to vaccination. The unsolicited events

reflected the pattern of childhood morbidity expected in the population and unsolicited adverse events were balanced between treatment groups in terms of frequency and severity.

Table 6 Frequency of solicited symptoms per dose during the 4-day follow-up period for each vaccine group (Total Cohort, Malaria-025)

		RTS,S/AS02A (0.25 mL dose) N=84		Engerix-B N=83	
Symptom		n	%	n	%
Injection site pain	Any	25	29.8	3	3.6
	Grade 3	1	1.2	0	0.0
Injection site swelling	Any	63	75.0	37	44.6
	Grade 3	19	22.6	0	0.0
Fever	Any*	12	14.3	3	3.6
	Grade 3	0	0.0	1	1.2
	Grade 3/Rel	1	1.2	0	0.0
Drowsiness	Any	4	4.8	1	1.2
	Grade 3	0	0.0	0	0.0
	Grade 3/Rel	0	0.0	0	0.0
Loss of Appetite	Any	10	11.9	1	1.2
	Grade 3	0	0.0	0	0.0
	Grade 3/Rel	0	0.0	0	0.0
Irritability	Any	6	7.1	2	2.4
	Grade 3	0	0.0	0	0.0
	Grade 3/Rel	0	0.0	0	0.0

N = number of documented doses.
n/% = number/percentage of doses followed by a local/general symptom.
*axillary body temperature ≥ 37.5°C.
Grade 3 injection site pain; cries when limb is moved/spontaneously painful.
Grade 3 swelling; > 20 mm.
Grade 3 fever; axillary body temperature ≥ 39.0°C.
Grade 3 drowsiness; drowsiness that prevents normal activity.
Grade 3 loss of appetite; not eating at all.
Grade 3 irritability/fussiness; crying that cannot be comforted/prevents normal activity.
Rel = related to study vaccine.
Note: all local injection site symptoms were considered related to study vaccine.

Two children experienced moderate anemia (Hematocrit 15 to 24%) during the period to one month post Dose 3. One child who received RTS,S/AS02A had anemia associated with an acute case of malaria. In the control group the cause was not identified but the child recovered when administered a course of antibiotics and iron supplementation.

In total 7 subjects in the control group and 3 subjects in the RTS,S/AS02A group had an elevation in ALT during the period to one months post Dose 3 (reference range < 60 IU/L). One subject in the control group was observed to have an ALT level of 61 at a single time point. The subject was clinically well and, because the value was just outside the reference range, he was not investigated further. In the other 9 subjects the raised ALT was associated with a viral hepatitis. Five of these subjects had acute hepatitis A. One subject in each group was a chronic carrier of hepatitis B and 1 subject in the RTS,S/AS02A group was suffering from acute hepatitis B. One other subject who received Engerix-B was HBsAg positive and was a probable chronic carrier of hepatitis B; no confirmatory serology was performed.

No other clinically relevant abnormalities of hematological or biochemical laboratory parameters were observed.

A total of four serious adverse events were reported, two in each group, during the period to one month post Dose 3. One subject in the Engerix-B group suffered *P. falciparum* malaria; a second subject suffered glomerulonephritis secondary to skin lesions. In the RTS,S/AS02A (0.25 mL dose) group, one subject suffered a febrile convulsion 15 days after the second vaccination and another subject suffered from bronchopneumonia. All SAEs were considered not to be related to study vaccines. All subjects made a full recovery and were not withdrawn from the study.

All subjects receiving RTS,S/AS02A (0.25 mL dose) were seropositive (titers $\geq$ 1 µg/mL) for anti-CS antibodies 14 days after Dose 1 and titers increased with each subsequent injection. Post Dose 3 a GMC of 270.4 µg/mL (95% CI 183 to 400 µg/mL) was observed in the group receiving RTS,S/AS02A (0.25 mL dose), in comparison to the control group in which GMC did not change over time (Post Dose 3: GMC of 0.6 µg/mL, 95% CI 0.5 to 0.7 µg/mL) (Table 4 above). Antibody concentrations in this study were higher than those observed previously in The Gambia.

Antibody GMTs to HBsAg after three doses of RTS,S/AS02A (0.25 mL dose) were significantly higher than those observed after 3 doses of Engerix-B: GMT 10 387 (95% CI 4872 to 22 142) compared to GMT 329 (95% CI 108 to 996). One month post Dose 3, seroprotection rates (anti-HBsAg titers $\geq$ 10 mIU/mL) for RTS,S/AS02A (0.25 mL dose) and Engerix-B were 96.3% and 95.2% respectively. There was 1 non-responder in each group (ATP cohort): in the RTS,S/AS02A (0.25 mL dose) group the subject was a chronic carrier of hepatitis B virus, in the Engerix-B group the subject was HBsAg negative.

The experience of the use of RTS,S/AS02A in children to date is that it has an acceptable reactogenicity and safety profile. The 0.25 mL dose is highly immunogenic for anti-CS and anti-HBs antibodies.

1.3.3.3. Phase IIb pediatric trial of RTS,S/AS02A (Malaria-026)

The trial Malaria-026 evaluated the efficacy of the 0.25 mL Dose of RTS,S/AS02A in children aged 1 to 4 years in Mozambique. It was a double-blind randomized design; 2022 children received either 3 doses of the candidate malaria vaccine, containing 25µg RTS,S and 0.25mL AS02A adjuvant, or 3 doses of a control vaccine using a 0, 1 and 2 month vaccination schedule. The control vaccination regime was 3 doses of hepatitis B vaccine (Engerix-B, GlaxoSmithKline Biologicals), administered to children of 24 months and older, or 2 doses of a 7-valent pneumococcal conjugate vaccine (Prevnar®, Wyeth Lederle Vaccines) at Dose 1 and Dose 3 and 1 dose of *Hemophilus influenzae* type b vaccine (Hiberix™, GlaxoSmithKline Biologicals) at Dose 2 to children less than 24 months. Study subjects were recruited to two Cohorts for the study. Cohort 1 included children recruited from the town of Manhiça, Cohort 2 included children from the nearby town of Ilha Josina. Cohort 2 was used to examine vaccine efficacy (VE) against infection.

1.3.3.3.1. Primary Efficacy Endpoint, Malaria-026

The Primary Efficacy Endpoint of the trial was the determination of the VE against clinical malaria disease and was evaluated in Cohort 1. The malaria Case Definition for the Primary Efficacy Endpoint was 'the presence of *P. falciparum* asexual parasitemia above 2500 per µL on Giemsa stained thick blood films and the presence of fever (axillary temperature ≥ 37.5°C) at the time of presentation and occurring in a child who is unwell and brought for treatment to a healthcare facility'.

In Cohort 1 and Cohort 2 the RTS,S/AS02A and control groups were well-balanced for age, gender, bednet usage, distance of residence from health facility and geographical area of residence. Malaria transmission intensity, as indicated by Hct, prevalence of splenomegaly and IFAT, was higher in Ilha Josina where Cohort 2 was evaluated than Manhiça where Cohort 1 was evaluated. Malaria transmission was not constant over the six-month period. Malaria disease rates in the control group were lower in the second half of the observation period.

VE was determined over a six-month surveillance period commencing from 14 days post Dose 3. As specified per RAP, the time at risk was adjusted for malaria drug usage, residence in the study, withdrawal and death and the estimate of effect was adjusted for the covariates of age of the subject, geographical area of the residence of the subject, bednet use, and distance of the subject's residence from the nearest health center. The estimate of VE determined as the time to the first clinical episode in the ATP Cohort for Efficacy (i.e. all children who received full vaccination courses and contributed to the time at risk) was 26.9% (95% CI 7.4% to42.2%; p=0.009) and after adjustment for covariates was 29.9% (95% CI 11.0% to 44.8%; p=0.004).

No waning of efficacy over the 6-month observation period was noted for the primary endpoint when analyzed by different methods (test for proportionality of hazards with Schoenfeld residuals, p=0.139).Consistent with these data, at the cross-sectional survey 6.5 months after Dose 3, prevalence of parasitemia in recipients of RTS,S/AS02A was 37% lower than in the recipients of control vaccines; 11.9% in RTS,S/AS02A vs 18.9%in controls, p=0.0003). Parasite densities in these children were similar between RTS,S/AS02A recipients and controls (geometric mean density 2271 vs 2513; p=0.699).

1.3.3.3.2. Secondary Efficacy Endpoints of clinical malaria disease, Malaria-026

The study evaluated three other Case Definitions of clinical malaria disease specified per protocol. These endpoints explored different parasite density threshold values and history of fever as opposed to documented fever. The point estimates were consistent for all the Secondary Case Definitions of malaria disease.

For the first episode meeting Secondary Case Definition 1 for malaria episodes assessed over 6 months of Dose 3 defined as: the time to the first clinical episode of symptomatic *P. falciparum* malaria meeting Secondary Case Definition 1 for malaria episodes (the presence of *P. falciparum* asexual parasitemia [any level of parasitemia] on Giemsa stained thick blood films *and* the presence of fever [axillary temperature ≥ 37.5°C] at the time of presentation *and* occurring in a child who is unwell and brought for treatment

detected by passive case detection) in children (1–4 years of age at first vaccination), determined over a six-month surveillance period post Dose 3; the VE was 28.6% (95% CI 10.4% to 43.1%; p=0.0036)

First episode meeting Secondary Case Definition 2 for malaria episodes assessed over 6 months of Dose 3: the time to the first clinical episode of symptomatic *P. falciparum* malaria meeting Secondary Case Definition 2 for malaria episodes (the presence of *P. falciparum* asexual parasitemia [any level of parasitemia] on Giemsa stained thick blood films *and* a history of fever within 24 hours or documented fever [axillary temperature ≥ 37.5°C] at the time of presentation *and* occurring in a child who is unwell and brought for treatment) detected by passive case detection in children (1–4 years of age at first vaccination), determined over a six-month surveillance period post Dose 3; the VE was 33.8% (95% CI 19.7% to45.3%; p=<0.001)

First episode meeting Secondary Case Definition 3 for malaria episodes assessed over 6 months of Dose 3: the time to the first clinical episode of symptomatic *P. falciparum* malaria meeting Secondary Case Definition 3 (the presence of *P. falciparum* asexual parasitemia above 15 000 per µL on Giemsa stained thick blood films *and* the presence of fever [axillary temperature ≥ 37.5°C] at the time of presentation *and* occurring in a child who is unwell and brought for treatment to a healthcare facility) detected by passive case detection in children (1–4 years of age at first vaccination), determined over a six-month surveillance period post Dose 3; the VE was 31.7% (95% CI 11.5% to 47.2%; p=0.0039).

1.3.3.3.3. Incidence of moderate anemia, Malaria-026

All children presenting to a Health Center as outpatients who were evaluated for malaria also had a blood sample taken by fingerprick to determine their hematocrit (Hct). It should be noted that there was no requirement for concurrent malaria parasitemia in this case definition and this therefore measures all cause anemia, of which in the study area malaria is the most important cause. The adjusted estimate of VE against first incident case of moderate anemia (defined as Hct < 25%) was 28.2% (95% CI -19.6% to 56.9%; p=0.203) in the ATP Cohort for Efficacy.

1.3.3.3.4. All clinical episodes meeting Primary Case Definition for Malaria, Malaria-026

Relatively few children had second or third episodes of malaria complying with the Primary Case Definition (see Table 7). No child experienced more than 3 episodes of clinical malaria during the course of the trial. The significance of the differences in the distribution of the number of episodes was p=0.0508 assessed using a Fisher exact test.

Table 7 Frequency of children according to the number of episodes by treatment group (ATP Cohort for Efficacy) (Cohort 1), Malaria-026

Number of episodes per child	R(1) N= 745		C(1) N= 745		Total N= 1490		p-value
	Value or n	%	Value or n	%	Value or n	%	
0	622	83.5	586	78.7	1208	81.1	0.0508
1	99	13.3	131	17.6	230	15.4	-
2	18	2.4	25	3.4	43	2.9	-
3	6	0.8	3	0.4	9	0.6	-

R(1): RTS,S/AS02A (Cohort 1, < 24 months)/ RTS,S/AS02A (Cohort 1, ≥ 24 months)
C(1): Prevnar and Hiberix (Cohort 1, < 24 months)/ Engerix-B (Cohort 1, ≥ 24 months)
N=number of subjects
n=number of subjects in a given category
Value=value of the considered parameter
%=n / Number of subjects with available results x 100
p-value: Fisher Exact test

To estimate the VE against all clinical episodes of malaria meeting the Primary Case Definition, the time at risk was adjusted using all of the criteria for the Primary Efficacy Endpoint with the addition of an exclusion period of 28 days after an episode. The adjusted VE against all clinical episodes in the ATP Cohort for Efficacy was 27.3% (95% CI 6.2%to 43.8; p=0.0143).

1.3.3.3.5. Exploratory Efficacy Endpoints of more severe disease, Malaria-026

The malaria transmission season over which the trial was conducted was particularly intense. The increased number of cases (compared to number expected), particularly those at the severe end of the spectrum, allowed the inclusion of Exploratory Efficacy Endpoints to evaluate severe disease to the RAP. It should be emphasized that all case determination was done systematically according to predefined criteria and prior to unblinding.

Malaria requiring hospitalization

Malaria requiring hospitalization was defined as an admission where malaria was either the sole cause of illness or a significant contributing factor. It was analyzed as the proportion of children experiencing one or more episodes during the six-month surveillance period post Dose 3 in the ATP cohort. There were 42 affected children in recipients of RTS,S/AS02A compared to 62 in the controls. The unadjusted estimate of VE was 32.3% (95% CI 1.3% to 53.9%; p=0.053).

Severe malaria disease

The proportion of children affected by one or more episodes of severe malaria meeting the Case Definition was compared. No adjustment was made for covariates. There were 11 cases of severe malaria disease in recipients of RTS,S/AS02A compared to 26 cases in recipients of control vaccine(s); the measured VE was 57.7% (95% CI 16.2% to 80.6%, p=0.019).

Total number of hospital admissions

The analysis of the total number of hospital admissions is restricted to those hospital admissions which occurred in the six-month surveillance period and in children belonging to the ATP Cohort for Efficacy, Cohort 1. In total there were 79 admissions in the RTS,S/AS02A group and 90 in the control Table 8. The Fisher exact test showed no statistical significance difference between the groups.

Table 8 Frequency distribution of all-cause hospital admissions (ATP Cohort for Efficacy), Malaria-026

Characteristics	Parameters or Categories	R(1) N= 745		C(1) N= 745		Total N= 1490		p-value
		Value or n	%	Value or n	%	Value or n	%	
Hospital admissions	0	678	91.0	665	89.3	1343	90.1	0.5281
	1	56	7.5	71	9.5	127	8.5	-
	2	10	1.3	8	1.1	18	1.2	-
	3	1	0.1	1	0.1	2	0.1	-

R(1): RTS,S/AS02A (Cohort 1, < 24 months)/ RTS,S/AS02A (Cohort 1, ≥ 24 months)
C(1): Prevnar and Hiberix (Cohort 1, < 24 months)/ Engerix-B (Cohort 1, ≥ 24 months)
N=number of subjects
n=number of subjects in a given category
Value=value of the considered parameter
%=n / Number of subjects with available results x 100
p-value: Fisher Exact test

An analysis was performed comparing the rate of all hospital admissions. The time at risk was adjusted in a similar way to the time at risk for the analysis except that there was no adjustment for antimalarial drug usage and absences from the study area. The estimate was not adjusted for covariates. The unadjusted estimate of VE was 14.4% (95% CI -19.7% to 38.8%; p=0.362).

1.3.3.3.6. Association of CS response with efficacy, Malaria-026

As specified in the RAP, two approaches to the analysis of the association of the CS response with hazard rate in the RTS,S/AS02A recipients were used. The association between CS and Hazard Rate by means of a comparison of hazard rates between the higher tertile (measured in the treated group) against the lower tertile of the anti-CS antibodies as well as the hazard rates per ten-fold increase in the value of anti-CS antibodies was calculated. Neither analysis detected an association reaching statistical significance (see Table 9)

Table 9 Time to the first episode of clinical malaria according to anti-CS value (ATP Cohort for Efficacy) (Cohort 1), Malaria-026

Label	Hazard Ratio	LL	UL	p-value
Per 10-fold increase	0.94	0.66	1.33	0.7079
Higher versus Lower Tertile	1.38	0.89	2.12	0.1498

LL=95% lower limit
UL=95% upper limit
p-value from Cox proportional hazards model

1.3.3.3.7. Efficacy analysis in the ITT Cohort from Day 0, Malaria-026

For safety reasons, the FDA requested the analyses from Day 0 be conducted on the Primary Case Definition in the ITT Cohort. This was repeated for all clinical attacks fitting the Primary Case Definition and the incidence of anemia. For the analysis of the ITT Cohort the time at risk was not adjusted for temporary travel from the study area, malaria drug usage or for previous clinical episodes, and no adjustment was made for covariates.

The VE for the Primary Case Definition was 30.2% (95% CI 14.4% to 43.0%; $p < 0.001$). The estimate of VE against all clinical episodes was 33.5% (95% CI 16.4% to 47.1%; $p < 0.001$). The estimate for VE against incidence of anemia was 24.8% (95% CI -12.8% to 49.9%; p=0.168). The estimate for VE for severe malaria disease was 48.5% (95% CI 7.3% to 73.1%; p=0.027); for hospitalized malaria the VE was estimated to be 31.2% (95% CI 3.1% to 51.5%; p=0.032).

The results in the ITT Cohort from Day 0 were consistent with the results of the ATP Cohort for Efficacy post Dose 3 for all the endpoints analyzed

1.3.3.3.8. Efficacy against infection, Malaria-026

The first infection could be detected actively on a scheduled home visit or at the time a child presented at a health facility with clinical malaria. All children received sulfadoxine-pyrimethamine and amodiaquine 14 days prior to Dose 3 and only children with a negative blood slide for malaria parasites were included in the ATP Cohort for Efficacy. As specified in the RAP, the time at risk was adjusted for malaria drug usage, residence in the study, withdrawal and death. The unadjusted estimate of VE determined as the time to the first infection in the ATP Cohort for Efficacy (i.e. all children who received full vaccination courses and contributed to the time at risk) was 44.7% (95% CI 31.0% to 55.6%; $p \leq 0.001$). After adjustment for the covariates of age of subject, bednet usage and distance of residence of subject from a health center, the VE was 45.0% (95% CI 31.4% to 55.9%; $p \leq 0.001$).

1.3.3.3.9. Immunogenicity, Malaria-026

Full details of the CS and HBs responses for Malaria-026 can be found in Table 4 and Table 5 respectively.

A formal study of non-inferiority was completed in children 2 to 4 years of age in Malaria-026. Table 10 shows the assessment of non-inferiority of RTS,S/AS02A

compared to Engerix-B with respect to seroprotection rates in subjects HBsAg negative pre-vaccination. The lower limit of the 95% CI for the difference in anti-HBs seroprotection rates (≥10 mIU/ml) was 4.3%, hence greater than -10%, thus demonstrating non-inferiority of RTS,S/AS02A compared to Engerix-B.

Table 10 Difference between groups in percentage of HBsAg negative subjects with anti-HBs titer at least 10 mIU/ml at month 3, post Dose 3 (ATP cohort for immunogenicity)

						Inference			
							Value	95% CI	
Group	N	%	Group	N	%	Diff	%	LL	UL
C(2)>24	111	91.9	R(2)>24	126	100.0	R(2)>24 - C(2)>24	8.1	4.3	14.7

R(2)>24 : RTS,S/AS02A (Cohort 2, ≥24 months)
C(2)>24 : Engerix-B (Cohort 2, ≥24 months)
N = number of subjects with available results
% = percentage of subjects with anti-HBs titer ≥ 10 mIU/ml
Diff = Difference calculated
CI = Standardized asymptotic confidence interval

Table 11 shows the assessment of non-inferiority of RTS,S/AS02A compared to Engerix-B with respect to the ratio of anti-HBs GMTs in HBsAg negative subjects at baseline. The upper limit of the 95% CI for the ratio of anti-HBs GMTs was 0.04, hence lower than 2.0, thus demonstrating non-inferiority of RTS,S/AS02A compared to Engerix-B.

Table 11 Non-inferiority in terms of GMTs in HBsAg negative subjects at Month 3 for the serotest anti-HBs - Point estimate and CI for the relative difference of GMTs and the ratio of GMTs (ATP cohort for immunogenicity)

	N in C(2)	N in R(2)	Ratio	95% CI	
				LL	UL
C(2)>24 vs R(2)>24	111	126	0.02754	0.0176	0.0431

C(2)>24 : Engerix-B (Cohort 2, ≥24 months)
R(2)>24 : RTS,S/AS02A (Cohort 2, ≥24 months)

A protection rate of > 97% was achieved. Again, RTS,S/AS02A was found to be highly immunogenic for anti-HBs antibodies. At 30 days post Dose 3 of RTS,S/AS02A, GMTs of 51 035 (95% CI 27 919 to 93 292) were observed in recipients of RTS,S/AS02A younger than 24 months of age; for children older than 24 months, GMTs of 11 369 were observed (8519 to 15 172) at the same timepoint. GMTs remained high at 180 days post Dose 3: 13 642 (95% CI 7342 to 25 347) in children younger than 24 months, 4556 (3500 to 5932 in children older than 24 months). The response to HBsAg was approximately 32 fold greater with RTS,S/AS02A compared to Engerix-B. Safety and reactogenicity, Malaria-026

Solicited symptoms are shown in Table 12 and Table 13, corresponding to results from Cohort 1 and Cohort 2 respectively. As with previous studies with RTS,S/AS02A, local reactions at the site of injection were common. Pain was the most frequently observed local symptom. In the recipients of RTS,S/AS02A, the most commonly observed solicited general adverse event related to vaccination was fever (In Cohort 1, 11.1% after all doses in children < 24 months of age, 3.7% after all doses in children ≥ 24 months of

age: in Cohort 2 related fever was observed after 4.9% of doses in children < 24 months, 1.3% of doses in children ≥ 24 months). In recipients of control vaccines in Cohort 1, fever was also the most commonly observed solicited adverse event related to vaccination (1.1% in children < 24 months of age [who received Prevnar and Hiberix] and 0.9% in children > 24 months of age [who received Engerix-B]). For recipients of RTS,S/AS02A < 24 months of age, a trend of increased local reactogenicity with sequential doses was observed. For recipients of RTS,S/AS02A ≥ 24 months of age, local reactogenicity was most frequent following Dose 3. The incidence of general symptoms of drowsiness, irritability, loss of appetite and fever (≥ 37.5°C) was higher in recipients of RTS,S/AS02A than recipients of control vaccine; Grade 3 events were infrequent. In children < 24 months, the most commonly reported AEs were Upper Respiratory Tract infection, Malaria and Diarrhea and in children ≥ 24 months Upper Respiratory Tract Infection (URTI), Malaria and Ascariasis; frequencies were similar in the RTS,S/AS02A and control groups.

Table 12 Frequency of solicited symptoms per dose during the 4-day follow-up period for each vaccine group (Total Cohort, Cohort 1, Malaria-026)

**Dose**		**COHORT 1 All/Dose**															
Group		R(1)<24				C(1)<24				R(1)>24				C(1)>24			
N		558				551				1768				1768			
		n	%	95% CI		n	%	95% CI		n	%	95% CI		n	%	95% CI	
				LL	UL			LL	UL			LL	UL			LL	UL
Pain	Any	165	29.6	25.8	33.5	43	7.8	5.7	10.4	459	26.0	23.9	28.1	78	4.4	3.5	5.5
	Grade 3	2	0.4	0.0	1.3	0	0.0	0.0	0.7	3	0.2	0.0	0.5	1	0.1	0.0	0.3
Swelling	Any	133	23.8	20.4	27.6	54	9.8	7.4	12.6	314	17.8	16.0	19.6	54	3.1	2.3	4.0
	Grade 3	45	8.1	5.9	10.6	8	1.5	0.6	2.8	104	5.9	4.8	7.1	1	0.1	0.0	0.3
Drowsiness	Any	38	6.8	4.9	9.2	15	2.7	1.5	4.5	46	2.6	1.9	3.5	25	1.4	0.9	2.1
	Grade 3	5	0.9	0.3	2.1	1	0.2	0.0	1.0	5	0.3	0.1	0.7	2	0.1	0.0	0.4
	Rel* Grade 3	0	0.0	0.0	0.7	0	0.0	0.0	0.7	0	0.0	0.0	0.2	0	0.0	0.0	0.2
Irritability	Any	73	13.1	10.4	16.2	14	2.5	1.4	4.2	53	3.0	2.3	3.9	22	1.2	0.8	1.9
	Grade 3	4	0.7	0.2	1.8	1	0.2	0.0	1.0	3	0.2	0.0	0.5	3	0.2	0.0	0.5
	Rel* Grade 3	0	0.0	0.0	0.7	0	0.0	0.0	0.7	0	0.0	0.0	0.2	0	0.0	0.0	0.2
Loss of Appetite	Any	101	18.1	15.0	21.6	33	6.0	4.2	8.3	130	7.4	6.2	8.7	62	3.5	2.7	4.5
	Grade 3	6	1.1	0.4	2.3	2	0.4	0.0	1.3	4	0.2	0.1	0.6	3	0.2	0.0	0.5
	Rel* Grade 3	0	0.0	0.0	0.7	0	0.0	0.0	0.7	0	0.0	0.0	0.2	0	0.0	0.0	0.2
Temperature	Any	152	27.2	23.6	31.1	32	5.8	4.0	8.1	173	9.8	8.4	11.3	91	5.1	4.2	6.3
	Grade 3	10	1.8	0.9	3.3	1	0.2	0.0	1.0	7	0.4	0.2	0.8	6	0.3	0.1	0.7
	Rel* Grade 3	4	0.7	0.2	1.8	0	0.0	0.0	0.7	1	0.1	0.0	0.3	1	0.1	0.0	0.3

R(1)<24; recipients of RTS,S/AS02A less than 24 months of age (Cohort 1)
C(1)<24; recipients of control vaccines (Prevnar & Hiberix) less than 24 months of age (Cohort 1)
R(1)>24; recipients of RTS,S/AS02A greater than or equal to 24 months of age (Cohort 1)
C(1)>24; recipients of control vaccines (Engerix-B) greater than or equal to 24 months of age (Cohort 1)
N = number of documented doses.
n/% = number/percentage of doses followed by a local/general symptom.
*axillary body temperature ≥ 37.5°C.
Grade 3 injection site pain; cries when limb is moved/spontaneously painful.
Grade 3 swelling; > 20 mm.
Grade 3 fever; axillary body temperature ≥ 39.0°C.
Grade 3 drowsiness; drowsiness that prevents normal activity.
Grade 3 loss of appetite; not eating at all.
Grade 3 irritability/fussiness; crying that cannot be comforted/prevents normal activity.
Rel = related to study vaccine.
Note: all local injection site symptoms were considered related to study vaccine.

Table 13 Frequency of solicited symptoms per dose during the 4-day follow-up period for each vaccine group (Total Cohort, Cohort 2, Malaria-026)

**Dose**		**COHORT 2 All/Dose**															
Group		R(2)<24				C(2)<24				R(2)>24				C(2)>24			
N		143				144				457				449			
		n	%	95% CI		n	%	95% CI		n	%	95% CI		n	%	95% CI	
				LL	UL			LL	UL			LL	UL			LL	UL
Pain	Any	40	28.0	20.8	36.1	9	6.3	2.9	11.5	80	17.5	14.1	21.3	12	2.7	1.4	4.6
	Grade 3	0	0.0	0.0	2.5	0	0.0	0.0	2.5	2	0.4	0.1	1.6	0	0.0	0.0	0.8
Swelling	Any	48	33.6	25.9	41.9	12	8.3	4.4	14.1	113	24.7	20.8	28.9	18	4.0	2.4	6.3
	Grade 3	28	19.6	13.4	27.0	3	2.1	0.4	6.0	47	10.3	7.7	13.4	2	0.4	0.1	1.6
Drowsiness	Any	14	9.8	5.5	15.9	8	5.6	2.4	10.7	14	3.1	1.7	5.1	7	1.6	0.6	3.2
	Grade 3	1	0.7	0.0	3.8	0	0.0	0.0	2.5	3	0.7	0.1	1.9	0	0.0	0.0	0.8
	Rel* Grade 3	0	0.0	0.0	2.5	0	0.0	0.0	2.5	0	0.0	0.0	0.8	0	0.0	0.0	0.8
Irritability	Any	19	13.3	8.2	20.0	2	1.4	0.2	4.9	13	2.8	1.5	4.8	4	0.9	0.2	2.3
	Grade 3	1	0.7	0.0	3.8	0	0.0	0.0	2.5	0	0.0	0.0	0.8	0	0.0	0.0	0.8
	Rel* Grade 3	0	0.0	0.0	2.5	0	0.0	0.0	2.5	0	0.0	0.0	0.8	0	0.0	0.0	0.8
Loss of Appetite	Any	27	18.9	12.8	26.3	12	8.3	4.4	14.1	28	6.1	4.1	8.7	10	2.2	1.1	4.1
	Grade 3	1	0.7	0.0	3.8	0	0.0	0.0	2.5	2	0.4	0.1	1.6	0	0.0	0.0	0.8
	Rel* Grade 3	0	0.0	0.0	2.5	0	0.0	0.0	2.5	0	0.0	0.0	0.8	0	0.0	0.0	0.8
Temperature	Any	40	28.0	20.8	36.1	9	6.3	2.9	11.5	57	12.5	9.6	15.9	48	10.7	8.0	13.9
	Grade 3	2	1.4	0.2	5.0	1	0.7	0.0	3.8	1	0.2	0.0	1.2	3	0.7	0.1	1.9
	Rel* Grade 3	0	0.0	0.0	2.5	0	0.0	0.0	2.5	0	0.0	0.0	0.8	0	0.0	0.0	0.8

R(2)<24; recipients of RTS,S/AS02A less than 24 months of age (Cohort 2)
C(2)<24; recipients of control vaccines (Prevnar & Hiberix) less than 24 months of age (Cohort 2)
R(2)>24; recipients of RTS,S/AS02A greater than or equal to 24 months of age (Cohort 2)
C(2)>24; recipients of control vaccines (Engerix-B) greater than or equal to 24 months of age (Cohort 2)
N = number of documented doses.
n/% = number/percentage of doses followed by a local/general symptom.
*axillary body temperature ≥ 37.5°C.
Grade 3 injection site pain; cries when limb is moved/spontaneously painful.
Grade 3 swelling; > 20 mm.
Grade 3 fever; axillary body temperature ≥ 39.0°C.
Grade 3 drowsiness; drowsiness that prevents normal activity.
Grade 3 loss of appetite; not eating at all.
Grade 3 irritability/fussiness; crying that cannot be comforted/prevents normal activity.
Rel = related to study vaccine.
Note: all local injection site symptoms were considered related to study vaccine.

Fifteen subjects died during the double-blind phase of the Malaria-026 study; 5 had received RTS,S/AS02A and 10 had received control vaccines. Four deaths of recipients of control vaccines had malaria as a sole or contributing factor; no deaths of recipients of RTS,S/AS02A had malaria as a contributing factor. None of the deaths were, in the judgement of the investigator, related to vaccination. Extrapolating from the mortality rate in the study population, approximately 17 deaths would be expected (this calculation assumes an adjusted seasonal rate of 24 per 1000 cyr [personal communication, Pedro Alonso, October 2003]) and 1010 child-years of observation approximately 17 July 2003 through 29 March 2004). The observed rate is lower and can be attributed to the ‘healthy cohort effect’; only fit children were enrolled in the trial and they remained under close medical surveillance.

There was a tendency for the proportion of children experiencing an SAE to be lower in the recipients of RTS,S/AS02A compared to recipients of control vaccines. In Cohort 1, for recipients of RTS,S/AS02A < 24 months of age, 25% of children suffered at least one SAE; for recipients of RTS,S/AS02A ≥ 24 months of age, 14% of subjects suffered at

least one SAE. This is compared to recipients of Prevnar and Hiberix (children < 24 months of age), 37% of whom suffered at least one SAE; for recipients of Engerix-B (those children ≥ 24 months of age) 20% of subjects suffered at least one SAE. It should be noted that apart from five deaths that occurred at home, the non-hospitalized SAEs were all children with episodes of acute malaria associated with a high parasite density (defined as 5+ parasitemia on quick read).

There were no SAEs judged related to vaccination.

In Cohort 1, SAE reports of malaria tended to be fewer in recipients of RTS,S/AS02A. In children <24 months who received RTS,S/AS02A 19% of children experienced a malaria episode compared to 33% in the control group. There was a similar trend in the children ≥ 24 months; in the recipients of RTS,S/AS02A 13% experienced a malaria episode compared to 16% in the recipients of control vaccines. In Cohort 2, where surveillance for malaria was intense, no difference was observed. For the other SAEs classified at the MedDRA preferred term level, there were no differences in the pattern of morbidity observed between recipients of RTS,S/AS02A and recipients of control vaccines. The pattern of morbidity and SAEs in the study participants was similar to that previously observed at the study site (personal communication, Dr. Pedro Alonso, October 2003) and that has been described in the region [Fidel, 2002; Iriso, 2000]..

1.3.3.4. Phase II pediatric trial of RTS,S/AS02D; Bridging Study (Malaria-034)

Malaria-034 was a phase I/II randomized double-blind bridging study to evaluate the safety and immunogenicity of 3 doses of GlaxoSmithKline Biologicals’ candidate vaccine RTS,S/AS02D (0.5 mL dose) in comparison to 3 doses of the existing formulation RTS,S/AS02A (0.25 mL dose) administered to children aged 3 to 5 years living in a malaria-endemic region of Mozambique. Both vaccines contain the same constituents but the final volume was adjusted to be compatible with existing EPI practices.

Two hundred children were enrolled into two groups of 100 subjects each, of which 189 (93 of whom received RTS,S/AS02D [D group] and 96 received RTS,S/AS02A [A group]) subjects were included in the ATP analysis of safety and reactogenicity and 143 (67 and 76 subjects respectively) subjects in the analysis of immunogenicity.

The safety profile of the two vaccine formulations was similar. Solicited symptoms were as shown in Table 14, with pain the more frequent local symptom and fever the most frequent general symptom reported. Symptoms were generally mild to moderate in intensity, and decreased in frequency with subsequent doses.

Unsolicited symptoms were reported following 69 (24.7%) doses in the RTS,S/AS02D group and following 56 (19.4%) in the RTS/SAS02A group. The most frequently reported symptoms were malaria (18% of the subjects in the D group and 25% in the A group) and upper respiratory tract infection (14% of the subjects in the D group and 12% in the A group). An unsolicited symptom, injection site erythema, deemed to be related to vaccination was reported for a single subject, following one dose in the D group. Three

symptoms in the D group and 8 in the A group were graded 3 in intensity, none of which were related to vaccination.

One serious adverse event occurred in a subject in the A group. This subject had severe malaria which lasted 4 days and required hospitalization. The subject recovered and the event was deemed by the investigator to be unrelated to vaccination.

Table 14: Incidence of any and grade 3 local and general solicited symptoms in the 7-day follow-up after vaccination

Group		RTS,S/AS02D (N = 93)				RTS,S/AS02A (N = 96)			
		n	%	95% CI		n	%	95% CI	
Pain	Any	31	33.3	23.9	43.9	43	44.8	34.6	55.3
	Grade 3	2	2.2	0.3	7.6	1	1.0	0.0	5.7
Swelling	Any	8	8.6	3.8	16.2	23	24.0	15.8	33.7
	Grade 3	1	1.1	0.0	5.8	4	4.2	1.1	10.3
Drowsiness	Any	12	12.9	6.8	21.5	8	8.3	3.7	15.8
	Grade 3	2	2.2	0.3	7.6	1	1.0	0.0	5.7
Fever	Any	26	28.0	19.1	38.2	25	26.0	17.6	36.0
	Grade 3	6	6.5	2.4	13.5	3	3.1	0.6	8.9
Irritability	Any	11	11.8	6.1	20.2	3	3.1	0.6	8.9
	Grade 3	3	3.2	0.7	9.1	1	1.0	0.0	5.7
Loss of Appetite	Any	15	16.1	9.3	25.2	14	14.6	8.2	23.3
	Grade 3	0	0.0	0.0	3.9	0	0.0	0.0	3.8

For each dose and overall/subject:
95%CI = Exact 95% confidence interval
N = number of subjects having received at least one dose
n/% = number/percentage of subjects reporting a specified symptom
Grade 3 injection site pain; cries when limb is moved/spontaneously painful.
Grade 3 swelling; > 20 mm.
Fever: axillary body temperature ≥ 37.5°C.
Grade 3 fever; axillary body temperature ≥ 39.0°C.
Grade 3 drowsiness; drowsiness that prevents normal activity.
Grade 3 loss of appetite; not eating at all.
Grade 3 irritability/fussiness; crying that cannot be comforted/prevents normal activity

Non-inferiority of the RTS,S/AS02D vaccine formulation was proven with respect to the immune response elicited. Table 15 shows the anti-CS seropositivity rates and GMTs one month following the third vaccine dose and Table 16 gives the ratios of post vaccination GMTs. All subjects in both groups were seropositive for anti-CS antibodies one month following the third vaccine dose. The RTS,S/AS02A:RTS,S/AS02D GMT ratio was 0.9 (clinical limit of non-inferiority: upper limit of 95% CI = 3).

Table 15 Seropositivity rates and geometric mean titers (GMT) for anti-CS antibody titers one month after Dose 3 (ATP cohort for immunogenicity)

Group	N	≥ 0.5 EU/mL				GMT			MIN	MAX
				95% CI		Value	95% CI			
		n	%	LL	UL		LL	UL		
RTS,S/AS02D	67	67	100.0	94.6	100.0	190.9	150.3	242.4	14.3	1314.3
RTS,S/AS02A	76	76	100.0	95.3	100.0	179.6	145.9	221.0	16.3	1276.9

N = number of subjects with available results
GMTs calculated on all subjects
EU/mL: ELISA units per mL
n/% = number/percentage of subjects with titer within the specified range
95% CI = 95% confidence interval; LL = Lower Limit; UL = Upper Limit
MIN/MAX = Minimum/Maximum
PRE = pre-vaccination
PIII (M3) = Post dose 3 Month 3

Table 16 Ratios of post-vaccination anti-CS GMT at PIII (M3) between Group A and Group D with their 95% CIs (ATP cohort for immunogenicity)

Antibody	RTS,S/AS02A		RTS,S/AS02D		Ratio (Group A/Group D)		
	N	GMT	N	GMT	GMT ratio	95% CI	
						LL	UL
ANTI-CS	76	179.6	67	190.9	0.9	0.7	1.3

N: Number of subjects with available results
95% CI LL, UL: 95% lower and upper confidence interval limits (ANOVA model)

All subjects were seroprotected with respect to anti-HBs after vaccination, as shown in Table 17, and GMTs exceeded 17 000 mIU/ml in both groups. The RTS,S/AS02A/RTS,S/AS02D ratio of post-vaccination anti-HBs GMTs (Table 18), and adjusted ratio were 0.7 and 0.8 respectively (clinical limit of non-inferiority: upper limit of 95% CI = 3).

Table 17 Seroprotection rates and geometric mean titers (GMT) for anti-HBs antibody titers one month after dose 3 (ATP cohort for immunogenicity)

Group	N	≥ 10 mIU/ML				GMT			MIN	MAX
				95% CI		Value	95% CI			
		n	%	LL	UL		LL	UL		
RTS,S/AS02D	67	67	100.0	94.6	100.0	23977.6	17895.5	32126.9	984.9	446880.0
RTS,S/AS02A	76	76	100.0	95.3	100.0	17410.0	13322.3	22751.9	1264.5	823500.0

N = number of subjects with available results
GMTs calculated on all subjects
n/% = number/percentage of subjects with titer within the specified range
95% CI = 95% confidence interval; LL = Lower Limit; UL = Upper Limit
MIN/MAX = Minimum/Maximum
PRE = pre-vaccination
PIII (M3) = Post dose 3 Month 3

Table 18 Ratios of post-vaccination anti-HBs GMT at PIII (M3) between Group A and Group D with their 95% CIs (ATP cohort for immunogenicity)

Antibody	RTS,S/AS02A		RTS,S/AS02D		Ratio (Group A/Group D)		
	N	GMT	N	GMT	GMT ratio	95% CI	
						LL	UL
Anti-HBs	76	17410.0	67	23977.6	0.7	0.5	1.1

N: Number of subjects with available results
95% CI LL, UL: 95% lower and upper confidence interval limits (ANOVA model)

1.3.4. Concurrent protection against hepatitis B

The hepatitis B surface antigen (HBsAg) contained in the RTS,S candidate malaria and hepatitis B vaccine is encoded by the hepatitis B virus S protein gene that is identical to the gene used to express HBsAg in GSK Biologicals' Engerix-B vaccine against hepatitis B.

Non-inferiority of the HBsAg response of RTS,S/AS02A compared to the licensed vaccine Engerix-B has been formally demonstrated in the study Malaria-026 [Alonso, 2004] (refer to Section 1.3.3.3). The RTS,S/AS02D vaccine has been shown to induce high levels of seroprotection and titers in the study Malaria-034 (refer to Section 1.3.3.4)

1.4. Rationale for the study design

There are two reasons why the RTS,S/AS02D candidate malaria and hepatitis B vaccine is being developed for delivery through the infant Expanded Program on Immunization (EPI) of the World Health Organization (WHO). Firstly, the vaccine is being developed to prevent severe malaria disease which occurs from about 4 months of age coinciding with the time that maternally-acquired immunity wanes [Snow 1990, Snow 1994]. Secondly the EPI has been highly successful at increasing the coverage of basic vaccines across the developing world. With the goal of integration into the EPI infant regimen, a 0, 1, 2 month schedule has been evaluated in children and the final dose volume has been adjusted to 0.5 mL to be compatible with the standard syringes used by EPI (see Section 1.3.2).

Although RTS,S/AS02D is primarily being developed as a vaccine against malaria, the particles of RTS,S contain HBsAg and the vaccine induces high seroprotection rates against hepatitis B (see Section 1.2.2). Therefore it is logical to propose that, in malaria endemic regions, RTS,S/AS02D replaces the existing hepatitis B vaccine in the infant regimen. This approach has the advantage that adding malaria immunization to the EPI schedule requires no extra vaccination clinic visits in the first year of life, which is more practical for parents and EPI services.

As the next step in the development path, this study examines the feasibility of giving RTS,S/AS02D to infants in the EPI age range; three doses of RTS,S/AS02D will be given at 10, 14 and 18 weeks of age, staggered with the administration of DTPw/Hib at 8, 12 and 16 weeks of age. The control arm will receive Hepatitis B vaccine at the times RTS,S/AS02D is given. As this is the initial assessment of vaccine safety in infants, the conduct of this study will precede studies of the coadministration of RTS,S/AS02D with

EPI vaccines. In this study, the licensed vaccines used are Engerix-B® (GlaxoSmithKline Biologicals) and TETRActHib™ (Aventis Pasteur).

Because this is an experimental regimen of hepatitis B immunization, infants at high risk of hepatitis B infection will be excluded from the trial. Infants born to women who are chronic carriers of hepatitis B virus (HBV) will receive the licensed vaccine in a regimen starting at birth (refer to Section 5.1.10.2.1).

Infants who are born to mothers who test positive for HIV will not be enrolled in the trial. HIV positive children may not respond normally to standard EPI vaccines and thus interfere with the analysis of immunogenicity in this study [Ryder et al 1993]. Also the high morbidity experienced by HIV positive children may obscure safety patterns.

Oral polio vaccine (OPV) will be administered according to local medical practice (birth, 8, 12, 16 weeks co-administration with other EPI vaccines) and will not be administered as part of this protocol. Antibody titers to OPV will not be assessed as part of this protocol but will be evaluated in Phase 3 development.

1.4.1. Safety monitoring plan

This study is the first administration of RTS,S/AS02D to infants and therefore a detailed safety plan, overseen by a formally constituted Data Safety Monitoring Board (DSMB) operating under a charter, is implemented (the role of the DSMB is fully detailed in Section 5.1.3). The DSMB is empowered to temporarily suspend the trial on the basis of any safety concern or meeting predefined criteria for temporary suspension (Section 5.1.6).

The rate of enrolment is uncertain as it is dependent on the birth rate, and could range from 6 to 12 months. The DSMB will receive all SAEs within 24 hours. In addition, the DSMB will review summarized safety and reactogenicity data at protocol-defined timepoints, so that enrollment to the trial could be halted in a timely fashion if a safety issue was detected (refer to Section 5.1.5).

1.4.1.1. Safety data collection on TETRActHib

Comprehensive safety data on the licensed vaccine TETRActHib will be collected under this protocol in an open fashion. This data that is collected in the same children, at the same time, by the same methodology as the data on RTS,S/AS02D will provide a useful bench mark in the interpretation of reactogenicity of RTS,S/AS02D.

1.4.2. Assessment of proof of concept

Prior to initiating definitive studies of vaccine efficacy in infants that will require larger sample sizes, the minimum number of infants will be enrolled in this study to achieve proof-of-concept of vaccine efficacy. Proof-of-concept will be assessed as time-delay to first infection with asexual forms of *P. falciparum*.

The time to infection was assessed following homologous challenge in the challenge model conducted at the WRAIR; estimates of vaccine efficacy with RTS,S/AS02 in field trials were consistently approximately 40% [Malaria-026, Malaria-005]. When the vaccine was evaluated under conditions of natural exposure in The Gambia [Malaria-005], the vaccine efficacy for the time to first infection in adults was 34% (CI 8.0% to 53.0%). The recently conducted Phase 2b trial in Mozambican children (Malaria-026) was the first study in which both clinical and parasitological endpoints were assessed. Measured concurrently in two groups of children, vaccine efficacy against first clinical episode was 30% (95% CI: 11.0% to 44.8%) and against first infection was 45% (95% CI: 31.4% to 55.9%).

Time to first infection will be used to assess proof-of-concept in infants, because it has proven to be consistent when measured by different methodologies in different populations and because, in the recent Malaria-026 study, the point estimate of efficacy against infection was consistent with the estimate of prevention of clinical episodes.

~~1.5. Rationale for studying the determinants of vaccine response~~

~~Molecular techniques provide the means to address the problems of individual and group variations in specific vaccine responses, and to gain further insight into the general mechanisms of response to vaccines. These technologies have recently undergone substantial development and are now able to successfully dissect the mechanisms in human disease.~~

~~This study of the response to malaria vaccination will examine two types of genes. The first group would be candidate genes known to be important in inflammatory or immunological responses to infection with malaria and these should be investigated with respect to the potential effects of variations in these genes. Candidate genes specific for malaria vaccination responses would include genes that: (1) are known to be directly involved in infection with malaria; (2) have common polymorphisms that affect the amount or function of the protein produced by the gene; or (3) are related to a specific marker of infection with malaria. A list of potential candidate genes is provided below (Table 19). To date, there have been no reports examining the role of these or other polymorphisms on response or non-response to malaria vaccines. The second group of genes are those along the immunological pathways that are most likely to be important in protection from infection and the most obvious of these are the Th1/Th2 immune response pathways. Indeed, at the early time of life that vaccination is given, there is a general delay in establishment of Th1 responses and the initial response to vaccine antigens is initially strongly Th2 biased [Rowe, 2000]. A list of genes that are directly or indirectly involved in Th1/Th2 responses is provided below (Table 19).~~

Table 19 Candidate genes for determinants of vaccine response study

Candidate genes for malaria infection	Genes involved directly or indirectly with Th1/Th2 immunity
Intercellular adhesion molecule 1 (ICAM-1)	Gly241Arg, Glu469Lys
IL-10	1082G/A, 819C/T, 592C/A
CD36	14T/C, 53G/T, 1439G/C
CD14	159C/T, 1359G/T, 1145A/G
Inducible nitric oxide synthase (iNOS)	1173C/T
IL-4	1098T/G, 589C/T, 33C/T
Complement receptor 1 (CR1)	3650A/G
IL-4R alpha	Ile50Val, Gln551Arg, Ser478Pro
Mannose-binding protein (MBP)	Gly54Asp, Gly57Glu
IL-13	1111C/T, Arg130Gln, 4738G/A
Tumor necrosis factor alpha (TNF alpha)	308G/A
TLR4	2026A/T, 1607T/C, Asp299Gly, Thr399Ile
Toll-like Receptor (TLR2)	Arg677Trp, Arg753Gln
IL-1beta	511C/T, 3953C/T
IL-6	174G/C, 572G/C
IL-12B	1188A/C
IFN gamma receptor 1 (IFN gamma R1)	56T/C

A blood sample for the determination of determinants of vaccine response will be taken during this trial (see Section 5.3) and analyzed according to CISM SOP.

(Amended 12 April 2005).

1.6. Rationale for testing cell-mediated immunity

RTS,S/AS02A has been shown to be a powerful inducer of antigen-specific humoral and CMI response in preclinical and clinical studies [GSK data on file, Malaria Investigator’s Brochure 2003]. The RTS,S/AS02D vaccine is believed to elicit a strong neutralizing humoral immune response directed against surface-exposed sporozoite proteins, and elicit CMI responses characterized by predominantly CD4+—Th1 cells, that are hypothesized to either destroy infected hepatocytes and/or limit intracellular parasite development through appropriate cytokines.

CMI response induced by a vaccine antigen formulated in AS02 adjuvant has never before been measured in infants. As it is believed to be a key component in protecting vaccinees against the *P. falciparum* parasite it will be measured in this trial. We will compare the CMI response of infants vaccinated with RTS,S/AS02D against the response of infants vaccinated with Engerix-B prior to vaccination, one month post Dose 3 and 3 months post Dose 3. The characterization of the specific CMI response will be performed as exploratory readouts and will address the most appropriate cellular markers.

1.7. Rationale for Interim Analysis

This study is the first administration of RTS,S/AS02D to infants. The interim analysis scheduled to be carried out 1 month post Dose 3 of RTS,S/AS02D or Engerix-B will provide safety and immunogenicity data prior to the final study report becoming available. The resulting safety and immunogenicity data will be used by clinical

development staff at GSK Biologicals and MVI to check the assumptions on which the future clinical development of the candidate RTS,S/AS02D vaccine is based, and allow for any necessary amendments (Amended 29 June 2005).

2. OBJECTIVE(S)

2.1. Primary: Safety

- To describe the safety and reactogenicity of RTS,S/AS02D administered as 3 doses intramuscularly in the left thigh to infants at 10, 14 and 18 weeks of age, staggered with the administration of 3 doses of TETRActHib intramuscularly in the right thigh at 8, 12 and 16 weeks of age.

Refer to Section 10.1 for definition of the primary endpoint.

2.2. Secondary: Immunogenicity

- To demonstrate the non-inferiority of antibody responses to Hepatitis B, when administered as 3 doses of RTS,S/AS02D at 10, 14 and 18 weeks of age compared to the regimen of 3 doses of Engerix-B at the same ages.

- To describe antibody responses to the circumsporozoite (CS) antigen of the RTS,S/AS02D malaria candidate vaccine administered as 3 doses intramuscularly in the left thigh to infants at 10, 14 and 18 weeks of age

Refer to Section 10.2 for definitions of secondary endpoints for immunogenicity.

2.3. Secondary: Proof-of-concept

- To assess the efficacy of RTS,S/AS02D against infection (defined as *P. falciparum* asexual parasitemia > 0) with *P. falciparum* malaria in infants immunized with RTS,S/AS02D as 3 doses at 10, 14 and 18 weeks of age

Refer to Section 10.2 for definitions of secondary endpoints for proof-of concept.

3. STUDY DESIGN OVERVIEW

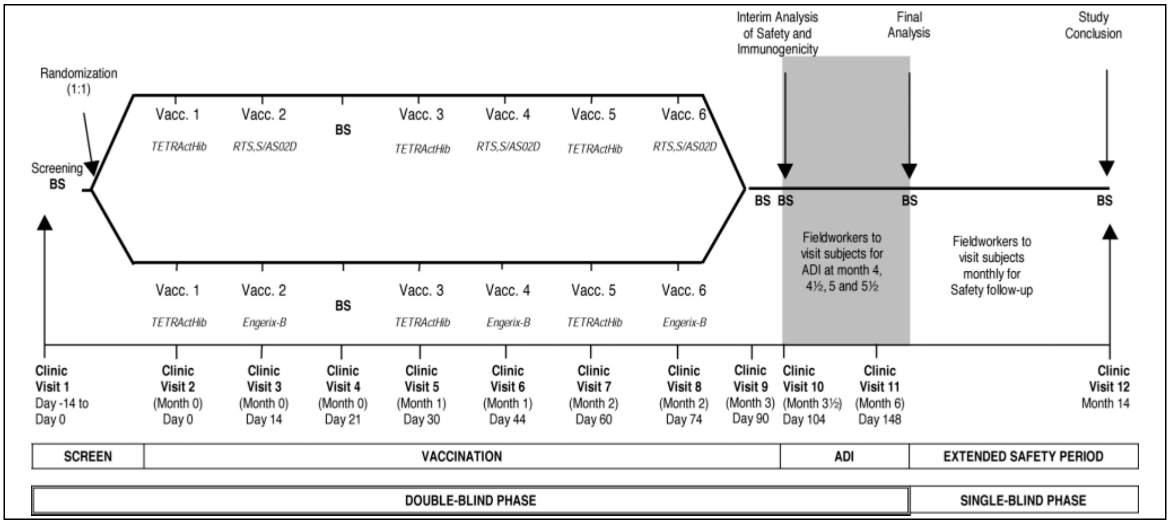

- Phase I/IIb, single-center, double blind (observer blind, participant blind), randomized controlled trial with two groups in one study site.
- Two hundred and twenty children will be enrolled.

Vaccine	Age (weeks)	Number of infants to be enrolled	Estimated number evaluable
TETRActHib	8, 12, 16	110	100
RTS,S/AS02D	10, 14, 18		
TETRActHib	8, 12, 16	110	100
Engerix-B	10, 14, 18		

- Prior to study start, a community information program will inform the local population of the study. Throughout the period of enrollment study information will be presented at antenatal clinics to expectant mothers.
- Infants at high risk of vertical transmission of hepatitis B infection will be excluded from this trial of an experimental hepatitis B vaccine. These infants will receive a licensed hepatitis B vaccine in a schedule beginning at birth. The woman’s full signed informed consent will be required for antenatal HBsAg testing.
- The HIV infection rates of women presenting to antenatal clinics in the Manhiça area is currently approximately 20%. Infants of HIV positive women will be excluded to avoid obscuring the safety pattern associated with the investigational vaccine. The woman’s full signed informed consent will be required for antenatal HIV testing.
- Healthy male and female infants aged 6 to 12 weeks will be screened. Those determined to be eligible, based on the inclusion and exclusion criteria, will be enrolled in the study.
- Infants enrolled for the study attending for the first injection of DTPw will be identified at EPI clinics and, if they meet the inclusion criteria, their parent(s)/guardian(s) asked for consent to the trial.

- Route of administration: all vaccines will be administered by the intramuscular route to the antero-lateral thigh.

  Left thigh: RTS,S/AS02D or Engerix-B

  Right thigh: TETRActHib
- Each infant will be observed for at least 60 minutes after vaccination with RTS,S/AS02D or Engerix-B to evaluate and treat any acute adverse events
- Oral Polio Vaccine will be provided and administered according to the recommended national guidelines (at birth, and coadministered with TETRActHib at 8, 12 and 16 weeks during the study).
- After vaccination, trained field workers will visit the children to detect adverse events (AEs) occurring after vaccination for one week (days 1, 2, 3, 4, 5 and day 6 after each vaccination). Data will be collected after all doses of RTS,S/AS02D or Engerix-B and all doses of TETRActHib. Diary cards and thermometers will be provided for the field workers to record axillary temperature and any local (at the injection site) or general adverse events.
- There will be a 14 day follow-up period after each Dose of TETRActHib and Dose 1 and Dose 2 of RTS,S/AS02D or Engerix-B, and a one month (30 day) follow-up period after Dose 3 of RTS,S/AS02D or Engerix-B for reporting unsolicited symptoms
- Infants will be followed for 14 months after administration of the first dose of TETRActHib.
- Recording of serious adverse events will be throughout the study period. They will be captured through the morbidity surveillance system at Ilha Jossina Health Center***, Taninga Health Center*** and Manhiça District Hospital. In addition all enrolled children will be visited at home monthly by field workers until study conclusion to ensure complete identification of all SAEs ***(Amended 29 June 2005)***.Bloods for safety monitoring of hematology, renal and hepatic function will be measured at 1 week post Dose 1, one month post Dose 3, 3½ months post Dose 3 and 12 months post Dose 3 of RTS,S/AS02D or Engerix-B.
- Non inferiority of hepatitis B response will be determined one month post Dose 3 of RTS,S/AS02D or Engerix-B.
- Anti-CS antibody titers will be determined at baseline, one month post Dose 3, 3½ months post Dose 3 and 12 months post Dose 3 of RTS,S/AS02D or Engerix-B.
- Anti-HBs antibody titers will be determined at baseline, one month post Dose 3 and 12 months post Dose 3 of RTS,S/AS02D or Engerix-B.
- Two weeks prior to Dose 3 of ~~TETRActHib~~ ***RTS,S/AS02D***, children will be treated with sulfadoxine-pyrimethamine and Amodiaquine for presumptive clearance of parasitemia ***(Amended 12 April 2005)***.
- All children will have a blood slide prepared and read to check for asexual *P. falciparum* parasitemia on the day of receiving Dose 3 of RTS,S/AS02D. Any children who test positive will be excluded from ADI.

- The total period of surveillance for ADI is 12 weeks. Seven visits will contribute to the ADI, made up of four field-worker visits and three clinic visits.
- At each contact for ADI, history of fever will be recorded and axillary temperature taken; a blood sample will be taken for malaria parasite genotyping and a smear will be taken for detection of malaria parasites.
- Subjects who are symptomatic at the time of ADI contact (i.e. history of fever within previous 24 hours or axillary temperature >37.5°C) will have a blood slide read and treated within the same day if the blood slide is positive.
- Infants will be followed passively for the occurrence of clinical malaria for the duration of their participation in the study. Cases will be detected at all health facilities in the study area.
- Infection with malaria parasites is defined as *P. falciparum* asexual parasitemia > 0
- Data collection will be by conventional CRF.
- Duration of the study will be 14 months per subject.
- The final analysis of the study for all primary and secondary endpoints will take place on all data collected up to the end of the surveillance for ADI (Clinic Visit 11)
- All children will be followed for safety for a period that extends to 12 months post Dose 3. All data collected after the Final Analysis (Clinic Visit 11) will be reported in an Annex Report.
- This study is overseen by a formally constituted DSMB operating under a charter. During the vaccination phase of the study full safety reports will be sent to the DSMB at 3 points corresponding to; after 30, 60 and 100 subjects have been enrolled and have completed the first 7 days of follow-up post Dose 1 of RTS,S/AS02D or Engerix-B.

4. STUDY COHORT & STUDY SITE

4.1. Study Site

4.1.1. Centro de Investigação em Saude de Manhiça

Centro de Investigação em Saude de Manhiça (CISM) is the first peripheral health research center in Mozambique, founded to undertake medical research into the priority health problems of the Mozambican population and to train Mozambican scientists in research. It was developed under a collaborative program between the Ministry of Health in Mozambique, the Maputo School of Medicine (Universidade Eduardo Mondlane) and the Hospital Clinic in Barcelona (University of Barcelona), with core funding from the Spanish Agency for International Cooperation (AECI). At the center of the study area is Manhiça District Hospital (Health Center of Manhiça). There is close cooperation between CISM and the government services at the district hospital to provide an integrated system of medical research and healthcare provision.

4.1.2. Study site; geography

Centro de Investigação em Saude de Manhiça is located in the District of Manhiça (Maputo Province) in Southern Mozambique approximately 2 hours drive from the capital Maputo. The study area is traversed by the Incomati River, which has wide flood plains that are farmed intensively. The local population lives, in general, on the nearby plateau.

Ilha Josina is located 50 km to the north of Manhiça, at the confluence of two rivers. ***Taninga is located 34 km to the north east of Manhiça, on the banks of the Incomati River (Amended 29 June 2005)***.

There are two distinct seasons. The warm, wet season is between November and April, during which most of the rainfall occurs (annual rainfall for 1998 was 1100 mm and average temperatures of 24°C to 26°C) and a cool, dry season during the rest of the year (average temperatures of 18°C to 23°C).

4.1.3. Study site; malaria transmission

The flood plains of the Incomati River provide ideal breeding conditions for mosquitoes throughout the year. The population living on the flat plateau suffers from the proximity of this area to the mosquito breeding grounds below in the flood plains. Entomological studies have been carried out and the vectors are known, with the most common being *Anopheles funestus* Giles. The Entomological Inoculation Rate (EIR) at Manhiça has been estimated at 38 infective bites/person/year across the study area. With these environmental conditions, malaria in this region is transmitted all year round but is maximal during the months from October to May. This is illustrated in Figure 1.

Figure 1 Malaria rates in children aged 1 to 4 years at Manhiça, Mozambique for the period 1997 to 2001 *

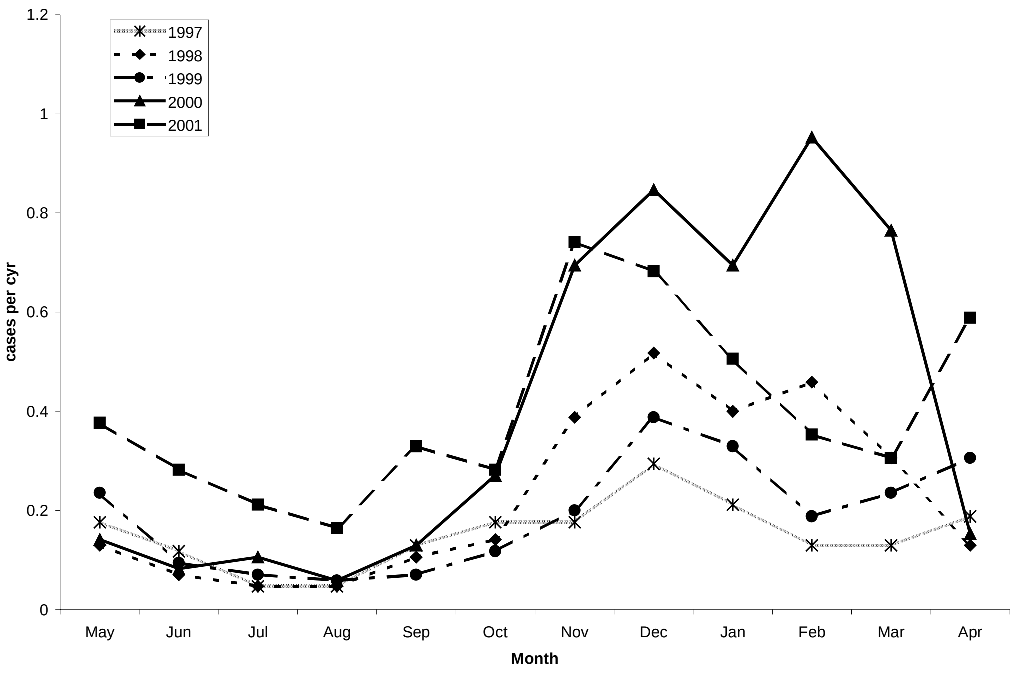

* Defined as the presence of *P. falciparum* asexual parasitemia above 2500 per µl on Giemsa stained thick blood films and the presence of fever [axillary temperature ≥ 37.5°C] at the time of presentation and occurring in a child who is unwell and brought for treatment to a healthcare facility.

The prevalence of parasitemia has been determined by malariometric cross sectional surveys in the Manhiça district. A total of 2057 children were surveyed in one of four surveys, two in the warm, wet seasons (November to April) and two in the cool, dry seasons (May to October) between 1997 and 1999. *Plasmodium falciparum* accounted for 90% of all malaria infections in the children surveyed (the prevalence of asexual *P. falciparum* was 30.5% and 34.0% during the warm seasons and 13.7% and 21.7% during the cool seasons. *P. malariae* was present in each survey with a prevalence of between 1.4% and 2.9%. The prevalence pattern for *P. ovale* was erratic, ranging from 0% to 6.9%).

4.1.4. Study site; population

The population of Manhiça district are primarily Xironga and Xichangana and their languages are usually termed as Ronga and Changana. The two dominant religions are Islam and Christianity. Subsistence and cash crop farming and related industries are the main activities in the area.

Illiteracy is high, affecting 24% of men and 47% of women. While 66% of men and 49% of women have had primary education, only 9% of men and 4% of women have had secondary education and less than 1% of both men and women have gone beyond their secondary education.

Villages in this area typically comprise a loose conglomeration of compounds separated by garden plots and grazing land. Houses are simple, with walls typically made of cane with thatched or corrugated roofs. In towns, houses are often grouped into family compounds and surrounded by grass fences. Towns grew substantially during the civil war in the 1980s as displaced people looked for refuge. Following the end of the civil war in 1992, few inhabitants returned to their original homes and displaced settlements have now been integrated into towns. Water source is mainly from community wells though some households have their own wells. In some areas there are community-sustained pumps. Both wells and pumps are supervised and chlorinated regularly by the District Water and Sanitation Department.

4.1.5. Study site; health care provision in the study area

4.1.5.1. Manhiça District Hospital

The Manhiça District Hospital (Health Center of Manhiça) is a 122-bed health facility that includes a busy outpatient clinic, maternity and child care unit which includes EPI and nutritional services and a 24-hour Emergency Service. It is the closest referral hospital for all the study children.

Patient care at the hospital is currently (January 2004) provided by a staff of 8 medical doctors, 2 medical assistants (Tecnicos de medicina), 15 medical agents (Agentes de medicina), 21 nurses, 11 midwifes, one anesthesiology assistant, two laboratory assistants, one psychiatry assistant and one surgery assistant. Outside of regular working hours, the emergency room is supervised by a medical assistant or doctor and there is always a medical doctor on call to manage emergencies. Oxygen is available for the treatment of patients. There is a basic blood bank on site and blood can be cross-matched and screened for HIV and syphilis. Children participating in this study who require tertiary pediatric care will be referred to the Maputo Central Hospital.

A hospital-based surveillance system was set up at the hospital in 1996, in order to describe the epidemiology and clinical presentation of the important diseases in children living in this rural area of southern Mozambique. Records of all attendances of children up to fifteen years of age are kept, including the diagnosis, laboratory tests and treatments given. Integrating this system into the government hospital service has meant that the CISM has become centrally involved in the day to day running of the hospital, including the support of personnel, diagnostic procedures, equipment and drugs. The strengthening of the hospital in recent years has contributed to the increased confidence of the local population in the local health services and to a very marked and sustained increase in hospital use. In brief, outpatient pediatric attendances have increased from 14 800 in 1997 to 29 800 in 2001. Similarly, the number of pediatric admissions has increased from 971 to 4577 over the same time period.

CISM has also carried out an analysis of the spatial distribution of hospital users in the study area. The increase in the use of the hospital as well as the analysis of CISM data suggest that the hospital is very widely used for important health conditions by the surrounding population and should any SAEs occur, the population will report to the

hospital. Thus, the hospital surveillance system is believed to be a safe and comprehensive method to detect, treat and document SAEs during this study.

4.1.5.2. Ilha Josina Health Center

Ilha Josina Health Center is the closest health center for ~~all~~ ***most*** study children. It does not have inpatient facilities. Currently (October 2004) in addition to the government staff of 1 nurse and 1 midwife it is strengthened by study personnel: 1 Medical Agent (Agente de medicina), 1 microscopist and 2 field workers. Children requiring inpatient management will be transferred to Manhiça District Hospital ***(Amended 29 June 2005)***.

~~The~~ ***Ilha Josina*** Health Center operates during normal office hours, but 24 hour assistance is available at the Health Center. A nurse and midwife live beside Ilha Josina Health Center and are contactable 24 hours a day. If subjects present to ~~the~~ ***Ilha Josina*** Health Center 'out of hours' the nurse or midwife can contact Manhiça District Hospital by telephone or radio and request an ambulance to pick up the patient and transfer them to Manhiça District Hospital ***(Amended 29 June 2005)***.

4.1.5.3. Taninga Health Center

Taninga Health Center does not have inpatient facilities. The quality of care available is equivalent to that available at Ilha Josina Health Center: Currently (June 2005) in addition to the government staff of 1 nurse and 1 midwife it is strengthened by study personnel of 1 midwifery assistant, 1 Medical Agent (Agente de medicina), 1 microscopist and 2 field workers. Children requiring inpatient management will be transferred to Manhiça District Hospital.

Taninga Health Center operates during normal office hours, but 24 hour assistance is available at the Health Center. A nurse and midwife live beside Taninga Health Center and are contactable 24 hours a day. If subjects present to Taninga Health Center 'out of hours' the nurse or midwife can contact Manhiça District Hospital by telephone or radio and request an ambulance to pick up the patient and transfer them to Manhiça District Hospital (Amended 29 June 2005).

4.1.5.4. Malaria control

The Ministry of Health malaria control program has a strategy of reduction of transmission and effective case management. It is recommended that all children and pregnant women should sleep under an impregnated bednet. Bednets are available in the study area through a social marketing program. CISM are working alongside the Ministry of Health to support this program.. First line therapy for malaria disease is sulfadoxine-pyrimethamine and amodiaquine. This regimen was recently introduced by the Mozambican Ministry of Health and has a high level of efficacy; sulfadoxine-pyrimethamine clinical response of 83% and amodiaquine of 93% (personal communication, Dr Pedro Alonso, 2002).

4.1.5.5. EPI services

The Expanded Program on Immunization (EPI) provides vaccination free of charge to the population. In the study area, clinics are operated every weekday at the Ilha Josina Health Center ***and at the Taninga Health Center (Amended 29 June 2005)***. The infant immunization schedule in Mozambique is:

BCG	Birth
D, T, Pw, hepatitis B	2, 3, 4 months of age
Measles	9 months of age
OPV	Birth; 2, 3, 4 months of age

Vaccination against *Hemophilus influenzae* type B is not currently included in the Mozambican EPI schedule.

4.1.5.6. HIV services

The Ministry of Health has recently introduced new recommendations for HIV management based on a system of voluntary testing and combination anti-retroviral treatment for the immunosuppressed adults and children. This new management is being rolled-out across Mozambique and will be available in full in the study area by the start of the trial. Vertical transmission is addressed by a policy of offering HIV screening to women attending antenatal clinics. CISM is coordinating with the Mozambican government to ensure that these services are rolled out and available by the start of the study.

4.1.5.6.1. HIV management; Adults

Ministry of Health policy is to commence multi-drug antiretroviral therapy in adults in whom there is clinical or laboratory evidence of immunosuppression. The first line therapy is Stavudine plus Lamidovudine with Nevirapine for all adults.

4.1.5.6.2. HIV management; Children

Ministry of Health policy is to commence multi-drug antiretroviral therapy in children in whom there is clinical or laboratory evidence of immunosuppression. These children should receive therapy based on a protease inhibitor (Indinavir, Nelfinavir or Ritonavir) together with two nucleoside inhibitors of reverse transcriptase (Estavudine, Didanosine, Lamivudine, Zalcitabine or Zidovodine) or one non nucleoside reverse transcriptase inhibitor (Nevirapine or Efivarenz) with two nucleoside reverse transcriptase inhibitors.

4.1.5.6.3. HIV management; Prevention of vertical transmission

The Ministry of Health has a policy to reduce vertical transmission of HIV; all women attending antenatal clinics are offered screening for HIV infection by trained counselors. Current recommendations for symptomatic women are that they are treated as per Section 4.1.5.5.1. Current recommendations are for asymptomatic women to receive treatment

with Combivir (Zidovudine plus Lamidovudine) from 32 weeks of pregnancy, followed in labor by Nevirapine, and after delivery with Zidovudine and Lamidovudine for three days. All infants should receive Nevirapine 72 hours after birth.

4.2. Number of subjects

Infants will be recruited by non-coercive methods according to existing policies of the CISM (see Appendix C). Eligible subjects will be male and female infants born to women who have previously been screened for current infection with hepatitis B virus or HIV as described under Section 5.1.10.2. See Section 10.5 for a detailed description of the criteria used in the estimation of sample size.

The necessary number of children will be screened in order to enroll approximately 220 eligible subjects, 110 to each group.

4.3. Inclusion criteria

All subjects must satisfy the following criteria at study entry:

- A male or female infant of between 6 and 12 weeks of age at the time of first vaccination.
- Written informed consent obtained from the parent(s) or guardian(s) of the subject
- Free of obvious health problems as established by medical history and clinical examination before entering into the study.
- Born to a mother who is HBsAg negative
- Born to a mother who is HIV negative.
- Born after a normal gestation period (between 36 and 42 weeks).
- Subjects who the investigator believes that their parents/guardians can and will comply with the requirements of the protocol should be enrolled in the study.

4.4. Exclusion criteria for enrollment

The following criteria should be checked at the time of study entry. If any apply, the subject must not be included in the study:

- BCG administration within one week of proposed administration of a study vaccine.
- Use of any investigational or non-registered drug or vaccine other than the study vaccines within 30 days preceding the first dose of study vaccine, or planned use during the study period.
- Administration of immunoglobulins and/or any blood products since birth or planned administration during the study period.
- Chronic administration (defined as more than 14 days) of immunosuppressants or other immune-modifying drugs since birth (for corticosteroids, this will mean prednisone, or equivalent, ≥ 0.5 mg/kg/day. Inhaled and topical steroids are allowed).

- Any chronic drug therapy to be continued during the study period.
- Previous vaccination with diphtheria, tetanus, pertussis (whole-cell or acellular), *Hemophilus influenzae* type b or hepatitis B vaccines.
- Major congenital abnormality.
- Serious acute or chronic illness determined by clinical, physical examination and laboratory screening tests
- Any medically diagnosed or suspected immunodeficient condition based on medical history and physical examination (no laboratory testing required)
- A family history of congenital or hereditary immunodeficiency.
- History of allergic disease or reactions likely to be exacerbated by any component of the vaccine.
- History of any neurological disorders or seizures.
- Maternal death.
- Moderate malnutrition at screening defined as weight for age Z-score less than -2; this corresponds to a weight of ≤ 3.9 kg for 2 month old boys and ≤ 3.6 kg for 2 month old girls ***(Amended 17 January 2005)***.
- Hemoglobin < 80 g/L
- Simultaneous participation in any other clinical trial.
- Same sex twin
- Any other findings that the investigator feels would increase the risk of having an adverse outcome from participation in the trial.

4.5. Elimination criteria during the study

The following criteria should be checked at each visit subsequent to the first visit. If any become applicable during the study, it will not require withdrawal of the subject from the study but may determine a subject's evaluability in the according-to-protocol (ATP) analysis. See Section 10.4 for definition of study cohorts to be evaluated.

- Use of any investigational or non-registered drug or vaccine other than the study vaccine(s) during the study period.
- Chronic administration (defined as more than 14 days) of immunosuppressants or other immune-modifying drugs during the study period (for corticosteroids, this will mean prednisone, or equivalent, ≥ 0.5 mg/kg/day. Inhaled and topical steroids are allowed).
- Administration of a vaccine not foreseen by the study protocol during the period starting from 30 days before each dose of vaccine(s) and ending 30 days after.
- Administration of immunoglobulins and/or any blood products during the study period.
- Administration of either TETRActHib or study vaccines in the incorrect limb.

- ***Failure to thrive***

(Amended 12 April 2005).

4.6. Contraindications to any vaccination dose

The following AEs constitute contraindications to administration of RTS,S/AS02D at that point in time; if any one of these AEs occurs at the time scheduled for vaccination, the subject may be vaccinated at a later date, within the time window specified in the protocol (see Section 5.3), or withdrawn at the discretion of the investigator (see Section 9). The subject must be followed until resolution of the event, as with any AE (see Section 8.6).

- Acute disease at the scheduled time of vaccination. Acute disease is defined as the presence of any moderate or severe illness, or a mild illness with axillary temperature ≥ 37.5°C (all vaccines can be administered to persons with a minor illness such as diarrhea, mild upper respiratory infection with or without low-grade febrile illness).
- Axillary temperature ≥ 37.5°C.
- A lesion that would prevent intramuscular injection in the left or right antero-lateral thighs

4.7. Contraindications to subsequent vaccination dose

The following adverse events (AEs) constitute absolute contraindications to further administration of RTS,S/AS02D. If any of these AEs occur during the study, the subject must not receive additional doses of vaccine but may continue other study procedures at the discretion of the investigator (see Section 9). The subject must be followed until resolution of the event, as with any AE (see Section 8.6):

- Anaphylactic reaction following the administration of vaccine(s).
- Any confirmed or suspected immunosuppressive or immunodeficient condition, including human immunodeficiency virus (HIV) infection.

See Section 5.1.7 for a description of how these children will receive further vaccinations with licensed vaccines.

5. CONDUCT OF STUDY

5.1. Ethics and regulatory considerations

The study will be conducted according to Good Clinical Practice (GCP), the appended Declaration of Helsinki (Protocol Appendix A), and local rules and regulations of the country.

The following two sections provide guidance to the study investigator of the minimal ethical and regulatory requirements required by GSK but the final responsibility for the interactions between the Institutional Review Boards (IRBs) and Independent Ethics

Committees (IECs) and for informed consent remain with the Principal Investigator and may be subject to local rules and regulations.

5.1.1. Institutional Review Board/Independent Ethics Committee (IRB/IEC)

The IRB/IEC must be constituted according to the local laws/customs of each participating country. The ICH Harmonized Tripartite Guideline for Good Clinical Practice recommends that the IRB/IEC should include:

(a) At least five members.

(b) At least one member whose primary area of interest is in a non-scientific area.

(c) At least one member who is independent of the institution/study site.

Only those IRB/IEC members who are independent of the investigator and the sponsor of the study should vote/provide opinion on a study-related matter.

A list of the professions of the IRB/IEC members should be obtained by the principal investigator

This protocol and any other documents that the IRB/IEC may need to fulfill its responsibilities, including subject recruitment procedures and information about payments and compensation available to subjects, will be submitted to the IRB/IEC by the principal investigator. Written unconditional approval of the IRB/IEC must be in the possession of the investigator and GSK Biologicals before commencement of the study. This approval must refer to the study by exact protocol title and number, and should identify the documents reviewed and state the date of review. Relevant GSK Biologicals' data will be supplied by the principal investigator to the independent IRB/IEC for review and approval of the protocol. Verification of IRB/IEC unconditional approval of the protocol and the written informed consent statement will be transmitted by the principal investigator to GSK Biologicals' Study Monitor, using the standard notification form, prior to shipment of vaccine supplies and CRFs to the site.

No deviations from, or changes to, the protocol should be initiated without prior written consent of the sponsor and IRB/IEC favorable opinion of an appropriate amendment except when necessary to eliminate immediate hazards to the subjects or when the change(s) involves only logistical or administrative aspects of the study (e.g., change of monitor[s], telephone number[s]). Modifications are submitted to the IRB/IEC for information only. However, written verification that the modification was submitted should be obtained. Approvals/verifications must be transmitted in writing to GSK Biologicals' Study Monitor by the principal investigator.

The IRB/IEC must be informed by GSK Biologicals' Study Monitor, of:

- all subsequent protocol amendments, informed consent changes or revisions of other documents originally submitted for review;

- serious and/or unexpected adverse events occurring during the study, where required,
- all subsequent protocol modifications (for information);
- new information that may affect adversely the safety of the subjects or the conduct of the study;
- an annual update and/or request for re-approval, where required;
- when the study has been completed, where required.

If a trial is prematurely terminated or suspended for reasons including, but not limited to, safety or ethical issues or severe non-compliance, the sponsor will promptly inform the regulatory authorities of the termination or suspension and the reason(s) for the termination or suspension. If required by applicable regulations, the investigator must inform the IEC/IRB promptly and provide the reason for the suspension or termination (see Section 5.1.3 and Appendix B for further details).

5.1.2. Informed consent

The details of the informed consent process are provided in Appendix C. The following principles will also apply.

In obtaining and documenting informed consent, the investigator should comply with the applicable regulatory requirement(s), and should adhere to GCP and to the ethical principles that have their origin in the appended Declaration of Helsinki. Prior to the beginning of the trial, the investigator should have the IRB/IEC's written approval/favorable opinion of the written informed consent form and any other written information to be provided to the subjects' parents/guardians.

Information should be given in both oral and written form whenever possible and as deemed appropriate by the IRB/IEC.

An investigator or designate will describe the protocol to potential subjects' parents/guardians face to face. The Subject Information and Consent Form may be read to the subjects' parents/guardians, but, in any event, the investigator or designate shall give the subjects' parents/guardians ample opportunity to inquire about details of the study and ask any questions before dating and signing the Consent Form.

Subject information and consent forms must be in a language fully comprehensible to the prospective subjects' parents/guardians. Informed consent shall be documented by the use of a written consent form approved by the IRB/IEC and signed and dated by the subjects' parents/guardians and by the person who conducted the informed consent discussion. The signature confirms the consent is based on information that has been understood. All illiterate individuals will have the study, the Subject Information and Consent Form explained to them point by point by the interviewer in the presence of an impartial witness. The subjects' parents/guardians will thumbprint or sign the consent form. The witness will also sign and date the consent form. Oral witnessed consent will replace written consent only in countries where the local custom is contrary or if the subjects' parents'/guardians' incapacity precludes this and provided that the local legal obligations are fulfilled.

Each subject's signed informed consent form must be kept on file by the investigator for possible inspection by Regulatory Authorities and/or GSK Biologicals' professional and Regulatory Compliance persons. The subjects' parents/guardians should receive a copy of the signed and dated written informed consent form and any other written information provided to the subjects' parents/guardians and should receive copies of any signed and dated consent form updates. Any amendments to the written information will be provided to subjects' parents/guardians.

Both the informed consent discussion and the written informed consent form and any other written information to be provided to the subjects' parents/guardians should include explanations of the following:

(a) That the trial involves research.

(b) The purpose of the trial.

(c) The trial treatment(s) and the probability for random assignment to each treatment.

(d) The trial procedures to be followed, including all invasive procedures.

(e) The subject's parents'/guardians' responsibilities.

(f) Those aspects of the trial that are experimental.

(g) The reasonably foreseeable risks or inconveniences to the subjects and, when applicable, to an embryo, fetus or nursing infant.

(h) The reasonable expected benefits. When there is no intended clinical benefit to subjects, the subjects' parents/guardians should be made aware of this.

(i) The alternative procedure(s) or course(s) of treatment/methods of prevention that may be available to subjects, and their important potential benefits and risks.

(j) The compensation and/or treatment available to subjects in the event of trial-related injury.

(k) The anticipated prorated payment, if any, to subjects' parents/guardians for participating in the trial.

(l) The anticipated expenses, if any, to subjects' parents/guardians for participating in the trial.

(m) That the subjects' participation in the trial is voluntary and subjects' parents/guardians may refuse to participate or withdraw from the trial, at any time, without penalty or loss of benefits to which subjects are otherwise entitled.

(n) That the monitor(s), the auditor(s), the IRB/IEC, and the regulatory authority(ies) will be granted direct access to the subject's original medical records for verification of clinical trial procedures and/or data, without violating the confidentiality of subjects, to

the extent permitted by the applicable laws and regulations and that, by signing a written informed consent, the subject's parents/guardians is authorizing such access.

(o) That records identifying subjects will be kept confidential and, to the extent permitted by the applicable laws and/or regulations, will not be made publicly available. If the results of the trial are published, subjects' identity will remain confidential.

(p) That the subjects' parents/guardians will be informed in a timely manner if information becomes available that may be relevant to the subjects' parents/guardians willingness for continued participation in the trial.

(q) The person(s) to contact for further information regarding the trial and the rights of trial subjects, and who to contact in the event of trial-related injury.

(r) The foreseeable circumstances and/or reasons under which a subject's participation in the trial may be terminated.

(s) The expected duration of a subject's participation in the trial.

(t) The approximate number of subjects involved in the trial.

GSK Biologicals will prepare a model Informed Consent Form which will embody all the elements described above. While it is strongly recommended that this model document be followed as closely as possible, the informed consent requirements given in this document are not intended to pre-empt any local regulations which require additional information to be disclosed for informed consent to be legally effective. Clinical judgement, local regulations and requirements should guide the final structure and content of the document.

The investigator has the final responsibility for the final presentation of Informed Consent Form, respecting the mandatory requirements of local regulations. The consent form generated by the investigator with the assistance of the sponsor's representative, must be approved (along with the protocol, and any other necessary documentation) by the IRB/IEC and be acceptable to GSK Biologicals.

5.1.3. Data Safety Monitoring Board (DSMB)

An independent committee consisting of experts in malaria, pediatrics, statistics and other appropriate disciplines has been appointed to oversee ethical and safety aspects of the study conduct. A quorum of 3 members is required at scheduled meetings.

The role of the DSMB includes the review of the implementation and progress of the study. It provides initial, regular, and closing advice on safety-related issues to GSK Biologicals. Its advice is based on the interpretation of study data with reference to the study protocol.

The DSMB will confer before the initiation of the study (pre-initiation review), during the study at points of safety review and at the close of the study. They will review the Protocol, Report and Analysis Plan (RAP) and Study Report. Other unscheduled

meetings may be required. Meetings must be documented and minutes made available to the sponsors. The DSMB may, if deemed necessary, convene a meeting with, or request further information from the Principal Investigators, the Medical Monitor/Local Safety Monitor and GSK Biologicals' and MVI at PATH's designated project representatives at any stage of the study.

The DSMB is empowered to suspend the enrollment to the trial and/or vaccination on the trial pending review of potential safety issues; complete details of this process are given in Section 5.1.5.

5.1.3.1. Data Reviewed by the DSMB

The DSMB must be informed by the Local Safety Monitor (LSM) of the following safety data on an 'as received' basis:

- All SAEs;
- All withdrawals of study subjects by the Principal Investigator or the parent(s)/guardian(s) of a subject due to adverse events.

The DSMB will receive from the sponsor, GSK Biologicals:

- Safety summary reports at 3 predefined timepoints; after 30, 60 and 100 subjects have been enrolled and have completed the first 7 days of follow-up post Dose 1 of RTS,S/AS02D or Engerix-B (refer to Section 5.1.5 for the contents of the summary reports).
- New information that may affect adversely the safety of the subjects or the conduct of the study;
- All subsequent protocol amendments, informed consent changes or revisions of other documents originally submitted for review;
- All subsequent protocol modifications (for information).

5.1.4. Local Safety Monitor (LSM)

The overall role of the Local Safety Monitor (LSM), who is an experienced clinician based in-country, will be to support the clinical investigators and to act as a link between the investigators and the DSMB.

The LSM's role will include:

- Acting as the study volunteer's advocate;
- Promptly communicating relevant safety information to the DSMB;
- Providing advice to the investigators on whether a set of clinical circumstances in a study warrants formal notification to the DSMB;
- Unblinding a subject if deemed necessary to allow for adequate treatment;
- Liaising closely with the chair of the DSMB throughout the course of the trial;

- Suspension of vaccination for a major safety concern pending discussion with the DSMB (see Section 5.1.5 for full details).

5.1.4.1. Data Reviewed by the LSM

The LSM must be informed by the investigator on an 'as received' basis of:

- All SAEs;
- All withdrawals of study subjects by the Principal Investigator or the parent(s)/ guardian(s) of a subject due to adverse events.

5.1.5. Safety monitoring plan

Safety reports will be produced for the DSMB on all data collected until 3 points in the study; after 30, 60 and 100 subjects have been enrolled and have completed the first 7 days of follow-up post Dose 1 of RTS,S/AS02D or Engerix-B. An independent statistician will analyze the data, thereby maintaining the blind of the malaria project team at GSK Biologicals and the investigator group. If a criteria for the suspension of enrolment or vaccination (refer to Section 5.1.6.2 and Section 5.1.6.1 respectively) is met, or the DSMB have any safety concerns about the vaccines, they may suspend the ongoing enrollment and vaccination in the trial. The process outlined in Section 5.1.6 will be followed.

The reports will contain analyses by group:

- All SAEs and any relationship to vaccine (RTS,S/AS02D, Engerix-B or TETRActHib) to date
- For each dose of RTS,S/AS02D or Engerix-B and TETRActHib, all solicited AEs tabulated by severity grading (any and Grade 3 alone) and relationship to vaccine.
  - Should the investigator judge a case of Grade 3 fever to be unrelated to vaccination, an alternative explanation for the cause of the fever will be provided
- For each dose of RTS,S/AS02D or Engerix-B and TETRActHib, all unsolicited AEs tabulated by severity grading (any and grade 3 alone) and relationship
- ~~All laboratory values up to 30 days post Dose 3 presented as number of subjects out of range (above and below normal range) at each sampling time point (post doses 1 and 3 of RTS,S/AS02D or Engerix-B)~~
- ***Laboratory values are scheduled to be collected at 1 week post Dose 1 of RTS,S/AS02D, 1 month post Dose 3 of RTS,S/AS02D, 5½ months post Dose 3 of RTS,S/AS02D and at the end of the single-blind phase of the study (Month 14). All available laboratory values up until the data base lock point of each of the three scheduled safety reports will be provided (Amended 29 June 2005)***
  - For all subjects out of range clinical details will be provided
- All withdrawals of study subjects by the Principal Investigator due to adverse events recorded from the infants or withdrawals of infants by the parent(s)/guardian(s)

following doses of RTS,S/AS02D or Engerix-B and TETRActHib (expressed as percentage of subjects enrolled).

5.1.6. Process for the suspension of vaccination

5.1.6.1. Indications for immediate suspension of all vaccination

Immediate suspension of all vaccination on the trial will take place if:

- The Principal Investigator suspends vaccination for any of the following SAEs pending review by the DSMB;
  - Death or life-threatening SAE which is judged to be related to the study vaccine;
  - Anaphylactic shock reaction in an enrolled subject following vaccination.
- The DSMB recommend suspension of all vaccination for any one SAE or pattern of SAEs. The DSMB will communicate their recommendation to the Principal Investigator who will enact it. The DSMB will notify the sponsors of their decision immediately;
- The DSMB recommend suspension of all vaccination following their review of the safety summary reports

5.1.6.2. Indications for suspension of enrolment to the trial

Enrolment to the trial will be suspended if:

- The DSMB review of a safety report shows ≥ 5% of subjects vaccinated with RTS,S/AS02D are withdrawn by the investigator for local or systemic reactogenicity in recipients of RTS,S/AS02D. In making their recommendation the DSMB will take into account the full clinical history of each withdrawn child.
- The DSMB review of a safety report shows ≥ 5% of doses followed by fever > 39.0°C judged to be related to vaccination in recipients of RTS,S/AS02D. In making their recommendation the DSMB will review the investigators assessment of relatedness of all Grade 3 fevers.
- The DSMB recommend suspension of enrollment following their review of a safety summary reports.

5.1.6.3. Process if the trial is suspended

Although the trial may be suspended by the DSMB, the LSM or the Principal Investigator, it is the responsibility of the sponsor (GSK Biologicals) to make the recommendation whether or not the trial should be stopped permanently.

If the trial is suspended, the DSMB will review all available information and make a recommendation to the study sponsor (GSK Biologicals) whether to recommence the trial or to stop the trial permanently. In the event that the DSMB recommend to stop the trial permanently, the FDA will be informed by GSK Biologicals that the trial is suspended.

In the event that the trial is suspended on the recommendation of the DSMB the sponsor (GSK Biologicals) will evaluate the information. If the sponsor concurs with the DSMB's recommendation to suspend the trial, GSK Biologicals will inform the FDA that the trial has been stopped ~~permanently~~ ***temporarily***. If the sponsor's recommendation is to continue, then a report will be submitted to the FDA detailing the rationale used in reaching this decision. The agreement of the FDA will be obtained prior to restarting the trial ***(Amended 29 June 2005)***.

5.1.7. Completion of immunization course for infants withdrawn during vaccination course

Infants may be withdrawn by the investigator for medical reasons or by their parent(s)/guardian(s) for any reason. If there is no contraindication, in the opinion of the Principal Investigator, for the infant receiving additional doses of licensed vaccine for D, T, Pw, Hib and hepatitis B, the importance of completion of a 3 dose regimen will be explained to the parent(s)/guardian(s). The parent(s)/guardian(s) will be offered the choice of completing a course of TETRActHib + Engerix-B administered by the study team or completing a course of D, T, Pw, hepatitis B at their local EPI clinic.

5.1.8. Rescue plan for Hepatitis B response

Antibody response to HBsAg will be determined 30 days after Dose 3 of RTS,S/AS02D or Engerix-B in infants. Infants who have not attained seroprotective levels of anti-HBs antibodies will be investigated further and their HBsAg measured to determine whether or not they are chronic carriers of hepatitis B virus. Chronic carriers are known to respond poorly to licensed hepatitis B vaccines and therefore may not benefit from additional doses of hepatitis B vaccine. Children who are not chronic carriers will be offered additional doses of the licensed vaccine. Children who are withdrawn, or drop out prior to the Day 104 will be invited to the Day 104 clinic visit (Clinic Visit 10) for safety monitoring and to assess their hepatitis B seroprotection status.

5.1.9. Rescue plan for D, T, Pw, and Hib responses

The EPI vaccines are not coadministered with RTS,S/AS02D in this trial, but the third dose of TETRActHib will coincide with the administration of sulfadoxine-pyrimethamine (SP)and Amodiaquine for parasite clearance. Experience of the safety and immunogenicity of these drugs coadministered with EPI vaccines has been gathered in the evaluation of the malaria control strategy, the intermittent preventive treatment of infants IPTi [Schellenberg, 2001]. In this study, all infants will be monitored for their response to D, T, Pw and Hib and receive booster doses where required.

5.1.10. Recruitment

5.1.10.1. Community information

The community in which the study will take place will be informed about the nature and design of the study. Refer to Appendix C for an overview of the recruitment plan of the study.

5.1.10.2. Antenatal visits

Study personnel will attend the antenatal clinics in the study area. They will be available to answer any questions women may have concerning the vaccine trial. Women in the third trimester of pregnancy will be asked to consider enrolling their child in the vaccine trial. The trial will be explained and the SIS given for them to take home.

As a trial procedure, women in the third trimester of pregnancy who are considering enrolling their infant in the study will be screened for HBsAg and HIV. It is a part of routine antenatal care provided by the Government Health Services to screen for HIV infection in pregnancy, but not for chronic carriage of hepatitis B virus. Written informed consent will be requested in private and if given, blood taken for HBsAg and HIV testing. Full confidentiality of test results will be maintained. Test results will not be recorded in the CRF. Trained counselors will deliver test results to women. Infants born to mothers who test positive for HBsAg or HIV will not be eligible to join the trial

5.1.10.2.1. Management of infants born to HBsAg positive mothers

It is not current medical practice in Mozambique to screen women in pregnancy for the detection of the chronic carrier state of hepatitis B. All infants routinely receive immunization with a hepatitis B vaccine at 8, 12, 16 weeks of age.

A direct benefit of knowing HBsAg serostatus is that if a woman is found to be HBsAg positive, the immunization of the infant against hepatitis B can begin at birth. The study team will ensure that infants born to mothers found to be chronic carriers of hepatitis B are offered a hepatitis B immunization schedule beginning at birth.

5.1.10.2.2. Management of HIV positive mothers and their infants

Any woman who is found to be HIV positive will be informed of her test result by a trained counselor. The study clinician will see the woman and explain why the newborn baby would not be eligible to enroll in the trial. The clinician will refer the woman to the government health care services for medical management. The Ministry of Health recommendations for management of HIV infection are given in Section 4.1.5.6.1 The close cooperation between Centro de Investigação em Saude de Manhiça and the government services at the District Hospital ensures that an integrated system of healthcare is provided (refer to Section 4.1.5 for an overview of health care provision in the study area).

5.1.10.3. Screening of infants

Only infants whose mothers have previously given consent, been tested and found to be negative for carriage of the hepatitis B virus and HIV infection will be eligible to be screened for the trial. Infants attending for the first injection of DTPw will be identified at EPI clinics and their parent(s) or guardian(s) asked for consent to the trial following ICH guidelines (refer to Section 5.1.2 and Appendix C). Comprehension of the information contained within the Informed Consent form will be checked prior to screening by an oral interview with the subject's parent(s)/guardian(s), according to SOPs at CISM. If consent is not available from both parents or guardians, the reason for the unavailability of one of the parents or guardians will be specified on the consent form. When the parent(s)/guardian(s) of the infant are illiterate, the informed consent will also be signed and dated by a witness.

Only infants with a written Informed Consent form, signed/thumb-printed and dated by parents/guardians will be screened. Subject numbers will be allocated to all infants who are consented for screening by their parent(s)/guardian(s). Subject numbers will be issued consecutively. Once consent is obtained, then per-protocol eligibility criteria will be checked, which will necessitate a physical examination and blood sampling for assessment of hematology, renal and liver function. This will be documented on clinic forms, which are prepared and filled in by the investigator. Each form will contain the subject number, permanent study area ID number of the subject (an identifier in the CISM demographic database), information about the subject's date of birth, household, date of screening visit, medical history, physical and laboratory screening examination. After reviewing the medical history, physical examination and laboratory results, any reasons for non-eligibility will be documented on the screening list.

The parent(s)/guardian(s) of children who have been consented for the study and found to be ineligible will receive a full explanation by a medical doctor and appropriate medical management. The screening lists for all children screened for entry to this study will be kept in a secure, locked area at the investigator's site. They will only be accessible to authorized persons. Any clinically relevant finding will be treated appropriately by a physician. An overview of the laboratory assays can be found in Appendix D and further details can be found in CISM SOPs.

A study identification card will be prepared for the parent(s)/guardian(s) of each screened subject. At screening a photograph of each screened subject being held by their parent(s)/guardian(s) will be taken and attached to the study identification card. This card will also bear the name of the study to which the child is enrolled, the child's subject number and permanent study area ID.

5.1.11. Vaccination process

The vaccines RTS,S/AS02D and Engerix-B will be packaged in identical boxes and will be identified by a treatment number. The unique treatment number will identify all doses of these vaccines administered to each subject. After randomization the treatment number assigns the subject to one group or another in a blinded way. The administration of the vaccine TETRActHib is not blinded; however it will be identified by a treatment number.

The treatment number will be issued sequentially at Clinic Visit 2. Each subject will retain the same treatment number for their subsequent vaccine doses. The treatment number will be recorded on the subject's Clinic Form after the vaccine has been administered; information from the Clinic Form is subsequently transferred to the Case Report Form. The Clinic Form and Case Report Form link the subject number and the treatment number.

- All vaccines will be administered by the intramuscular route to the antero-lateral thigh: left thigh RTS,S/AS02D or Engerix-B and right thigh TETRActHib. Subjects who receive their vaccination with in the incorrect leg will continue to receive subsequent blinded vaccinations of RTS,S/AS02D or Engerix-B and unblinded vaccinations with TETRActHib as normal. The fact that the vaccine was administered in the wrong limb will be documented and they will be eliminated from the According to Protocol Cohort for Safety.

Vaccinations will take place at the EPI clinic of Ilha Josina Health Center ***and Taninga Health Center (Amended 29 June 2005)***. All vaccinations will be given by a qualified person; a nurse or a doctor or a 'medical agent of preventative medicine'. A staff member experienced in the resuscitation of children will be available at all vaccination sessions. Facilities and equipment will be available to give emergency treatment in the case of an anaphylactic reaction following administration of vaccines. All children will be observed for an hour after the administration of vaccine to evaluate and treat any acute adverse events.

The process for each vaccination is as follows. The identity of the child will be confirmed using the study identification card. The subject number on this card will be cross-checked with that on the subject's clinic form, ensuring that the subject number on the clinic form matches that of the study identification card. On the day of the first vaccination the inclusion/exclusion criteria and consent form will be checked prior to vaccination. On the days of the subsequent vaccinations, elimination criteria and contraindications to vaccination will be checked prior to vaccination. A vaccine clinic form will be initiated, which will give the child's identifiers, subject number and treatment number assigned after the administration of the vaccine.

For each vaccination during the course of the study, the Vaccinator will prepare and administer the vaccine for a specific subject. Since the vaccines used in this study are of distinct appearance, the Vaccinators are not blinded and perform no other function in the study (refer to Section 6.5). The Vaccinator will select the sealed box labeled with the subject's treatment number, containing the vials numbered with the treatment number, remove the vaccine vials and fill a syringe according to this study protocol (refer to Section 6.2). The Vaccinator will then place a numbered opaque label with the subject's treatment number on the syringe. The purpose of masking the syringe is to blind the parent(s) or guardians(s) of the subject. After administering the vaccination to the subject the Vaccinator will enter the treatment number administered to the subject on the clinic form.

Subjects who cannot be vaccinated on the originally scheduled date (due to an acute illness on the judgment of the PI or designate, local lesion at the injection site or scheduling conflicts) will be vaccinated within 7 days and undergo all study procedures

for the visit on the same day as vaccination. In the particular case of any child found to be febrile (axillary temperature ≥ 37.5°C), a blood slide will be taken to investigate for malaria. Children will be treated as appropriate for their condition and will be followed up until resolution of any symptoms and be vaccinated if their clinical symptoms resolve within 7 days.

Those who cannot be re-vaccinated within 7 days of their scheduled date will continue all study procedures apart from receiving further study vaccinations.

The parent(s)/guardian(s) of infants who are withdrawn from the study will be advised on an appropriate method of completing the infants vaccination regimen (refer to Section 5.1.7).

5.1.12. Home follow-up visits for assessment of reactogenicity (7-day follow-up period)

At Dose 1 of RTS,S/AS02D or Engerix-B, trained field workers under the supervision of the Principal Investigator will visit each enrolled child at daily intervals for Days 1 to 5 post Dose 1 of RTS,S/AS02D or Engerix-B; a study clinician will examine the infant on Day 6. For all other doses, the field workers will visit each enrolled subject at daily intervals for Days 1 to 6 post each vaccination (see below in detailed study procedure; Section 5.4). In the event that the field worker finds any Grade 3 solicited general or unsolicited symptoms, the volunteer will be brought to CISM for examination by a study clinician. Any further clinical data, including treatment provided, will be written on diary cards and CISM clinic forms and transcribed onto the CRF. If the physician finds that the volunteer has experienced an SAE the appropriate measures will be taken to report this (See Section 8.7).

Diary cards will be checked and verified by the Principal Investigator or designate before transcription onto CRFs after the 7-day follow-up. The Principal Investigator has a primary responsibility for the data transcribed onto the CRFs. Unresolved AEs will be followed-up by field workers until resolution under the supervision of the Principal Investigator and data will be entered onto the CRF. The procedures and frequency of visits will be outlined in a SOP at the investigator’s site.

Analgesics/antipyretics will be provided to field workers for the treatment of children with injection site pain and fever and their use will be documented. Parent(s)/guardian(s) will not routinely be provided with these medications.

5.1.13. Monitoring of hematological and biochemical laboratory parameters

The hematological and biochemical parameters will be documented on the CRF. For all values outside the normal range, the reason and/or clinical condition will be documented. Results of hematological and biochemical laboratory tests will be reviewed as soon as they are generated. Any value outside the normal range will be managed as appropriate by a medically qualified individual under the supervision of the Principal Investigator. Guidance on when to report abnormalities as SAEs is given in Section 8.3.

5.1.14. Surveillance for SAEs (all subjects)

5.1.14.1. At health facilities

Morbidity surveillance has been in place at Manhiça District Hospital since 1996 and in Ilha Josina ***and Taninga*** since 2001 (a detailed review of the level of health care service is given in Section 4.1.5). These ~~two~~ ***three*** health facilities are the source of primary care for this area. The surveillance system will provide a comprehensive recording of all outpatient attendances, the investigational results, diagnosis and management. Prior to the start of the study, all parent(s)/guardian(s) of study children will be educated on the appropriate action they should take if their child becomes unwell at any time during the study period. They will be asked not to medicate their child at home, but to seek medical care at ~~either~~ Ilha Josina Health Center***, Taninga Health Center*** or Manhiça District Hospital ***(Amended 29 June 2005)***.

Study staff (a nurse, medical agent or field worker) will be available 24 hours per day at Manhiça District Hospital to receive and identify study participants when they present and to ensure complete investigation and documentation of the attendance. At Ilha Josina Health Center ***and Taninga Health Center*** staff are available 24 hours a day to arrange the transport of study participants to Manhiça District Hospital (refer to Section 4.1.5.2) ***(Amended 29 June 2005)***.

All children attending as outpatients within the age range of the trial will be asked if the child is in this study and to provide the child’s vaccine study identity card and CISM demographic surveillance system ID card (see Section 5.1.10.3 for details of identity cards). If the identity cards are not available, the identity of the child will be confirmed against details held at the health facilities or on the CISM demographic database.

The child will then be referred for evaluation by the medical assistant or medical doctor, who will follow CISM SOPs for the examination, investigation and documentation of each presentation. This information will be recorded on a CISM clinic morbidity surveillance questionnaire. This questionnaire will provide a record of the child’s name and study number, key symptoms and signs, axillary temperature, results of the laboratory tests and imaging examinations available at the time of form completion, the diagnosis, the treatment prescribed and will state whether hospital admission was required.

These forms will be retained by the study personnel in the Health Center until they are sent to CISM at the end of each working day. At CISM, the questionnaires will be reviewed by the Principal Investigator or delegate. If it is necessary to notify any of these consultations as a serious adverse event, the Principal Investigator or delegate will review the child as necessary and complete an SAE form to notify the event (as specified in Section 8.5).

Children requiring inpatient care will be admitted to Manhiça District Hospital, their clinic course, treatment and results of further investigations will be documented in their hospital records.

5.1.14.2. In the community

Up until Clinic Visit 11 (approximately 3 months post Dose 3 of RTS,S/AS02D or Engerix-B), contacts with the study team will be frequent and SAEs will be documented through these visits. After Clinic Visit 11 (i.e. during the single-blind phase of the study) safety follow up will be by means of monthly visits by field workers and a final Clinic Visit at Month 14 (Clinic Visit 12). Field workers will visit each child once every month from 2 months after Dose 3 until study conclusion to maintain contact with the subject and his/her family and to ensure the complete detection of SAEs. The information will also be used to determine the period of residence within the study region and hence the period at risk for subjects. Absences from the study area of 2 weeks or more in duration will be recorded in multiples of 1 week.

During the field worker visits, the children's parent(s)/guardian(s) will be asked retrospectively if any SAEs occurred since the last visit and this information will be recorded. Unreported SAEs detected in this way will be investigated and reported by the Principal Investigator or delegate on the corresponding SAE forms (see Section 8.7). In the case of a death which has occurred at home, supplementary information will be gained using the verbal autopsy technique. The verbal autopsy will be conducted according to previously published methods and detailed in the SOPs on file with the investigators [Smith 1991].

If any child is reported to be unwell at the time of a visit, the field worker will advise the parent(s)/guardian(s) to seek care at either Ilha Josina Health Center***, Taninga Health Center*** or Manhiça District Hospital.. In the event that a child is seriously ill the field worker will inform the Principal Investigator or designate and transport arranged to Ilha Josina Health Center***, Taninga Health Center*** or Manhiça District Hospital, if judged appropriate by the responsible clinician ***(Amended 29 June 2005)***.

5.1.15. Provision of health care

Ilha Josina Health Center ***and Taninga Health Center*** provide~~s~~ primary health care between 8 am to 4 pm Monday to Friday. Medical attention is available on a 24 hour basis at Manhiça District Hospital. Subjects presenting 'out of hours' to Ilha Josina Health Center ***or Taninga Health Center*** will be provided with transport to Manhiça District Hospital. A detailed description of the facilities available at Manhiça District Hospital are provided in Section 4.1.5.1. A detailed description of the facilities available at Ilha Josina Health Center is provided in Section 4.1.5.2. ***A detailed description of the facilities available at Taninga Health Center is provided in Section 4.1.5.3 (Amended 29 June 2005)***.

Children who present unwell will be fully evaluated by medical personnel. Laboratory and radiological investigation will be carried out when appropriate. Treatment for medical conditions will be given according to the standard treatment regimes of Mozambique. Outpatient care will be delivered at the Manhiça District Hospital. Children requiring inpatient care will be admitted to the Manhiça District Hospital.

The Mozambican health services ask that the parent(s)/guardian(s) contribute to the costs of the child's care, this is a standard fee of 500 Mozambican Meticals (about 0.02 US Dollars) for outpatient medication. However, those families who cannot afford to pay will receive treatment free of charge. Care and medication given during hospitalization are free.

Manhiça District Hospital is able to provide a wide range of services and thus the need for referral to the specialist pediatric hospital in Maputo is rare. If this is considered necessary by the Principal Investigator or designate, CISM will facilitate the transfer of study subjects to Maputo Central Hospital.

5.1.15.1. Management and treatment of malaria in all subjects

Children with malaria will receive a regimen of sulfadoxine-pyrimethamine and amodiaquine. This regimen was recently introduced by the Mozambican Ministry of Health and has a high level of efficacy (refer to Section 4.1.5.3).

Children who require inpatient care or systemic treatment will receive treatment with intravenous quinine, which is standard practice and effective therapy in Mozambique.

5.1.16. Passive case detection of clinical cases of malaria

If a child presenting to Ilha Josina Health Center***, Taninga Health Center*** or Manhiça District Hospital is reported to have had fever within the preceding 24 hours or has a documented fever (defined as axillary temperature ≥ 37.5ºC) then blood will be collected for determination of malaria parasites and hemoglobin by the CISM laboratory staff. Duplicate blood slides will be taken and labeled with the same unique laboratory ID number, which will also be recorded on the CISM clinic morbidity surveillance questionnaire. Both slides will be taken to the CISM Laboratory and stained with Giemsa Stain. One will be read immediately (within 2 hours) and the result reported to guide diagnosis and management. The second slide will be kept for a later reading, according to CISM SOPs, and will determine parasite density for data analysis. Both slides will be stored at CISM. In addition, blood samples will be taken and stored for parasite genotyping ***(Amended 29 June 2005)***.

The clinical case definition for malaria currently used at Ilha Josina Health Center, ***Taninga Health Center*** and Manhiça District Hospital is a history of fever within the previous 24 hours or documented fever (temperature ≥ 37.5°C) at the time of presentation plus any level of asexual *P. falciparum* parasitemia. This will not be changed for the trial and all cases meeting this case definition will receive treatment for malaria ***(Amended 29 June 2005)***.

Axillary temperature measurements will be taken throughout this study. It is widely used in the health care provision system because it does not require sterilization of equipment between use. In addition, rectal temperature measurement is not acceptable in many African cultures. Axillary temperature measurement has long been used in malaria research in Africa: consequently, the disease rates and case definitions on which this trial

has been designed use axillary temperature measurement. It is therefore appropriate that this method continues to be used in this trial.

5.1.17. Active detection of infection

Four weeks prior to the start of surveillance for malaria infection (i.e. 2 weeks prior to Dose 3 of RTS,S/AS02A or Engerix-B), asymptomatic parasitemia will be cleared in children with sulfadoxine-pyrimethamine (SP) and Amodiaquine (each SP tablet contains 500 mg sulfadoxine and 25 mg pyrimethamine; ½ a tablet will be administered to each child to give a dose of 250 mg sulfadoxine and 12.5 mg pyrimethamine to each child. Amodiaquine will be administered to children as 10mg/kg body weight p.o. once daily for three days). The prevalence of asymptomatic parasitemia in the study area is high, therefore all children will receive a drug for clearance of malaria parasites without prior blood sampling to determine parasitemia.

One half of a tablet of SP will be administered to each child at Clinic Visit 7, two weeks before Dose 3 of RTS,S/AS02A or Engerix-B (Day 60). One dose of Amodiaquine will also be administered at Clinic Visit 7. Parents will be given two further doses of Amodiaquine to take with them and give to their child on the two subsequent days. The absence of parasitemia will be checked by taking blood for a blood slide two weeks later at Clinic Visit 8 (Day 74). Any child with any level of parasitemia at this point will be treated with a second-line treatment for malaria and will not undergo the surveillance period for active detection of infection. All such children will not contribute to the secondary endpoint for proof-of-concept.

The surveillance period for malaria infection will begin at Day 90 (Month 3, Clinic Visit 9), 14 days after the third dose of vaccine. Surveillance will continue with Clinic Visit 10 on Day 104 (Month 3½) and then field worker visits every two weeks (one visit every 14 days) for the following 2~~½~~ months (~~six~~ ***four*** visits) and ending with Clinic Visit 11 at Month 6. Blood samples will be taken for examination of malaria parasitemia and parasite genotyping at each ADI visit. The first blood slide examination will be made on the first day of surveillance, i.e. 14 days post Dose 3 of RTS,S/AS02A or Engerix-B (Day 90, Clinic Visit 9). At first detection of malaria infection with asexual forms of *P. falciparum* in a child, either by field worker surveillance or on clinical presentation with malaria disease at a health facility, the child will be considered to be infected and no further visits for surveillance of infection will be undertaken. The child will still be under morbidity surveillance and will receive monthly visits by field workers for SAE surveillance ***(Amended 12 April 2005)***.

For the surveillance period for malaria infection, a field worker visit will consist of visiting the child at home and completing a brief surveillance for infection morbidity questionnaire, which will include the reporting of malaria symptoms and a record of axillary temperature. If the child is well and afebrile, a ~~capillary~~ ***(Amended 12 April 2005)*** blood-sample will be taken and examined for malaria parasitemia and parasite genotyping. These blood slides will be Giemsa-stained and read to determine the presence of parasites at Ilha Josina Health Facility ***or Taninga Health Center***. Treatment for children not considered to be clinically ill will be sent by a field worker to all blood-

slide positive children within 36 hours of the original blood slide being taken ***(Amended 29 June 2005)***.

In the event that a child is reported as having a history of fever within the preceding 24 hours or has a documented axillary temperature of ≥37.5°C, no blood slides will be taken by the field worker but transport will be arranged by a project vehicle to Ilha Josina Health Facility ***or Taninga Health Center***. The surveillance for infection morbidity questionnaire will record that the child was transferred to Ilha Josina Health Facility ***or Taninga Health Center, as appropriate (Amended 29 June 2005)***. There the child will be reviewed by a clinician or medical agent or assistant, who will complete the CISM clinic morbidity surveillance questionnaire and the child will be managed according to the procedures detailed above (see Section 5.1.14) and a ~~capillary~~ ***(Amended 12 April 2005)*** blood sample will be taken for determination of parasitemia and parasite genotyping. If parasitemia is detected in the blood slides taken on arrival at Ilha Josina Health Facility ***or Taninga Health Center***, the child will be treated as appropriate ***(Amended 29 June 2005)***.

For data analysis purposes related to the study, all slides will be transferred to CISM for parasite density reading and storage.

5.2. Subject identification

Subject numbers will be issued sequentially to subjects at screening. Treatment numbers are assigned at the time of the first dose of TETRActHib in order of administration of dose.

Subject numbers will be issued consecutively to all children who are consented for screening. To identify the infant at subsequent contacts, each child’s parent(s)/guardian(s) will be issued with an identification card with the picture of the parent(s)/guardian(s) with the child attached to it. This card will bear the child’s subject number and an identifier used by CISM in their demographic database, the “permanent study area ID”.

The vaccines RTS,S/AS02D and Engerix-B will be identified by a treatment number. The unique treatment number will identify all doses of the vaccine administered to each subject. The treatment number will be issued sequentially at Clinic Visit 2. The treatment number will be recorded on the clinic form and subsequently the case report form documenting the linkage between the subject number and the treatment number.

5.3. Outline of study procedures

Table 20 List of study procedures

	**SCREEN**	**DOUBLE-BLIND PHASE**																	**SINGLE-BLIND PHASE**	
		**VACCINATION**													**ADI**					
**Study Months**		**0**					**1**				**2**				**3**		**4-5**	**6**	**7-13**	**14**
**Study days**	-14 to 0	0		14		21	30		44		60		74		90	104		180		
**Age (approx. weeks)**	6 to 8	8		10		11	12		14		16		18		20	22		~~30~~ ***32***		70
**Clinic Visit**	1	2		3		4	5		6		7		8		9	10		11		12
**Field Worker Visit Code #**			*21-26*		*27-31*			*32-37*		*38-43*		*44-49*		*50-55*			*56-59*		*60-66*	
**STUDY PROCEDURES**																				
Informed consent	●																			
Check of inclusion/ exclusion criteria	*	●																		
Medical history	*	●																		
Physical examination	*	●		*		*	*		*		*		*			*		*		*
Measure body weight		●					*				*					●		*		●
Check contraindications to vaccination		●		●			●		●		●		●							
Temperature		●	●	●	●	●	●	●	●	●	●	●	●	●						
Randomization		●																		
Vaccination—RTS,S/AS02D or Engerix-B				●					●				●							
Vaccination—TETRActHib		●					●				●									
Check of elimination criteria				●		●	●		●		●		●			●		●		●
Recording of medication		●	●	●	●	●	●	●	●	●	●	●	●	●		●		●		●
**SAFETY DATA COLLECTION**																				
Post-vaccination recording of solicited symptoms by investigator (Day 0)		●		●			●		●		●		●							
Solicited symptoms by investigator (Day 6)						●														
Solicited symptoms by field workers			●		●			●		●		●		●						
Recording of unsolicited AEs up to 1 month (min. 14 days) post-vaccination by investigator				●		●	●		●		●		●			●				
Morbidity surveillance/ recording of SAEs	● [b]	●	●	●	●	●	●	●	●	●	●	●	●	●	●	●	●	●	●	●
**ACTIVE DETECTION OF INFECTION**																				
Administer drug for clearance											●									
History of fever (for ADI)															●	●	●	●		
Temperature (for ADI)															●	●	●	●		
**LABORATORY ANALYSES**																				
Blood sampling	●					●							●		●	●	●	●		●
Complete blood count [a]	●					●										●		●		●
Creatinine, ALT, bilirubin	●					●										●		●		●
Antibodies to HBs	●															●				●
Antibodies to CS	●															●		●		●
Antibodies to Pertussis	●														●					
Antibodies to Diphtheria															●					
Antibodies to PRP															●					
Antibodies to Tetanus															●					
Smear for parasite density [c]													●		●	●	●	●		
Parasite genotyping													●		●	●	●	●		
Cell-Mediated Immunity	●															●		●		
~~Determinants of vaccine response~~						~~●~~														
**INTERIM ANALYSIS SAFETY & immunogenicity**																●				
**FINAL ANALYSIS**																		●		
**STUDY CONCLUSION**																				●

for notes, please see overleaf
(Amended 12 April 2005)

ADI; Active Detection of Infection
[a] Includes analysis of hemoglobin, total white cell count and platelets
[b] SAEs related to study procedures will be collected
[c] If a child presenting to a health center reports fever within the preceding 24 hours or has a documented fever (axillary temperature ≥ 37.5°C) then blood will be taken for parasitemia determination and hemoglobin
[d] To be carried out on first 100 children vaccinated only
● is used to indicate a study procedure that requires documentation in the individual CRF and ○ is used to indicate a study procedure that does not require documentation in the individual CRF. *SAEs that are related to study participation or are related to a concurrent medication will be collected and recorded from the time the child's' parent / guardian consents for them to participate in the study until they are discharged

It is the investigator's responsibility to ensure that the intervals between visits/contacts are strictly followed. These intervals determine each subject's evaluability in the according to protocol analyses (see Sections 4.3 to 4.6 and Section 10.4 for details of criteria for evaluability and cohorts to be analyzed). The intervals are tabulated in Table 21.

Table 21 Intervals between study visits

Interval	Length of interval
Clinic Visit 1 → Clinic Visit 2	0 to 14 days
Clinic Visit 2 → Clinic Visit 3	12 to 21 days
Clinic Visit 3 → Clinic Visit 4	5 to 9 days
Clinic Visit 3 → Clinic Visit 5	12 to 21 days
Clinic Visit 5 → Clinic Visit 6	12 to 21 days
Clinic Visit 6 → Clinic Visit 7	12 to 21 days
Clinic Visit 7 → Clinic Visit 8	12 to 21 days
Clinic Visit 7 → Clinic Visit 9	28 to 35 days
Clinic Visit 8 → Clinic Visit 10	28 to 35 days
Clinic Visit 8 → Clinic Visit 11	3.5 months ± 1 month
Clinic Visit 11 → Clinic Visit 12	8 months ± 2 months

5.4. Detailed description of study stages/visits

When materials are provided by GSK Biologicals, it is **MANDATORY** that all clinical samples (including serum samples) will be collected and stored using exclusively those materials in the appropriate manner. The use of other materials could result in the exclusion of the subject from the ATP analysis (see Section 10.6 for definition of study cohorts to be evaluated). The investigator must ensure that his/her personnel and the laboratory(ies) under his/her supervision comply with this requirement. However, when GSK Biologicals does not provide material for collecting and storing clinical samples, then appropriate materials from the investigator's site are to be used. Refer to Appendix D and Appendix E.

The subjects' parents/guardians will be instructed to contact the investigator immediately should the subject manifest any signs or symptoms they perceive as serious.

Clinic Visit 1: Screening
Day -14 to Day 0

- Obtain signed/thumbprinted informed consent from the parent(s)/guardians
- Check inclusion/exclusion criteria
- Take medical history and carry out physical examination

- Record any SAEs that may have occurred as a result of study procedures
- ~~Capillary~~ Blood sample to collect a minimum ~~1~~ ***2*** mL blood for analysis of:
  - hematology (complete blood count)
  - biochemistry (creatinine, ALT and bilirubin)
  - serology (antibodies to CS**,** ~~and~~ antibodies to HBs ***and pertussis***)
  - cell-mediated immunity

(Amended 12 April 2005)

Clinic Visit 2: Dose 1 of TETRActHib
Day 0

- Check study identification card of vaccinee
- Check inclusion/exclusion criteria
- Take medical history and carry out physical examination
- Measure body weight
- Check contraindications to vaccination
- Record pre-vaccination body temperature
- Randomize subjects
- Administer the first dose of TETRActHib intramuscularly in the right thigh

Each infant will be assessed for at least 60 minutes after vaccination to evaluate and treat any acute adverse events.

- Record any post-vaccination solicited symptoms
- Record any post-vaccination unsolicited adverse events
- Record any post-vaccination SAEs
- Record concomitant medication

Field worker post vaccination follow up visits 21 to 26
Days 1, 2, 3, 4, 5 and 6

- Record axillary temperature of subject
- Record local (pain and swelling at the injection site) and general (fever, irritability / fussiness, drowsiness, loss of appetite) solicited adverse events
- Record SAEs experienced by the vaccinee since the last visit
- Record unsolicited adverse events experienced by the vaccinee since the last visit
- Record concomitant medication

Clinic Visit 3: Dose 1 of RTS,S/AS02D or Engerix-B
Day 14

- Check study identification card of vaccinee
- Carry out physical examination
- Record SAEs experienced by the vaccinee since the last visit
- Record any unsolicited adverse events since the previous vaccination
- Check contraindications to vaccination
- Check elimination criteria
- Record pre-vaccination body temperature
- Administer the first dose of RTS,S/AS02D or Engerix-B intramuscularly in the left thigh

Each infant will be assessed for at least 60 minutes after vaccination to evaluate and treat any acute adverse events.

- Record any post-vaccination solicited symptoms
- Record any post-vaccination unsolicited adverse events
- Record any post-vaccination SAEs
- Record concomitant medication

Field worker post vaccination follow up visits 27 to 31
Days 15, 16, 17, 18 and 19

- Record axillary temperature of subject
- Record local (pain and swelling at the injection site) and general (fever, irritability / fussiness, drowsiness, loss of appetite) solicited adverse events
- Record SAEs experienced by the vaccinee since the last visit
- Record unsolicited adverse events experienced by the vaccinee since the last visit
- Record concomitant medication

Clinic Visit 4: Collection of blood sample
Day 20

- Check study identification card of vaccinee
- Carry out physical examination
- Record axillary temperature of subject
- Record local (pain and swelling at the injection site) and general (fever, irritability / fussiness, drowsiness, loss of appetite) solicited adverse events
- Record SAEs experienced by the vaccinee since the last visit
- Record unsolicited adverse events experienced by the vaccinee since the last visit

- Check elimination criteria
- Record concomitant medication
- ~~Capillary~~ Blood sample to collect a minimum 1 mL blood for analysis of
  - hematology (complete blood count)
  - biochemistry (creatinine, ALT and bilirubin)
  - ~~determinants of vaccine response~~

(Amended 12 April 2005).

Clinic Visit 5: Dose 2 of TETRActHib
Day 30

- Check study identification card of vaccinee
- Carry out physical examination
- Measure body weight
- Record SAEs experienced by the vaccinee since the last visit
- Record any unsolicited adverse events since the previous vaccination
- Check contraindications to vaccination
- Check elimination criteria
- Record pre-vaccination body temperature
- Administer the second dose of TETRActHib intramuscularly in the right thigh

Each infant will be assessed for at least 60 minutes after vaccination to evaluate and treat any acute adverse events.

- Record any post-vaccination solicited symptoms
- Record any post-vaccination unsolicited adverse events
- Record any post-vaccination SAEs
- Record concomitant medication

Field worker post vaccination follow up visits 32 to 37
Days 31, 32, 33, 34, 35 and 36

- Record axillary temperature of subject
- Record local (pain and swelling at the injection site) and general (fever, irritability / fussiness, drowsiness, loss of appetite) solicited adverse events
- Record SAEs experienced by the vaccinee since the last visit
- Record unsolicited adverse events experienced by the vaccinee since the last visit
- Record concomitant medication

Clinic Visit 6: Dose 2 of RTS,S/AS02D or Engerix-B
Day 44

- Check study identification card of vaccinee
- Carry out physical examination
- Record SAEs experienced by the vaccinee since the last visit
- Record any unsolicited adverse events since the previous vaccination
- Check contraindications to vaccination
- Check elimination criteria
- Record pre-vaccination body temperature
- Administer the second dose of RTS,S/AS02D or Engerix-B intramuscularly in the left thigh

Each infant will be assessed for at least 60 minutes after vaccination to evaluate and treat any acute adverse events.

- Record any post-vaccination solicited symptoms
- Record any post-vaccination unsolicited adverse events
- Record any post-vaccination SAEs
- Record concomitant medication

Field worker post vaccination follow up visits 38 to 43
Days 45, 46, 47, 48, 49 and 50

- Record axillary temperature of subject
- Record local (pain and swelling at the injection site) and general (fever, irritability / fussiness, drowsiness, loss of appetite) solicited adverse events
- Record SAEs experienced by the vaccinee since the last visit
- Record unsolicited adverse events experienced by the vaccinee since the last visit
- Record concomitant medication

Clinic Visit 7: Dose 3 of TETRActHib with the administration of sulfadoxine-pyrimethamine for presumptive clearance of malaria parasitemia
Day 60

- Check study identification card of vaccinee
- Carry out physical examination
- Measure body weight
- Record SAEs experienced by the vaccinee since the last visit
- Record any unsolicited adverse events since the previous vaccination
- Check contraindications to vaccination

- Check elimination criteria
- Record pre-vaccination body temperature
- Treat with antimalarial without checking for parasitemia (administer dose of sulfadoxine-pyrimethamine and first dose of Amodiaquine; provide subject's parent(s)/guardian(s) with doses of Amodiaquine for administration to their child on the two subsequent days)
- Administer the third dose of TETRActHib intramuscularly in the right thigh

Each infant will be assessed for at least 60 minutes after vaccination to evaluate and treat any acute adverse events.

- Record any post-vaccination solicited symptoms
- Record any post-vaccination unsolicited adverse events
- Record any post-vaccination SAEs
- Record concomitant medication

Field worker post vaccination follow up visits 44 to 49
Days 61, 62, 63, 64, 65 and 66

- Record axillary temperature of subject
- Record local (pain and swelling at the injection site) and general (fever, irritability / fussiness, drowsiness, loss of appetite) solicited adverse events
- Record SAEs experienced by the vaccinee since the last visit
- Record unsolicited adverse events experienced by the vaccinee since the last visit
- Record concomitant medication

Clinic Visit 8: Dose 3 of RTS,S/AS02D or Engerix-B
Check for clearance of malaria parasitemia
Day 74

- Check study identification card of vaccinee
- Carry out physical examination
- Record SAEs experienced by the vaccinee since the last visit
- Record any unsolicited adverse events since the previous vaccination
- Check contraindications to vaccination
- Check elimination criteria
- Record pre-vaccination body temperature
- Administer the third dose of RTS,S/AS02D or Engerix-B intramuscularly in the left thigh

Each infant will be assessed for at least 60 minutes after vaccination to evaluate and treat any acute adverse events.

- Record any post-vaccination solicited symptoms
- Record any post-vaccination unsolicited adverse events
- Record any post-vaccination SAEs
- Record concomitant medication
- ~~Capillary~~ Blood sample to collect a minimum 1 mL blood for ***(Amended 12 April 2005)***:
  - blood film for parasitemia determination
  - parasite genotyping

**Any subjects who are determined to be positive for asexual *P. falciparum* parasitemia (parasitemia > 0) are NOT to be included in ADI**

Field worker post vaccination follow up visits 50 to 55
Days 75, 76, 77, 78, 79 and 80

- Record axillary temperature of subject
- Record local (pain and swelling at the injection site) and general (fever, irritability / fussiness, drowsiness, loss of appetite) solicited adverse events
- Record SAEs experienced by the vaccinee since the last visit
- Record unsolicited adverse events experienced by the vaccinee since the last visit
- Record concomitant medication

Clinic Visit 9: First Clinic Visit for ADI
Day 90

- Record history of fever for subject
- Record axillary temperature of subject for ADI
- Record SAEs experienced by the vaccinee since the last visit
- ~~Capillary~~ Blood sample to collect a minimum 1 mL blood for ***(Amended 12 April 2005)***:
  - blood film for parasitemia determination
  - parasite genotyping
  - serology (antibodies to Pertussis, Diphtheria, PRP, and Tetanus)

Clinic Visit 10: Second Clinic Visit for ADI
Day 104, Month 3½

- Check study identification card of subject
- Carry out physical examination
- Check elimination criteria
- Record concomitant medication

- Measure body weight
- Record SAEs experienced by the vaccinee since the last visit
- Record any unsolicited adverse events since the previous vaccination
- Record history of fever for subject
- Record axillary temperature of subject for ADI
- ~~Capillary~~ Blood sample to collect a minimum ~~1~~ ***2*** mL whole blood for analysis of ***(Amended 12 April 2005)***:
  - hematology (complete blood count)
  - biochemistry (creatinine, ALT and bilirubin)
  - blood film for parasitemia determination
  - serology (antibodies to CS and HBs)
  - parasite genotyping
  - cell-mediated immunity

Monthly field-worker home visits (for ADI) 56 to 59
Month 4, 4½, 5, 5½

- Record SAEs experienced by the vaccinee since the last visit
- Record history of fever for subject
- Record axillary temperature of subject for ADI
- ~~Capillary~~ Blood sample to collect a minimum 1 mL blood for ***(Amended 12 April 2005)***:
  - blood film for parasitemia determination
  - parasite genotyping

Clinic Visit 11: Final Visit for ADI
Day 180, Month 6

- Check study identification card of subject
- Carry out physical examination
- Check elimination criteria
- Record concomitant medication
- Measure body weight
- Record SAEs experienced by the vaccinee since the last visit
- Record history of fever for subject
- Record axillary temperature of subject for ADI
- ~~Capillary~~ Blood sample to collect a minimum ~~1~~ ***2*** mL whole blood for analysis of ***(Amended 12 April 2005)***:

- hematology (complete blood count)
- biochemistry (creatinine, ALT and bilirubin)
- serology (antibodies to CS)
- blood film for parasitemia determination
- parasite genotyping
- cell-mediated immunity

Monthly field-worker home visits 60 to 66
Months 7 to 13

- Record SAEs experienced by the vaccinee since the last visit

Clinic Visit 12: Final Study Visit
Month 14

- Carry out physical examination
- Check elimination criteria
- Record concomitant medication
- Measure body weight
- Record SAEs experienced by the vaccinee since the last visit
- ~~Capillary~~ Blood sample to collect a minimum 1 mL whole blood for analysis of ***(Amended 12 April 2005)***:
  - hematology (complete blood count)
  - biochemistry (creatinine, ALT and bilirubin)
  - serology (antibodies to CS and antibodies to HBs)

5.5. Sample handling and analysis

5.5.1. Treatment and storage of biological samples

See Appendix D of the protocol for details of treatment and storage of biological samples

See Appendix E for instructions for shipment of biological samples

5.5.2. Laboratory assays

Laboratory tests at screening: Clinic Visit 1
Day -14 to Day 0

Collection of a minimum ~~1~~ ***2*** mL whole blood for baseline analysis of:

- hematology (complete blood count)

- biochemistry (creatinine, ALT and bilirubin)
- serology (antibodies to CS**,** ~~and~~ antibodies to HBs ***and pertussis***)
- cell-mediated immunity

(Amended 12 April 2005)

Laboratory tests during the study: Clinic Visit 4
Day 20

~~Capillary~~ Blood sample to collect a minimum 1 mL blood for analysis of:

- hematology (complete blood count)
- biochemistry (creatinine, ALT and bilirubin)
- ~~determinants of vaccine response~~

(Amended 12 April 2005)

Clinic Visit 8: Dose 3 of RTS,S/AS02D or Engerix-B
Check for clearance of malaria parasitemia
Day 74

~~Capillary~~ Blood sample to collect a minimum 1 mL blood for analysis of ***(Amended 12 April 2005)***:

- blood film for parasitemia determination
- parasite genotyping

Clinic Visit 9: First Clinic Visit for ADI
Day 90

~~Capillary~~ Blood sample to collect a minimum 1 mL blood for analysis of ***(Amended 12 April 2005)***:

- blood film for parasitemia determination
- parasite genotyping
- serology (antibodies to Pertussis, Diphtheria, PRP and Tetanus)

Clinic Visit 10
Month 3½

~~Capillary~~ Blood sample to collect a minimum ~~1~~ ***2*** mL blood for analysis of:

- hematology (complete blood count)
- biochemistry (creatinine, ALT and bilirubin)
- blood film for parasitemia determination
- serology (antibodies to CS and HBs)

- parasite genotyping
- cell-mediated immunity

Monthly field-worker home visits (for ADI) 56 to 59
Month 4, 4½, 5, 5½

~~Capillary~~ Blood sample to collect a minimum 1 mL blood for analysis of ***(Amended 12 April 2005)***:

- blood film for parasitemia determination
- parasite genotyping

Clinic Visit 11: Final Visit for ADI
Month 6

~~Capillary~~ Blood sample to collect a minimum ~~1~~ ***2*** mL blood for analysis of ***(Amended 12 April 2005)***:

- hematology (complete blood count)
- biochemistry (creatinine, ALT and bilirubin)
- serology (antibodies to CS)
- blood film for parasitemia determination
- parasite genotyping
- cell-mediated immunity

Clinic Visit 12: Final Study Visit
Month 14

~~Capillary~~ Blood sample to collect a minimum 1 mL blood for analysis of ***(Amended 12 April 2005)***:

- hematology (complete blood count)
- biochemistry (creatinine, ALT and bilirubin)
- serology (antibodies to CS and antibodies to HBs)

Table 22 Summary of laboratory immunology tests to be performed

Assay	Marker	Assay method	Test Kit/ Manufacturer	Assay unit	Assay cut-off	Laboratory
Anti-CS antibodies	R32LR	ELISA	In-house ELISA	EU/mL	0.5	Leroux-Roels Laboratory, Ghent, Belgium
Anti-HBs antibodies		EIA	AUSAB EIA ABBOTT†	mIU/mL	10**	GSK Bio*, Rixensart
Anti-Pertussis antibodies		ELISA	IgG EIA (ICN-FLOW)	EL.U/mL	15	GSK Bio*, Rixensart
Anti-Diphtheria antibodies		ELISA	In-house ELISA	IU/mL	0.1	GSK Bio*, Rixensart
Anti-PRP antibodies		ELISA	In-house ELISA	µg/mL	0.15	GSK Bio*, Rixensart
Anti-Tetanus antibodies		ELISA	In-house ELISA	IU/mL	0.1	GSK Bio*, Rixensart
CS-specific CMI		ICS	In-house ICS	% cytokine positive cells		CISM or GSK Bio*
~~Determinants of vaccine response~~		~~high throughput genotyping~~	~~TBD~~	~~TBD~~		~~CISM*~~

* or designated validated laboratory
** seroprotective level
† or equivalent
CMI: cell-mediated immunity
ELISA: Enzyme-linked Immunoabsorbent Assay
EIA: Enzyme Immunoassay
ICS: intra-cellular cytokine staining
(Amended 12 April 2005)

Separation of serum from the blood samples will be performed at the investigator's center.

Hematology and biochemistry determination will be performed at the CISM laboratories. The acceptable reference ranges will be specified in an SOP at the investigator site and in the GSK central study file.

Total antibodies against the Hib polysaccharide PRP will be measured by ELISA using an in-house assay of GSK Biologicals. The cut-off is 0.15 µg/mL.

Specific antibodies against diphtheria and tetanus toxoid will be measured by ELISA techniques by GSK Biologicals. The cut-off of the tests is 0.1 IU/mL (Camargo 1984, Melville-Smith 1983).

Anti-BPT antibody titers will be determined by ELISA using the IgG EIA test kit Lab Systems by GSK Biologicals, and expressed in EL.U/mL with an assay cut-off of 15 EL.U/mL.

CS-specific CMI will be determined at CISM by intra-cellular cytokine staining (CD4, CD8, CD3 and IFN-gamma) and measured as frequency (percent) of cytokine positive cells.

~~The determinants of vaccine response will be analyzed by high throughput genotyping including Sequenom multiplexing, denaturing high performance liquid chromatography,~~

~~Taqman®, restriction enzyme digestions, and sequencing. This will be done at the CISM laboratory, the Center for International Health Laboratory, University of Barcelona, or a designated validated laboratory.~~

(Amended 12 April 2005)

5.5.3. Serology plan

Serological responses will be primarily measured by evaluating antibody responses to CS (anti-CS also referred to as anti-R32LR antibodies). Antibody levels will be measured by standard Enzyme Linked Immunosorbent Assay (ELISA) methodologies using plate adsorbed R32LR antigen with a standard reference antibody as a control. Results will be reported in EU/mL. Anti-HBsAg antibody levels will be measured by ELISA with a commercial AUSAB EIA kit from Abbott or equivalent.

Serum for antibody determination will be collected by ~~capillary~~ blood sample (as specified at each visit in Sections 5.4 and 5.5). Samples for safety will be analyzed at the time they are collected ***(Amended 12 April 2005)***.

Serum samples from each child will be shipped to GSK Biologicals, Rixensart, Belgium.

Any serum not immediately used in antibody assays will be stored at -20°C or less and would only be used to assess the immune response to vaccination or to assess any potential toxicity of the vaccine (Assay details are provided in Appendix F).

Table 23 Summary of blood sampling timepoints/*immunological assays (Amended 12 April 2005)*

Blood sampling timepoint			Test	No. subjects	Laboratory	Priority Rank
**Timing**	**Timepoint**	**Clinic Visit No.**				
Pre Dose 1	Screening	1	Anti-HBs antibodies	~~200~~ ***220***	GSK Bio*	***3*** ~~2~~
			Anti-CS antibodies	~~200~~ ***220***	GSK Bio*	***2*** ~~1~~
			Anti-Pertussis Antibodies	***200***	***GSK Bio****	***1***
			CMI	~~200~~ ***220***	CISM	***4*** ~~3~~
~~Post Dose 1~~	~~Month 0~~	4	~~Determinants of Vaccine Response~~	~~200~~	~~CISM*~~	~~1~~
Post Dose 3	Month 3	9	Anti-PRP antibodies	~~200~~ ***220***	GSK Bio*	1
			Anti-Pertussis antibodies	~~200~~ ***220***	GSK Bio*	2
			Anti-Diphtheria antibodies	~~200~~ ***220***	GSK Bio*	3
			Anti-Tetanus antibodies	~~200~~ ***220***	GSK Bio*	4
Post Dose 3	Month 3½	10	Anti-HBs antibodies	~~200~~ ***220***	GSK Bio*	2
			Anti-CS antibodies	~~200~~ ***220***	GSK Bio*	1
			CMI	~~200~~ ***220***	~~GSK Bio*~~ ***CISM***	3
Post Dose 3	Month 6	11	Anti-CS antibodies	~~200~~ ***220***	GSK Bio*	1
			CMI	~~200~~ ***220***	CISM	2
Post Dose 3	Month 14	12	Anti-HBs antibodies	~~200~~ ***220***	GSK Bio*	1
			Anti-CS antibodies	~~200~~ ***220***	GSK Bio*	2

*or designated laboratory
CMI: cell-mediated immunity
(Amended 12 April 2005)

6. INVESTIGATIONAL PRODUCTS AND ADMINISTRATION

6.1. Study vaccines

The candidate vaccine to be used has been developed and manufactured by GSK Biologicals.

The Quality Control Standards and Requirements for the candidate vaccine are described in separate release protocols and the required approvals have been obtained.

Commercial vaccines are assumed to comply with the specifications given in the manufacturer's Summary of Product Characteristics.

Refer to Appendix G for details of vaccine supplies.

6.1.1. RTS,S/AS02D candidate malaria and hepatitis B vaccine

RTS,S antigen presentation:

- The lyophilized antigen pellet contains 31.25 µg of RTS,S with 12.6 mg of sucrose as cryoprotectant per 3 mL monodose vial. The pellet is reconstituted with adjuvant in liquid form and 0.5 mL of reconstituted vaccine contains 25µg RTS,S.

AS02D adjuvant:

- AS02D contains 25 µg of MPL®, 25 µg QS21 (QS21 is a triterpene glycoside purified from the bark of Quillaja saponaria) and 125 µL of a proprietary oil-in-water emulsion in phosphate buffered saline per 0.5 mL, presented in prefilled syringes.

A dose of 0.5 mL will be delivered. The presentation of the reconstituted RTS,S/AS02D candidate malaria and hepatitis B vaccine is an opaque milky liquid.

6.1.2. TETRActHib

TETRActHib™ is licensed by Aventis Pasteur, Lyon. The vaccine is presented as one vial of Act-HIB *Hemophilus influenzae* type b polysaccharide conjugated to tetanus protein as a powder and one pre-filled syringe (PFS) of D.T.COQ/D.T.P. (adsorbed diphtheria, tetanus and pertussis vaccine, Aventis Pasteur, Lyon). Prior to vaccination the D.T.COQ/D.T.P. is mixed with the Act-HIB powder.

The vaccine composition is;

Act-HIB (Powder for one vaccinating dose)

- *Hemophilus influenzae* type b polysaccharide conjugated with tetanus protein 10 µg
- Sucrose and trometamol

D.T.COQ/D.T.P. (Suspension for injection for one vaccinating dose (0.5 mL)

- Purified diphtheria toxoid not less than 30 IU
- Purified tetanus toxoid not less than 60 IU
- *Bordetella pertussis* not less than 4 IU
- Aluminum hydroxide, thiomersal and buffer solution containing sodium chloride, dihydrate sodium hydrogen phosphate, potassium dihydrogen phosphate, acetic acid and/or sodium hydroxide and water for injections.

A dose of 0.5 mL will be delivered. The presentation of the reconstituted TETRActHib vaccine is a whitish cloudy liquid.

6.1.3. Engerix-B

The pediatric dosage vial contains 10 μg of purified major hepatitis B surface antigen. It is prepared as a suspension of 0.5 mL volume per vial. The content, upon storage may present a fine white deposit with a clear colorless supernatant. Once shaken, the vaccine is slightly opaque.

6.2. Dosage and administration

6.2.1. RTS,S/AS02D (0.5 mL dose)

RTS,S/AS02D will be supplied such that the reconstituted vaccine volume will provide a 0.5 mL pediatric dose. One 0.5 mL dose will be aspirated from each vial and used.

Disinfect top of vaccine vial (pellet) with alcohol swabs and let dry. Inject complete contents of one PFS of diluent into vial of lyophilized vaccine. Remove and discard the syringe and needle under appropriate safety precautions. The pellet is then dissolved by gently shaking the vial. Wait for 1 minute to ensure complete dissolution of vial contents before withdrawing a sufficient volume to provide a 0.5 mL dose (volume required for RTS,S/AS02D) of the reconstituted vaccine solution using a fresh needle and syringe for injection. The reconstituted vaccine should be administered by slow IM injection, using a 25G needle with length of 1 inch (25 mm), in the left antero-lateral thigh within 4 hours of reconstitution (storage at 2°C to 8°C).

6.2.2. TETRActHib (0.5 mL dose)

Disinfect top of *Hemophilus* type b conjugate vaccine vial with alcohol swabs and let dry. Inject complete contents of one pre-filled syringe (PFS) of D.T.P. suspension (D.T.COQ/D.T.P.) into vial of *Hemophilus* type b conjugate vaccine. Remove and discard the syringe and needle with appropriate safety precautions. Shake before use to obtain a homogenous suspension. The vaccine should be withdrawn using a fresh needle for injection. The reconstituted vaccine should be administered by slow IM injection, using a 25G needle with length of 1 inch (25 mm), in the right antero-lateral thigh (storage at 2°C to 8°C).

6.2.3. Engerix-B (0.5 mL dose)

Disinfect the top of the vaccine vial with alcohol swabs and shake gently while letting the alcohol dry. Then withdraw, using a fresh syringe and needle the contents of the vial into a syringe for injection. Remove and discard the needle with appropriate safety precautions. Use a fresh needle for injection. The vaccine should be administered soon afterwards intramuscularly ~~in the deltoid region~~, using a 25G needle with length of 1 inch (25 mm) in the left antero-lateral thigh (storage at 2°C to 8°C) ***(Amended 12 April 2005)***.

6.3. Storage

ALL VACCINE VIALS/PRE-FILLED SYRINGES (RTS,S, Engerix-B, D.T.P. suspension [D.T.COQ/D.T.P.] and *Hemophilus influenzae* type B), AND ADJUVANTS (AS02D) MUST BE STORED IN THE REFRIGERATOR (+2°C to +8°C) AND MUST NOT BE FROZEN.

All vaccines will be stored in a safe and locked place with no access for unauthorized personnel. Storage temperature will be monitored daily, according to SOPs at the investigator's site. An alarm system and a back-up refrigerator will be available in case of power failure/breakdown.

The study monitor must be contacted if the cold chain is broken (e.g. vaccines become frozen or refrigeration fails).

Storage conditions for transport of vaccines from country medical department or dispatch center to study sites or between sites are described in Appendix D.

6.4. Treatment allocation and randomization

6.4.1. Randomization of supplies

A randomization list will be generated at GSK Biologicals, Rixensart, using a standard SAS® (Statistical Analysis System) program and will be used to assign the vaccines to treatment numbers. A 1:1 randomization of RTS,S/AS02D to Engerix-B will be used throughout the study.

6.4.2. Randomization of subjects

Subjects will be allocated sequentially to treatment numbers in the order that they present for vaccination.

6.5. Method of blinding and breaking the study blind

Data pertaining to RTS,S/AS02D or Engerix-B will be collected in a double blinded (observer blinded) manner; data relating to TETRActHib will be collected in an open fashion. 'Double blinded (observer blinded)' means that the vaccine recipient and their parent(s)/guardian(s) as well as those responsible for the evaluation of safety,

immunogenicity and efficacy endpoints will all be unaware which treatment, RTS,S/AS02D or Engerix-B, was administered to a particular subject. The only study staff aware of the vaccine assignment for RTS,S/AS02D or Engerix-B will be those responsible for the preparation and administration of vaccines; these staff will play no other role in the study.

Code break envelopes, for each study enrolled subject and associating each treatment number with a specific vaccine, will be kept in a safe and locked place with no access for unauthorized personnel by the Local Safety Monitor in Mozambique as well as by Central Safety at GlaxoSmithKline Biologicals, Rixensart.

If deemed necessary for reasons such as safety, the Local Safety Monitor in Mozambique as well as GSK Biologicals Central Safety will unblind the specific enrolled subject without revealing the study blind to the investigators.

One interim analysis will be conducted during the study: an analysis of Safety and Immunogenicity post Clinic Visit 10. To maintain the blind the interim analysis will be performed by a statistician independent of the GSK Biologicals, MVI and CISM clinical teams.

A formal reporting and analysis plan (RAP) will be developed. Once the study is completed and the GSK Biologicals reference database locked, GlaxoSmithKline Biologicals will be responsible for initiating the execution of the statistical analysis plan in collaboration with CISM and MVI and breaking the blind.

GSK Biologicals’ policy (incorporating ICH E2A guidance, EU Clinical Trial Directive and Federal Regulations) is to unblind any serious adverse event (SAE) report associated with the use of the investigational product, which is unexpected and attributable/suspected, prior to regulatory reporting. The Clinical Safety physician is responsible for unblinding the treatment assignment in accordance with specified time frames for expedited reporting of SAEs (Refer to Section 8.8).

6.6. Replacement of unusable vaccine doses

Additional vaccine doses will be provided to replace those that are unusable (see Appendix G for details of supplies).

In addition to the vaccine doses provided for the planned number of enrolled subjects, 5% additional doses will be supplied. In case a vaccine dose is broken or unusable, the investigator should replace it with a replacement vaccine dose. If a vaccine dose needs replacement, the envelope with the corresponding treatment number will designate the replacement without unblinding the study using a coded letter system. Although the sponsor need not be notified immediately in these cases, documentation of the use of the replacement vaccine must be recorded by the investigator on the vaccine administration page of the CRF and on the vaccine accountability form.

6.7. Packaging

See Appendix G.

6.8. Vaccine accountability

See Appendix G.

6.9. Concomitant medication/treatment

At each study visit/contact, the investigator should question the enrolled subject's parent(s)/guardian(s) about any medication(s) taken.

All antipyretic, analgesic, antibiotic and antimalarial drugs, administered at ANY time during the period starting with administration of each dose and ending 30 days after each dose are to be recorded with generic name of the medication (trade names are allowed for combination drugs, i.e., multi-component drugs), medical indication, total daily dose, route of administration, start and end dates of treatment.

All antimalarial drugs administered during the period starting at 31 days post Dose 3 of RTS,S/AS02D and ending with the last visit for ADI are to be recorded with generic name of the medication (trade names are allowed for combination drugs, i.e., multi-component drugs), medical indication, total daily dose, route of administration, start and end dates of treatment (Amended 12 April 2005).

Any treatments and/or medications which are listed as elimination criteria in Section 4.5, e.g., any immunoglobulins, other blood products and any immune modifying drugs administered within three months preceding the first dose or at any time during the study period are to be recorded with generic name of the medication (trade names are allowed for combination drugs only), medical indication, total daily dose, route of administration, start and end dates of treatment. Refer to Sections 4.4 and 4.5. ***The time periods between which each type of concommitant medication/treatment should be recorded is summarized in Table 24.***

Table 24 Summary of time periods between which different classes of concommitant medication/treatment must be recorded

3 months prior to Dose 1 → Dose 1	*All treatments listed as elimination criteria in Section 4.5**
Dose 1 → 30 Days post Dose 3	*All antipyretic, analgesic, antibiotic, antimalarial and any treatments listed as elimination criteria in Section 4.5**
31 Days post Dose 3 → last visit for ADI	*All antimalarial drugs and treatments listed as elimination criteria in Section 4.5**
Last visit for ADI → Final Study Visit	*All treatments listed as elimination criteria in Section 4.5**

** e.g. any immunoglobulins, other blood products and any immune modifying drugs*

(Amended 12 April 2005).

Any vaccine not foreseen in the study protocol administered in the period beginning 30 days preceding each dose and ending 30 days after each dose is to be recorded with trade name, route of administration and date(s) of administration. Refer to Sections 4.4 and 4.5.

Any concomitant medication administered prophylactically in anticipation of reaction to the vaccination must be recorded in the CRF with generic name of the medication (trade names are allowed for combination drugs only), total daily dose, route of administration, start and end dates of treatment and coded as ‘Prophylactic’.

7. HEALTH ECONOMICS

Not applicable

8. ADVERSE EVENTS AND SERIOUS ADVERSE EVENTS

The investigator is responsible for the detection and documentation of events meeting the criteria and definition of an adverse event (AE) or serious adverse event (SAE) as provided in this protocol. During the study, when there is a safety evaluation, the investigator or site staff will be responsible for detecting AEs and SAEs, as detailed in this section of the protocol.

Each enrolled subject’s parent(s)/guardian(s) will be instructed to contact the investigative team at CISM immediately should the enrolled subject manifest any signs or symptoms they perceive as serious.

8.1. Definition of an adverse event

An AE is any untoward medical occurrence in a clinical investigation subject, temporally associated with the use of a medicinal product, whether or not considered related to the medicinal product.

An AE can therefore be any unfavorable and unintended sign (including an abnormal laboratory finding), symptom, or disease (new or exacerbated) temporally associated with the use of a medicinal product.

Examples of an AE include:

- Exacerbation of a chronic or intermittent pre-existing condition including either an increase in frequency and/or intensity of the condition.
- New conditions detected or diagnosed after investigational product administration even though it may have been present prior to the start of the study.
- Signs, symptoms, or the clinical sequelae of a suspected overdose of either investigational product or a concurrent medication (overdose per se should not be reported as an AE/SAE).
- Signs, symptoms temporally associated with vaccine administration.

AEs may include pre- or post-treatment events that occur as a result of protocol-mandated procedures (i.e., invasive procedures, modification of subject’s previous therapeutic regimen).

N.B. AEs to be recorded as endpoints (solicited events) are described in Section 8.4.1. All other AEs will be recorded as **UNSOLICITED AEs**.

Example of events to be recorded in the medical history section of the CRF:

Pre-existing conditions or signs and/or symptoms present in a subject prior to the start of the study (i.e. prior to the first study procedure) should be recorded in the medical history section of the subject's CRF.

8.2. Definition of a serious adverse event

A serious adverse event (SAE) is any untoward medical occurrence that:

a) results in death;

b) is life-threatening;

NOTE: The term 'life-threatening' in the definition of 'serious' refers to an event in which the subject was at risk of death at the time of the event. It does not refer to an event, which hypothetically might have caused death, if it were more severe.

c) requires hospitalization or prolongation of existing hospitalization;

NOTE: In general, hospitalization signifies that the subject has been detained (usually involving at least an overnight stay) at the hospital or emergency ward for observation and/or treatment that would not have been appropriate in the physician's office or out-patient setting. Complications that occur during hospitalization are AEs. If a complication prolongs hospitalization or fulfills any other serious criteria, the event is serious. When in doubt as to whether 'hospitalization' occurred or was necessary, the AE should be considered serious.

Hospitalization for elective treatment of a pre-existing condition that did not worsen from baseline is not considered an AE.

d) results in disability/incapacity;

NOTE: The term disability means a substantial disruption of a person's ability to conduct normal life functions. This definition is not intended to include experiences of relatively minor medical significance such as uncomplicated headache, nausea, vomiting, diarrhea, influenza, and accidental trauma (e.g. sprained ankle) which may interfere or prevent everyday life functions but do not constitute a substantial disruption.

e) is a congenital anomaly/birth defect in the offspring of a study subject;

f) medical or scientific judgement should be exercised in deciding whether reporting is appropriate in other situations, such as important medical events that may not be immediately life-threatening or result in death or hospitalization but may jeopardize the subject or may require medical or surgical intervention to prevent one of the other outcomes listed in the above definition. These should also be considered serious.

Examples of such events are invasive or malignant cancers, intensive treatment in an emergency room or at home for allergic bronchospasm, blood dyscrasias or convulsions that do not result in hospitalization.

In this study all seizures occurring within a 30-day period of vaccination will be notified as SAEs. Complete data collection of seizures is sought to pilot an analysis proposed by the Brighton Collaboration [Bonhoeffer, 2004]. Key information pertaining to seizures will be documented in the CRF.

8.3. Clinical laboratory parameters and other abnormal assessments qualifying as adverse events and serious adverse events

Abnormal laboratory findings (e.g., clinical chemistry, hematology, urinalysis) or other abnormal assessments (e.g. blood film) that are judged by the investigator to be clinically significant will be recorded as AEs or SAEs if they meet the definition of an AE, as defined in Section 8.1 or SAE, as defined in Section 8.2. Clinically significant abnormal laboratory findings or other abnormal assessments that are detected during the study or are present at baseline and significantly worsen following the start of the study will be reported as AEs or SAEs. However, clinically significant abnormal laboratory findings or other abnormal assessments that are associated with the disease being studied, unless judged by the investigator as more severe than expected for the subject's condition, or that are present or detected at the start of the study and do not worsen, will not be reported as AEs or SAEs.

The investigator will exercise his or her medical and scientific judgement in deciding whether an abnormal laboratory finding or other abnormal assessment is clinically significant.

8.4. Time period, frequency, and method of detecting adverse events and serious adverse events

All AEs occurring within 30 days following administration of each dose of vaccine must be recorded on the Adverse Event form in the subject's CRF, irrespective of severity or whether or not they are considered vaccination-related.

The standard time period for collecting and recording SAEs will begin at first receipt of vaccine and will end at the study conclusion. See Section 8.7 for instructions for reporting and recording SAEs.

Additionally, in order to fulfill international reporting obligations, SAEs that are related to study participation (e.g. procedures, invasive tests, a change from existing therapy) or are related to a concurrent medication will be collected and recorded from the time the subject consents to participate in the study until she/he is discharged: if a child is screened and enrolled, then discharge will be the end of the study as per study protocol; if a child is screened but not enrolled, then discharge will be the point at which the decision is taken not to enroll the child.

The investigator will inquire about the occurrence of AEs/SAEs at every visit/contact during the study and throughout the follow-up phase as appropriate.

The mechanism by which SAEs will be identified in the study are detailed in Section 5.1.14. The investigator or study clinician will fully document any such events on the Serious Adverse Event pages appended to the individual Case Report Form including, where applicable, information from relevant hospital case records, autopsy reports and verbal autopsies.

All AEs either observed by the investigator, study clinician, field worker or reported by the subject's parent/guardian spontaneously or in response to a direct question will be evaluated by the investigator. AEs not previously documented in the study will be recorded in the Adverse Event form within the subject's CRF. The nature of each event, date and time (where appropriate) of onset, outcome, intensity and relationship to vaccination will be established. Details of any corrective treatment will be recorded on the appropriate page of the CRF. Refer to Section 6.9.

As a consistent method of determining the occurrence of unsolicited AEs, the subject or the subject's parent/guardian will be asked a non-leading question such as:

'Has your child acted differently or felt different in any way since receiving the vaccine or since the last visit '

N.B. The investigator should record only those AEs having occurred within the time frame defined above.

AEs already documented in the CRF, i.e. at a previous assessment, and designated as 'not recovered/not resolved' or 'recovering/resolving' should be reviewed at subsequent visits, as necessary. If these have resolved, the documentation in the CRF should be completed.

N.B. If an AE changes in frequency or intensity during the specified reporting period, a new record of the event will be entered.

When an AE/SAE occurs, it is the responsibility of the investigator to review all documentation (e.g., hospital progress notes, laboratory, verbal autopsies and diagnostics reports) relative to the event. The investigator will then record all relevant information regarding an AE/SAE on the CRF or SAE Report Form as applicable. It is not acceptable for the investigator to send photocopies of the subject's medical records to GSK Biologicals in lieu of the appropriate completed AE/SAE pages. However, there may be instances when copies of medical records and verbal autopsies for certain cases are requested by GSK Biologicals. In this instance, all subject identifiers will be blinded on the copies of the medical records prior to submission to GSK Biologicals.

The investigator will attempt to establish a diagnosis of the event based on signs, symptoms, and/or other clinical information. In such cases, the diagnosis should be documented as the AE/SAE and not the individual signs/symptoms.

8.4.1. Solicited adverse events

Local (injection site) adverse events

- Pain at injection site
- Swelling at injection site

General adverse events

- Fever (defined as axillary temperature ≥ 37.5°C)
- Drowsiness
- Loss of appetite
- Irritability/fussiness

The visiting field worker will record these adverse events according to detailed SOPs available on study site during the field worker visits.

N.B. Temperature will be recorded on days 1 to 6 by the field worker or Principal Investigator. Should additional temperature measurements be performed at other times of day, the highest temperature will be recorded.

8.5. Evaluating adverse events and serious adverse events

8.5.1. Assessment of intensity

Intensity of the following AEs will be assessed as described:

Table 25 Intensity scales for solicited symptoms in infants/toddlers and children less than 6 years of age

Adverse Event	Intensity grade	Parameter
Pain at injection site	0	Absent
	1	Minor reaction to touch
	2	Cries/protests on touch
	3	Cries when limb is moved/spontaneously painful
Swelling at injection site		Record greatest surface diameter in mm
Fever		Record temperature in °C
Irritability/Fussiness	0	Behavior as usual
	1	Crying more than usual/ no effect on normal activity
	2	Crying more than usual/ interferes with normal activity
	3	Crying that cannot be comforted/ prevents normal activity
Drowsiness	0	Behavior as usual
	1	Drowsiness easily tolerated
	2	Drowsiness that interferes with normal activity
	3	Drowsiness that prevents normal activity
Loss of appetite	0	Appetite as usual
	1	Eating less than usual/ no effect on normal activity
	2	Eating less than usual/ interferes with normal activity
	3	Not eating at all

*Fever is defined as axillary temperature ≥ 37.5°C

The maximum intensity of local injection site swelling will be scored at GSK Biologicals as follows:

0 : None
1 : < 5 mm
2 : 5 to 20 mm
3 : > 20 mm

The maximum intensity of fever will be scored at GSK Biologicals as follows:

0 : < 37.5°C
1 : 37.5 – 38.0°C
2 : > 38 – 39.0°C
3 : > 39.0°C

The investigator will make an assessment of intensity for all other AEs, i.e. unsolicited symptoms, including SAEs reported during the study. The assessment will be based on the investigator's clinical judgement. The intensity of each AE and SAE recorded in the CRF or SAE Report Form, as applicable, should be assigned to one of the following categories:

1	=	An AE which is easily tolerated by the subject, causing minimal discomfort and not interfering with everyday activities.
2	=	An AE which is sufficiently discomforting to interfere with normal everyday activities.
3	=	An AE which prevents normal, everyday activities (in a young child, such an AE would, for example, prevent attendance at school/ kindergarten/ a day-care center and would cause the parents/ guardians to seek medical advice. In adults/ adolescents, such an AE would, for example, prevent attendance at work/ school and would necessitate the administration of corrective therapy).

An AE that is assessed as Grade 3 should not be confused with an SAE (serious adverse event). Grade 3 is a category utilized for rating the intensity of an event; and both AEs and SAEs can be assessed as Grade 3. An event is defined as 'serious' when it meets one of the pre-defined outcomes as described in Section 8.2.

8.5.2. Assessment of causality

The investigator is obligated to assess the relationship between investigational product and the occurrence of each AE/SAE. The investigator will use clinical judgement to determine the relationship. Alternative causes, such as natural history of the underlying diseases, concomitant therapy, other risk factors and the temporal relationship of the event to the investigational product will be considered and investigated. The investigator will also consult the Investigator Brochure and/or Product Information, for marketed products, in the determination of his/her assessment.

There may be situations when a SAE has occurred and the investigator has minimal information to include in the initial report to GSK Biologicals. However, it is very important that the investigator always makes an assessment of causality for every event prior to transmission of the SAE Report Form to GSK Biologicals. The investigator may change his/her opinion of causality in light of follow-up information, amending the SAE Report Form accordingly. The causality assessment is one of the criteria used when determining regulatory reporting requirements.

In case of concomitant administration of multiple vaccines, it may not be possible to determine the causal relationship of general AEs to the individual vaccines administered. The investigator should, therefore, assess whether the AE could be causally related to vaccination rather than to the individual vaccines.

All solicited local (injection site) reactions will be considered causally related to vaccination. Causality of all other AEs should be assessed by the investigator using the following question:

Is there a reasonable possibility that the AE may have been caused by the investigational product ?

NO	:	The AE is not causally related to administration of the study vaccine(s). There are other, more likely causes and administration of the study vaccine(s) is not suspected to have contributed to the AE.
YES	:	There is a reasonable possibility that the vaccine(s) contributed to the AE.

Non-serious and serious AEs will be evaluated as two distinct events. If an event meets the criteria to be determined “serious” (see Section 8.2 for definition of serious adverse event), it will be examined by the investigator to the extent to be able to determine ALL contributing factors applicable to each serious adverse event.

Other possible contributors include:

- Medical history
- Other medication
- Protocol required procedure
- Other procedure not required by the protocol
- Lack of efficacy of the vaccine(s), if applicable
- Erroneous administration
- Other cause (specify).

8.6. Follow-up of adverse events and serious adverse events and assessment of outcome

After the initial AE/SAE report, the investigator is required to proactively follow each subject and provide further information to GSK Biologicals on the subject’s condition.

All AEs and SAEs documented at a previous visit/contact and designated as not recovered/not resolved or recovering/resolving will be reviewed at subsequent visits/contacts.

Investigators will follow-up subjects:

- with SAEs or subjects withdrawn from the study as a result of an AE, until the event has resolved, subsided, stabilized, disappeared, the event is otherwise explained, or the subject is lost to follow-up;
- or, in the case of other non-serious AEs, until they complete the study or they are lost to follow-up.

Clinically significant laboratory abnormalities will be followed up until they have returned to normal, or a satisfactory explanation has been provided. Additional

information (including but not limited to laboratory results) relative to the subsequent course of such an abnormality noted for any subject must be made available to the Study Monitor.

GSK Biologicals may request that the investigator perform or arrange for the conduct of supplemental measurements and/or evaluations to elucidate as fully as possible the nature and/or causality of the AE or SAE. The investigator is obliged to assist. If a subject dies during participation in the study or during a recognized follow-up period, GSK Biologicals will be provided with a copy of any available post-mortem findings, including histopathology and verbal autopsies.

New or updated information will be recorded on the originally completed SAE Report Form, with all changes signed and dated by the investigator. The updated SAE report form should be resent to GSK Biologicals within 24 hours of receipt of the follow-up information as outlined in Section 8.7.1.

Outcome of any non-serious AE occurring within 30 days post-vaccination (i.e. unsolicited AE) or any SAE reported during the entire study will be assessed as:

- Recovered/resolved
- Not recovered/not resolved
- Recovering/resolving
- Recovered with sequelae/resolved with sequelae
- Fatal (SAEs only).

8.7. Prompt reporting of serious adverse events to GSK Biologicals

8.7.1. Time frames for submitting serious adverse event reports to GSK Biologicals

SAEs will be reported promptly to GSK and MVI at PATH once the investigator determines that the event meets the protocol definition of an SAE. The investigator or designate will fax or send electronically the SAE reports to GSK Biologicals' Study Contact for Serious Adverse Event Reporting **WITHIN 24 HOURS OF HIS/HER BECOMING AWARE OF THESE EVENTS**. Additional or follow-up information relating to the initial SAE report is also to be reported to the GSK Biologicals' Study Contact for Serious Adverse Event Reporting and the MVI at PATH contact within 24 hours of receipt of such information.

8.7.2. Completion and transmission of serious adverse event reports to GSK Biologicals

Once an investigator becomes aware that a SAE has occurred in a study subject, she/he will report the information to GSK and MVI at PATH within 24 hours as outlined in Section 8.7.1. The SAE Report Form will always be completed as thoroughly as possible

with all available details of the event, signed by the investigator (or designee), and forwarded to GSK and MVI at PATH within the designated time frames. If the investigator does not have all information regarding an SAE, he/she will not wait to receive additional information before notifying GSK and MVI at PATH of the event and completing the form. The form will be updated when additional information is received and forwarded to GSK and MVI at PATH **WITHIN 24 HOURS** as outlined in Section 8.7.1.

The investigator will always provide an assessment of causality at the time of the initial report as described in Section 8.5.2.

Facsimile (Fax) or electronic transmission of the SAE Report Form are the preferred methods to transmit this information to the Study Contact for Reporting SAEs. In rare circumstances and in the absence of facsimile equipment or electronic connection, notification by telephone is acceptable, with a copy of the SAE Report Form to follow. Initial notification via the telephone does not replace the need for the investigator to complete and sign the SAE Report Form within 24 hours as outlined in Section 8.7.1.

In the event of a death or SAE, determined by the investigator to be related to vaccination, sending of the fax or electronic report must be accompanied by telephone call to the Study Contact for Reporting SAEs.

Central Study Coordinator	Local GSK Study Monitor
Sabine Corachan,<br>Central Study Coordinator,<br>GlaxoSmithKline Biologicals,<br>Rue de l'Institut 89, 1330 Rixensart,<br>Belgium.<br>Tel: +32.2.656.92.33<br>Fax: +32.2.656.61.60<br>email: sabine.corachan@gskbio.com	~~Andreas Pakendorf &~~ Adelheid Davis,<br>GlaxoSmithKline,<br>Private Bag X173,<br>Bryanston 2021,<br>South Africa.<br>Tel: +27.11.745.63.24<br>Fax: +27.11.745.74.95<br>email: ~~andreas.m.pakendorf@gsk.com &~~<br>adelheid.a.davis@gsk.com<br><br>***(Amended 29 June 2005)***

Malaria Vaccine Initiative

Filip Dubovsky,<br>6290 Montrose Road, Rockville,<br>Maryland 20852,<br>USA.<br>Tel: +1.301.770.53.77<br>Fax: +1.301.770.53.22<br>email: sae@malariavaccine.org<br><br>*alternate email:*<br>fdubovsky@malariavaccine.org	Jessica Milman,<br>6290 Montrose Road, Rockville,<br>Maryland 20852,<br>USA.<br>Tel: +1.301.770.53.77<br>Fax: +1.301.770.53.22<br>email: sae@malariavaccine.org<br><br>*alternate email:*<br>jmilman@malariavaccine.org

**Back-up Study Contact at GSK Biologicals for Reporting Serious Adverse Events
Manager Clinical Safety Vaccines**

GSK Biologicals Clinical Safety Physician,
GlaxoSmithKline Biologicals,
Rue de l'Institut 89, 1330 Rixensart,
Belgium.
Tel: +32.2.656.87.98
Fax: +32.2.656.80.09
Mobile phone for 7/7 day availability: +32.477.40.47.13

8.8. Regulatory reporting requirements for serious adverse events

The investigator will promptly report all SAEs to GSK in accordance with the procedures detailed in Section 8.7. GSK Biologicals has a legal responsibility to promptly notify, as appropriate, both the local regulatory authority and other regulatory agencies about the safety of a product under clinical investigation. Prompt notification of SAEs by the investigator to the Study Contact for Reporting SAEs is essential so that legal obligations and ethical responsibilities towards the safety of other subjects are met.

The investigator, or responsible person according to local requirements, will comply with the applicable local regulatory requirements related to the reporting of SAEs to regulatory authorities and the IRB/IEC.

This protocol has been filed under an Investigational New Drug (IND) application with the US Food and Drug Administration (FDA). A given SAE may qualify as an IND Safety Report if the SAE is both attributable to the investigational product and unexpected. In this case, all investigators filed to the IND (and associated INDs for the same compound) will receive an Expedited Investigator Safety Report (EISR), identical in content to the IND Safety Report submitted to the FDA.

EISRs are prepared according to GSK Biologicals' policy and are forwarded to investigators as necessary. An EISR is required for:

- development compounds (i.e. compounds not marketed), if the event is serious, unexpected and has a suspected relationship to study drug treatment. Expected adverse events for development compounds will be described in the Development Core Safety Information (DCSI) in the Investigator Brochure (IB).
- marketed compounds (i.e. approved in at least one market), if the event is serious, unexpected and has a suspected relationship to treatment with a GSK product AND is a significant new emerging safety issue. Expected adverse events for marketed compounds will be described in the Core Safety Information (CSI). An EISR is required if an SAE was expedited to the IND in the US or to fulfill regulatory obligations in other countries. An EISR for Post marketing surveillance (PMS)/Phase IV studies would not typically be required.

The purpose of the EISR is to fulfill specific regulatory and Good Clinical Practice (GCP) requirements, regarding the product under investigation.

When a site receives an Initial or Follow-up EISR or other safety information (e.g., revised Investigator Brochure) from GSK Biologicals, the responsible person according to local requirements is required to promptly notify his or her IRB/IEC.

8.9. Post study adverse events and serious adverse events

A post-study AE/SAE is defined as any event that occurs outside of the AE/SAE detection period defined in Section 8.4. Investigators are not obligated to actively seek AEs or SAEs in former study participants.

However, if the investigator learns of any SAE, including a death, at any time after a subject has been discharged from the study, and he/she considers the event reasonably related to the investigational product, the investigator will promptly notify the Study Contact for Reporting SAEs.

8.10. Pregnancy

Not applicable

8.11. Treatment of adverse events

Treatment of any adverse event is at the sole discretion of the investigator and according to current good medical practice. Any medication administered for the treatment of an AE should be recorded in the subject's CRF according to Section 6.9.

9. SUBJECT COMPLETION AND WITHDRAWAL

9.1. Subject completion

A subject who returns for the concluding visit/is available for the concluding contact foreseen in the protocol is considered to have completed the study.

A subject that comes for Clinic Visit 11 has completed the double-blind phase of the study. A subject that comes for Clinic Visit 12 has completed the single-blind phase of the study.

9.2. Subject withdrawal

The parent(s) or guardian(s) of subjects that are withdrawn or withdraw prior to completion of a 3-dose course of TETRActHib will be advised to complete their child's routine infant immunization with the EPI services.

Subjects who are withdrawn for AEs must be clearly distinguished from subjects who are withdrawn for other reasons. Investigators will follow subjects who are withdrawn as result of a SAE/AE until resolution of the event (see Section 8.6). Withdrawals will not be replaced.

9.2.1. Subject withdrawal from the study

From an analysis perspective, a 'withdrawal' from the study is any subject who was not available for the concluding contact foreseen in the protocol. A subject that comes for Clinic Visit 11 has completed the double-blind phase of the study. A subject that comes for Clinic Visit 12 has completed the single-blind phase of the study.

A subject qualifies as a 'withdrawal' from the study when no study procedure has occurred, no follow-up has been performed and no further information has been collected for this subject from the date of withdrawal/last contact.

Investigators will make an attempt to contact those subjects who do not return for scheduled visits or follow-up.

Information relative to the withdrawal will be documented on the Study Conclusion page of the CRF. The investigator will document whether the decision to withdraw from the study was made by the subject's parent or guardian or the investigator and which of the following possible reasons was responsible for withdrawal:

- serious adverse event
- non-serious adverse event
- protocol violation (specify)
- consent withdrawal, not due to an adverse event
- moved from the study area
- lost to follow-up
- other (specify).

9.2.2. Subject withdrawal from investigational product

A ‘withdrawal’ from the investigational product is any subject who does not receive the complete treatment, i.e. when no further planned dose is administered from the date of withdrawal. A subject withdrawn from the investigational product may not necessarily be withdrawn from the study as further study procedures or follow-up may be performed (safety or immunogenicity) if planned in the study protocol.

Information relative to premature discontinuation of the investigational product will be documented on the Vaccine Administration page of the CRF. The investigator will document whether the decision to discontinue further vaccination/treatment was made by the subject’s parent or guardian or the investigator and which of the following possible reasons was responsible for withdrawal:

- serious adverse event;
- non-serious adverse event;
- other (specify).

10. DATA EVALUATION: CRITERIA FOR EVALUATION OF OBJECTIVES

10.1. Primary Endpoints

10.1.1. Primary endpoint; safety

- Occurrence of SAEs from the time of first TETRActHib vaccination until months 6 post Dose 1

10.2. Secondary endpoints

10.2.1. Secondary endpoints for Safety

- Occurrence of unsolicited AEs after Dose 1, 2 and 3 of TETRActHib over a 14 day follow-up period (day of vaccination and 13 subsequent days)

- Occurrence of unsolicited AEs after Dose 1 and 2 of RTS,S/AS02D or Engerix-B over a 14 day follow-up period (day of vaccination and 13 subsequent days)
- Occurrence of unsolicited AEs after Dose 3 of RTS,S/AS02D or Engerix-B over a 30 day follow-up period (day of vaccination and 29 subsequent days)
- Occurrence of solicited general and local reactions over a 7 day follow-up period (day of vaccination and 6 subsequent days) after TETRActHib
- Occurrence of solicited general and local reactions over a 7 day follow-up period (day of vaccination and 6 subsequent days) after RTS,S/AS02D or Engerix-B.

10.2.2. Secondary endpoints for Immunogenicity

Endpoints assessed one month post Dose 3 of RTS,S/AS02D or Engerix-B; assessment of non-inferiority:

- Anti-HBs antibody titers; difference between groups in percent seroprotection <10%

Endpoints assessed prior to vaccination, 1 month post Dose 3 of RTS,S/AS02D or Engerix-B:

- Anti HBs antibody titers

Endpoints assessed prior to vaccination, 1 month post Dose 3, 3½ months post Dose 3 of RTS,S/AS02D or Engerix-B:

- Anti-CS antibody titers.

Endpoints assessed 1 month post Dose 3 of TETRActHib:

- Anti-diphtheria antibody titers measured by ELISA
- Anti-tetanus antibody titers measured by ELISA
- Anti-BPT antibody titers measured by ELISA
- Anti-PRP antibody titers measured by ELISA

10.2.3. Secondary endpoints for Proof of concept

- The time to first malaria infection (first recording of infection of asexual stage falciparum parasites detected by the active detection of infection surveillance) over a period starting 14 days after last vaccination with RTS,S/AS02D extending for 12 weeks.
- The asexual *P. falciparum* parasitemia (prevalence and density) at 3½ months post Dose 3 (Month 6).

10.3. Tertiary Endpoints

10.3.1. Tertiary endpoints for Safety

- Occurrence of serious adverse events during the single-blind surveillance period (from Month 7 to Month 14 inclusive).

10.3.2. Tertiary endpoints for Immunogenicity

Endpoints assessed 12 months post Dose 3:

- Anti HBsAg antibody titers
- Anti-CS antibody titers.

10.4. Exploratory Endpoints

From Day 0 and extending to 3½ months post Dose 3 of Engerix-B or RTS,S/AS02D

- The time to the first clinical episode of symptomatic *P. falciparum* malaria (defined as the presence of *P. falciparum* asexual parasitemia above 500 per µL on Giemsa stained thick blood films AND the presence of fever [axillary temperature ≥ 37.5°C] at the time of presentation) detected by passive case detection.

From Day 0 and extending to 3½ months post Dose 3 of Engerix-B or RTS,S/AS02D

- The time to the first clinical episode of symptomatic *P. falciparum* malaria (defined as the presence of *P. falciparum* asexual parasitemia above 500 per µL on Giemsa stained thick blood films AND the presence of fever [axillary temperature ≥ 37.5°C] at the time of presentation) detected by passive case detection and active case detection.

From Day 0 and extending to 14 months post Dose 3 of Engerix-B or RTS,S/AS02D

- The time to the first clinical episode of symptomatic *P. falciparum* malaria (defined as the presence of *P. falciparum* asexual parasitemia above 500 per µL on Giemsa stained thick blood films AND the presence of fever [axillary temperature ≥ 37.5°C] at the time of presentation) detected by passive case detection.

- The total number of clinical episodes of symptomatic *P. falciparum* malaria (defined as the presence of *P. falciparum* asexual parasitemia above 500 per µL on Giemsa stained thick blood films AND the presence of fever [axillary temperature ≥ 37.5°C] at the time of presentation) detected by passive case detection.

Endpoint assessed at screening, 1 month post Dose 3 and 3½ months post Dose 3 of Engerix-B or RTS,S/AS02D

- The frequency of CS-specific CD4+ and CD8+ T cells, as measured by intracellular cytokine staining.

10.5. Estimated sample size

In the study area there are approximately 30 births per month. We have assumed that 25% of children will not meet eligibility criteria; given the previous experience at the site and the estimated rates of Hepatitis B chronic carriage and HIV prevalence in antenatal women. A loss to follow up rate of 10% is anticipated and therefore 220 subjects will be enrolled to provide 200 evaluable subjects at study conclusion.

10.5.1. Safety

This is the first administration of RTS,S/AS02D to infants and therefore the sample size of this Phase 1/2 safety study is limited to 200. Therefore only large differences in the frequencies of SAEs can be detected with reasonable power. The data set will be examined, comparing the rates of SAEs at the Medical Dictionary for Regulatory Activities (MedDRA) preferred term level. A sample size of 200 has the power to detect differences in the rates of safety endpoints (SAEs at the MedDRA preferred term level) as shown in Table 26. All calculations were performed with PASS 2000, using an asymptotic test for difference in proportions, type I error rate of 5% and no correction for multiplicity.

Table 26 Differences in safety endpoints (SAEs at the MedDRA preferred term level) between treatment groups that can be detected with 80% power for varying baseline rates

Frequency of events for Engerix-B	Frequency of events for RTS,S/AS02D	Power to detect difference (number per group equals 100)
1%	12%	80%
3%	15%	80%
5%	19%	80%
10%	26%	80%

10.5.2. Sample size for secondary endpoint immunology of Hepatitis B response

This study will compare the immunogenicity of RTS,S/AS02D to Engerix-B. A non-inferiority approach will be used to calculate the power of the study for the comparison of seroprotection rates and geometric mean titers of anti-HBs antibodies. The assessment of the anti-HBs antibody immune response will be based on approximately 200 evaluable subjects (100 subjects per group). This sample size will have 90% power at a 2.5% significance level using a one-sided equivalence test of proportions when the proportion that are seroprotected in the control group (subjects vaccinated with Engerix-B) is at least 0.97 and the proportion in the RTS,S/AS02D group is 0.97 and the maximum allowable difference between these proportions that still results in equivalence (the range of equivalence) is 0.10 (PASS 2000, one sided equivalence test of proportions, alpha = 2.5%).

Similarly this sample size will provide 98.5% power to demonstrate at a 5% significance level using an equivalence of means test, that the lower limit of a two sided 95% confidence interval on the GMT ratio of anti-HBs antibodies (RTS,S/AS02D vaccine

group over Engerix-B vaccine group) is greater than 0.5 (2 fold decrease). (PASS 2000, equivalence of means, alpha = 5%). The standard deviation used for this calculation was 0.52 (computed on log base 10 transformed titers) and was derived from a GSK Biologicals study (Malaria-034) on 67 subjects which received three doses of RTS,S/AS02D administered at a 0, 1, 2 months schedule.

10.5.3. Sample size for secondary endpoint proof of concept

Assuming an attack rate of 75% in the Control Group over the 3-month surveillance period (Personal Communication JJ Aponte September 2004: data from a study performed in Ilha Josina in 2001, July to September in children 7 to 18 months), 100 evaluable subjects per group are needed in order to have 90% power to show a Vaccine Efficacy (=1-hazard ratio) of 45% (Pass 2000, Log rank Survival, alpha=0.05, based on the assumption that the hazard rates are proportional). This is equivalent to an attack rate in the Vaccinated Group of 53.3%. Using proportion of subjects with malaria infection over the surveillance period, a Vaccine Efficacy (=1-risk ratio) of 29% can be detected with 90% power.

10.6. Study cohorts to be evaluated

10.6.1. Total Vaccinated Cohort

The Total Vaccinated Cohort will include all vaccinated subjects for whom data are available. Thus, the total analysis of safety will include all subjects with at least one vaccine administration documented and the total analysis of immunogenicity/efficacy will include vaccinated subjects for whom data concerning immunogenicity/efficacy endpoint measures are available. The Total Vaccinated Cohort analysis will be performed per treatment actually administered.

10.6.2. According to protocol (ATP) cohort for analysis of safety

The ATP cohort for analysis of safety will include all evaluable subjects;

- who have received at least one dose of study vaccine according to their random assignment
- have sufficient data to perform an analysis of safety (at least one vaccine dose with safety follow-up)
- for whom administration site of study vaccine is known
- who have not received a vaccine not specified or forbidden in the protocol and for whom elimination criteria were not applied
- for whom the randomization code has not been broken except for when unblinding has been carried out by the DSMB for Safety Analysis.
- who meet all eligibility criteria.

10.6.3. According to protocol (ATP) cohort for analysis of immunogenicity

The ATP cohort for analysis of immunogenicity will include all evaluable subjects (i.e. those meeting all eligibility criteria, complying with the procedures defined in the protocol, with no elimination criteria during the study) for whom data concerning immunogenicity endpoint measures are available.

10.6.4. According to protocol (ATP) cohort for analysis of efficacy

The ATP cohort for analysis of efficacy will include all evaluable subjects (i.e. those meeting all eligibility criteria, complying with the procedures defined in the protocol, with no elimination criteria during the study) for whom data concerning efficacy endpoint measures are available.

10.7. Derived and transformed data

- Seroprotection rate for HBsAg is defined as the percentage of subjects with antibody titers greater than or equal to an established cut-off (anti-HBsAg ≥ 10 mIU/mL).
- Seroprotection rate for Diphtheria is defined as the percentage of subjects with antibody titers greater than or equal to an established cut-off (anti-diphtheria ≥ 0.1 IU/mL).
- Seroprotection rate for Tetanus is defined as the percentage of subjects with antibody titers greater than or equal to an established cut-off (anti-tetanus ≥ 0.1 IU/mL).
- Seroprotection rate for PRP is defined as the percentage of subjects with antibody titers greater than or equal to an established cut-off (anti-PRP ≥ 0.15 µg/mL).
- A subject seropositive for anti-BPT antibody is a subject whose antibody titer is greater than or equal to the cut-off value (anti-BPT ≥ 15 EL.U/mL).
- A subject seropositive for anti-CS antibody is a subject whose antibody titer is greater than or equal to the cut-off value (anti-CS ≥ 0.5 EU/mL).
- Vaccine response is defined as follows:
  - The appearance of antibodies one month post Dose 3 (titer ≥ cut-off) in initially seronegative subjects (seroconversion)
  - Post vaccination antibody titers ≥ pre-vaccination titers in initially sero-positive subjects
- The Geometric Mean Titers (GMTs) calculations are performed by taking the anti-log of the mean of the log10 titer transformations. Antibody titers below the cut-off of the assay will be given an arbitrary value of half the cut-off for the purpose of GMT calculation.
- The Geometric Mean Concentration (GMC) calculations are performed by taking the anti-log of the mean of the log10 concentration transformations. Antibody concentrations below the cut-off of the assay will be given an arbitrary value of half the cut-off for the purpose of GMC calculation.

For a given subject and a given immunogenicity measurement, missing or non-evaluable measurements will not be replaced. Therefore, an analysis will exclude subjects with missing or non-evaluable measurements.

For the analysis of solicited local/general symptoms, only subjects for which information on the solicited local/general symptom sheet is available for the considered study dose will be included in the analysis.

10.8. Planned Interim Analysis

One interim analysis is planned at one month post Dose 3 of RTS,S/AS02D or Engerix-B, Clinic Visit 10. The interim analysis will be performed by the sponsor. In order to maintain blinding, the analyses will be performed by a statistician independent of the GSK Biologicals clinical teams. The interim analysis will be done on cleaned data and will contain analyses of reactogenicity, safety and immunogenicity.

This interim analysis will contain the final analyses for the reactogenicity (local and general solicited symptoms within 7 days of vaccination and unsolicited symptoms within 14 or 30 days of vaccination) and immunogenicity on Clinic Visit 1, 9 and 10.

10.8.1. Analysis of demographics/baseline characteristics

Demographic characteristics (age, gender) of each study cohort will be tabulated.

The mean age (plus range and standard deviation) by gender of the enrolled subjects, as a whole, and per group, will be calculated.

The distribution of subjects enrolled will be tabulated as a whole and per group.

10.8.2. Analyses of safety

Safety will be analyzed on the Total Cohort and the ATP cohort for Safety (for the purpose of reporting local reactogenicity the ATP will be used to ensure that those subjects who received vaccines in the wrong limb are not included).

The analysis of the occurrence of SAEs will be performed on the Total Vaccinated Cohort. The proportion of subjects with an SAE (until Clinic Visit 10), classified by the MedDRA preferred term level, reported from study start until study conclusion will be tabulated with exact 95% CI. Comparisons between groups will be done using Fisher's Exact test for each preferred term.

Serious adverse events and withdrawal due to adverse event(s), from study start until Clinic Visit 10 will be described in detail.

The percentage of subjects with at least one local adverse event (solicited and unsolicited), with at least one general adverse event (solicited and unsolicited) and with any adverse event during the solicited follow-up period will be tabulated with exact 95% CI after each vaccine dose and overall. The percentage of doses followed by at least one

local adverse event (solicited and unsolicited), by at least one general adverse event (solicited and unsolicited) and by any adverse event will be tabulated, overall vaccination course, with exact 95% CI.

The percentage of subjects reporting each individual solicited local and general adverse event during the solicited follow-up period will be tabulated with exact 95% CI. The percentage of doses followed by each individual solicited local and general adverse event will be tabulated, overall vaccination course, with exact 95% CI.

For all other solicited symptoms, the same tabulation will be performed for Grade 3 adverse events and for adverse events with relationship to vaccination.

For seizures occurring within 7 days of vaccination, an analysis will be performed based on the Brighton Collaborations guidelines [Bonhoeffer, 2004]. This includes descriptive tables of the time relationship of seizures to vaccination, the duration of seizures, the level of certainty and the relationship to episodes of fever.

The proportion of subjects with at least one report of unsolicited adverse event classified by the Medical Dictionary for Regulatory Activities (MedDRA), whenever available, and reported up to 30 days after vaccination (14 days after Dose 1, 2 and 3 of TETRActHib and after Dose 1 and 2 of RTS,S/AS02D or Engerix-B) will be tabulated with exact 95% CI. The same tabulation will be performed for Grade 3 unsolicited adverse events and for unsolicited adverse events with a relationship to vaccination.

Biochemistry (ALT, bilirubin, creatinine) and hematology (hemoglobin, WBC, platelets) values that are outside of the reference ranges will be described for timepoints Clinic Visit 1, 4 and 10.

10.8.3. Analyses of immunogenicity

The primary analysis will be based on the ATP cohort for analysis of immunogenicity. If the percent of enrolled subjects excluded from this ATP cohort is more than 5%, a second analysis based on the Total Vaccinated cohort will be performed to complement the ATP analysis.

10.8.3.1. Assessment of non-inferiority Clinic Visit 10 (one month post Dose 3 of RTS,S/AS02D or Engerix-B)

For seroprotection rates (anti-HBsAg), the difference between both groups (RTS,S/AS02D – Engerix-B) will be calculated as well as the 95% CI around this difference. If the lower limit of the two-sided 95% CI is greater than –0.10, non-inferiority of RTS,S/AS02D compared to Engerix-B will have been demonstrated in this study.

10.8.3.2. Anti-HBs antibodies

The seroprotective level for anti-HBs is ≥ 10 mIU/mL; seroprotective rates will be used in the assessment of non-inferiority at 1 month post Dose 3. The percentage of subjects

with protective levels of anti-HBs (≥ 10 mIU/mL) with 95% confidence interval (95% CI) will be determined at each blood sampling time point (Screening and Clinic Visit 10). Antibody titers will be summarized by GMT with 95% CI at all time points at which serological samples are taken (Screening and Clinic Visit 10). Antibody titers after the third dose will also be investigated using reverse cumulative curves.

10.8.3.3. Anti-CS antibodies

The percentage of subjects with sero-positive levels of anti-CS (proportion of subjects with anti-CS antibody titers greater than or equal to 0.5 EU/mL) with 95% CI will be determined at each blood sampling time point (Screening and Clinic Visit 10). Antibody titers will be summarized by GMT with 95% CI at all time points at which serological samples are taken. Antibody titers after the third dose will also be investigated using reverse cumulative curves.

10.8.3.4. Anti-BPT antibodies

Antibody titers will be summarized by GMT with 95% CI at Clinic Visit 9 (one month post Dose 3 of TETRActHib) The percentage of subjects with anti-*Bordetella pertussis* titer ≥ the assay cut off of 15 EL.U/mL with 95% CI will be calculated. Antibody titers after the third dose will also be investigated using reverse cumulative curves.

10.8.3.5. Anti-diphtheria toxoid antibodies

The seroprotective level for anti-diphtheria is ≥ 0.1 IU/mL. The percentage of subjects with protective levels of anti-diphtheria antibodies with 95% CI will be determined at Clinic Visit 9 (one month post Dose 3 of TETRActHib). Antibody titers will be summarized by GMT with 95% CI. Antibody titers after the third dose will also be investigated using reverse cumulative curves.

10.8.3.6. Anti-tetanus toxoid antibodies

The seroprotective level for anti-tetanus is ≥ 0.1 IU/mL. The percentage of subjects with protective levels of anti-tetanus antibodies with 95% CI will be determined at Clinic Visit 9 (one month post Dose 3 of TETRActHib). Antibody titers will be summarized by GMT with 95% CI. Antibody titers after the third dose will also be investigated using reverse cumulative curves.

10.8.3.7. Anti-PRP antibodies

The seroprotective level for anti-PRP is ≥ 0.15 µg/mL. The percentage of subjects with protective levels of anti-PRP antibodies with 95% CI will be determined at Clinic Visit 9 (one month post Dose 3 of TETRActHib). Antibody titers will be summarized by GMT with 95% CI. The proportion of subjects with titers > 1.0 µg/mL with 95% CI will be calculated. Antibody titers after the third dose will also be investigated using reverse cumulative curves.

10.9. Final Analyses

The final analysis will complement the interim analysis to provide complete analysis on all primary and secondary endpoints.

10.9.1. Analyses of safety

10.9.1.1. Primary endpoint

For the safety primary endpoint, the occurrence of SAEs will be performed on the Total Vaccinated Cohort. The proportion of subjects with an SAE, classified by the MedDRA preferred term level, reported from study start until study conclusion (end of the double-blind phase) will be tabulated with exact 95% CI. Comparisons between groups will be done using Fisher's Exact test for each preferred term. If the percent of enrolled subjects excluded from the ATP cohort for analysis of safety is more than 5%, a second analysis based on this ATP cohort will be performed to complement the Total Analysis.

10.9.1.2. Other safety endpoints

Serious adverse events and withdrawal due to adverse event(s) will be described in detail.

Biochemistry (ALT, bilirubin, creatinine) and hematology (hemoglobin, WBC, platelets) values that are outside of the reference ranges will be described for Clinic Visit 11 (end of double-blind phase).

10.9.2. Analyses of immunogenicity

The primary analysis will be based on the ATP cohort for analysis of immunogenicity. If the percent of enrolled subjects excluded from this ATP cohort is more than 5%, a second analysis based on the Total Vaccinated cohort will be performed to complement the ATP analysis.

10.9.2.1. Anti-CS antibodies

The percentage of subjects with sero-positive levels of anti-CS (proportion of subjects with anti-CS antibody titers greater than or equal to 0.5 EU/mL) with 95% CI will be determined at Month 6 (Clinic Visit 11). Antibody titers will be summarized by GMT with 95%CI. Antibody titers after the third dose will also be investigated using reverse cumulative curves.

10.9.3. Analyses of efficacy

10.9.3.1. Calculation of the time at risk

For the efficacy endpoint (time to first malaria infection), the time at risk will be counted in days, and expressed as child years at risk (days/365.25).

For each subject in the Total Cohort, time at risk begins 14 days after Dose 3 for those that received Dose 3 or 2½ months after Dose 1 (Day ~~75~~ ***90***) otherwise. For the According to Protocol cohort, time at risk begins 14 days after Dose 3 ***(Amended 12 April 2005).***

The time at risk will end whenever one of the following conditions happen first: fits the case definition for malaria infection, loss of follow up, emigration from the study area, withdrawal, death, end of follow-up period (Clinic Visit 11).

The time at risk will take into account absences from the study area and anti-malaria drug therapy. The monthly home visits will be used to determine any period of travel outside the study area. Absences from the study area of 2 weeks or more will be recorded in multiples of 1 week. The date of departure will be documented. Similarly, if treatment for malaria is administered, the subject will not be considered susceptible to malaria infection for the longest duration of the combination of drugs the subject could receive for this episode as follows: 28 days if received sulphadoxine-pyrimethamine, 7 days if having received chloroquine alone, 7 days if having received quinine alone, 20 days if having received co-artemesinin.

If an episode is detected during a period of time not counting for the time at risk it will not be included in the analysis, however a table will be presented showing the number of such episodes not included in the analysis.

Episodes will be included in the analysis only if the child is identified properly showing his/her ID Card at the contact with the health facility and the study number is written in the OPD form. Only blood slides that complete the reading process according to SOP will be considered in the establishment of a malaria infection.

10.9.3.2. Vaccine efficacy

Time to first infection of *P. falciparum* will be examined using Kaplan-Meier curves for both groups. The distribution will be compared with the Wilcoxon test (if efficacy varies with time) or the Log-rank test (if it does not). Vaccine Efficacy will be assessed using Cox regression models. Vaccine efficacy is defined as 1-R where R is the hazard ratio of the RTS,S/AS02D group versus the Engerix-B group (with 95% CI). Crude and adjusted estimates for prognostic/stratifying factors (see Section 10.9.3.3) will be presented.

Cox regression assumes proportional hazards throughout the follow-up period. This assumption will be checked by plotting by group the log of the cumulative hazard against the log of time. Under the assumption of proportionality of the hazard, both curves should be parallel. A test based on the Schoenfield residuals will be performed.

If there is strong evidence that the hazard is not constant over the surveillance period, then alternative approaches to analyze the data will be examined according to the following:

- Cox regression models with time varying covariates, using fractional polynomials to identify the function of the time that best fits. The following functions of the time will

be explored: –2, -1, -½, log, identity, ½, 2, 3. The Akaike's criterion and the Schwarz Bayesian criterion will be explored.

- Piecewise Cox regression model, partitioning the surveillance period in three periods based on the equal number of events

For the assessment of the parasite density at Month 6, a 2x2 table presenting the frequency of positive and negative will be presented. The effect of the group will be evaluated using the Fisher exact test. The geometric mean of those positive in each group will be calculated. Difference in geometric mean and 95% CI will be estimated. The effect of the group will be evaluated using the t-test. In case the assumptions of the t-test are not satisfied (normality of residuals and equal variance) a non-parametric Wilcoxon test will be used instead.

10.9.3.3. Adjustment for covariates

The risk for malaria infection and development of clinical disease depends on numerous factors related to the parasite, host and vector biology. Estimates of the vaccine efficacy will be made, firstly based on treatment group and secondly adjusted for:

- age: continuous variable.
- geographical area ('barrios' local government areas): categorical variable.
- distance from health center (in kilometers): continuous variable.

An exploratory examination of the marginal effect each of the above covariates have on the treatment estimate may also be undertaken.

10.9.4. Exploratory analysis

The exploratory endpoints will be analyzed in the Total Vaccinated Cohort and the ATP Cohort for efficacy.

For the first exploratory endpoint (time to the first clinical episode of symptomatic *P. falciparum* malaria defined as the presence of *P. falciparum* asexual parasitemia above 500 per µL on Giemsa stained thick blood films AND the presence of fever [axillary temperature ≥ 37.5°C] at the time of presentation), the time at risk is defined as in Section 10.9.3.1. However, the time at risk will begin at Day 0 and no adjustments will be made for the absences from the study area and the anti-malarial drug therapy. Vaccine efficacy will be calculated in the same manner as described in Section 10.9.3.2 using Cox regression models. Only crude estimates, not adjusted for prognostic/stratifying factors will be presented.

This analysis will be done including cases from the passive case detection only (see Section 5.1.16) and including cases from both the passive case detection and the active detection of infection (ADI, see Section 5.1.17)

Note that the total number of clinical episodes will only be analyzed at the end of the single-blind phase.

The frequency of CS-specific CD4+ and CD8+ T cells will be summarized for each group at each timepoint (descriptive statistics).

10.10. Annex analysis at study conclusion

10.10.1. Analyses of safety

The analysis of the occurrence of SAEs will be performed on the Total Vaccinated Cohort. The proportion of subjects with an SAE, classified by the MedDRA preferred term level, reported from study start until study conclusion (end of the single-blind phase) will be tabulated with exact 95% CI. Comparisons between groups will be done using Fisher's Exact test for each preferred term.

Serious adverse events and withdrawal due to adverse event(s) will be described in detail.

Biochemistry (ALT, bilirubin, creatinine) and hematology (hemoglobin, WBC, platelets) values that are outside of the reference ranges will be described for Clinic Visit 12 (end of single-blind phase).

10.10.2. Analyses of immunogenicity

The primary analysis will be based on the ATP cohort for analysis of immunogenicity. If the percent of enrolled subjects excluded from this ATP cohort is more than 5%, a second analysis based on the Total Vaccinated cohort will be performed to complement the ATP analysis.

10.10.2.1. Anti-HBs antibodies

The seroprotective level for anti-HBs is ≥ 10 mIU/mL; The percentage of subjects with protective levels of anti-HBs (≥ 10 mIU/mL) with 95% confidence interval (95% CI) will be determined at Month 14 (Clinic Visit 12). Antibody titers will be summarized by GMT with 95% CI at Month 14 (Clinic Visit 12). Antibody titers after the third dose will also be investigated using reverse cumulative curves.

10.10.2.2. Anti-CS antibodies

The percentage of subjects with sero-positive levels of anti-CS (proportion of subjects with anti-CS antibody titers greater than or equal to 0.5 EU/mL) with 95% CI will be determined at Clinic Visit 12. Antibody titers will be summarized by GMT with 95%CI. Antibody titers after the third dose will also be investigated using reverse cumulative curves.

10.10.3. Analyses of efficacy

10.10.3.1. Exploratory analysis

The exploratory endpoints will be analyzed in the Total Vaccinated Cohort and the ATP Cohort for efficacy.

For the first exploratory endpoint (time to the first clinical episode of symptomatic *P. falciparum* malaria defined as the presence of *P. falciparum* asexual parasitemia above 500 per µL on Giemsa stained thick blood films AND the presence of fever [axillary temperature ≥ 37.5°C] at the time of presentation), the time at risk is defined as in Section 10.9.3.1. However, the time at risk will begin at Day 0 and will end whenever one of the following conditions happen first: fits the case definition for malaria infection, loss of follow up, emigration from the study area, withdrawal, death, end of follow-up period (Clinic Visit 12, end of single blind phase). The time at risk will not be adjusted for the absences from the study area and the anti-malarial drug therapy. Vaccine efficacy will be calculated in the same manner as described in Section 10.9.3.2 using Cox regression models. Only crude estimates, not adjusted for prognostic/stratifying factors will be presented.

For the analysis of the total number of clinical episodes, the incidence of episodes (total number of episodes/person years at risk) for each group will be presented. The significance of the group effect will be assessed using Poisson regression models with random effects including the time at risk as an off-set variable. Vaccine efficacy is defined as 1-R where R is the rate ratio of clinical episodes of the RTS,S/AS02D group versus the Engerix-B group (with 95% CI). Only crude estimates, not adjusted for prognostic/stratifying factors will be presented.

Both analyses will be done including cases from the passive case detection only (see Section 5.1.16).

11. ADMINISTRATIVE MATTERS

To comply with Good Clinical Practice important administrative obligations relating to investigator responsibilities, monitoring, archiving data, audits, confidentiality and publications must be fulfilled. See Appendix B for details.

12. REFERENCES

Alonso PL, Sacarlal J, Aponte JJ, Leach A, Macete E et al. Efficacy of the RTS,S/AS02A vaccine against *Plasmodium falciparum* infection and disease in young African children: randomised controlled trial. *The Lancet.* 2004;364:1411-1420.

Bojang KA, Milligan PJM, Pinder M, Vigneron L, Alloueche A, Kester KE et al. Efficacy of RTS,S/AS02 Malaria Vaccine against *Plasmodium falciparum* Infection in Semi-Immune Adult Men in the Gambia: A Randomized Trial. *The Lancet.* 2001;358: 1927-34.

Bonhoeffer J, Menkes J, Gold, MS, de Souza-Brito G, Fisher MC et al. Generalized convulsive seizure as an adverse event following immunization: case definition and guidelines for data collection, analysis and presentation. *Vaccine*. 2004; 22: 557-562.

Breman JG, Egan A, Keusch GT. Introduction and Summary. The Intolerable Burden of Malaria: A New Look at the Numbers. *Am J Trop Med Hyg*. 2001;64 (1, 2)S: iv-vii.

Breman, JG. The Ears of the Hippopotamus: Manifestations, Determinants and Estimates of the Malaria Burden. *Am J Trop Med Hyg.* 2001; 64:1-11.

Camargo ME, et al. Immunoenzymatic assay of anti-diphtheric toxin antibodies in human serum. *J Clin Microbiol* 1984; 20 (4): 772-774.

Center for Disease Control. National Health and Nutrition Examination Survey. http://www.cdc.gov/nchs/about/major/nhanes/growthcharts/zscore/zscore.htm. Accessed 17 January 2005 ***(Amended 17 January 2005)***.

Doherty JF, Pinder M, Tornieporth N, Carton C, Vigneron L, Milligan P et al. A Phase I Safety and Immunogenicity Trial with the Candidate Malaria Vaccine RTS,S.SBAS02 in Semi-Immune Adults in The Gambia. *J Am Soc Trop Med Hyg*. 1999;61 (6): 865-68.

Fidel F, Quinto L, Masanja H, Nathan R, Ascaso C, et al. Pediatric referrals in rural Tanzania:the Kilombero District Study – a case series. *BMC International Health and Human Rights.* 2002; 30;2(1):4.

Gallup JL, Sachs JD. The Economic Burden of Malaria. *Am J Trop Med Hyg.* 2001; 64: 85-96.

GSK data on file: Investigator Brochure GlaxoSmithKline Biologicals' Candidate Vaccine Against *Plasmodium falciparum* Malaria (RTS,S/AS02A Malaria Vaccine). March 21 2003. Data on File at GlaxoSmithKline Biologicals.

GSK data on file: Phase IIb controlled randomized double-blind study to evaluate the efficacy, safety and immunogenicity of SmithKline Beecham Biologicals' candidate RTS,S malaria vaccine in semi-immune adult males in The Gambia. Protocol 257049/005 (Malaria-005) 12 December 1997. Data on File at GlaxoSmithKline Biologicals.

GSK data on file: A Phase I Double Blind, Randomized, Controlled, Staggered Study to Evaluate the Safety, Reactogenicity and Immunogenicity of 1/5, 1/2 and Full Doses of GlaxoSmithKline Biologicals' RTS,S/AS02 Candidate Malaria Vaccine, Administered IM According to a 0, 1, 3-month Vaccination Schedule in Semi-Immune Children aged 6 to 11 Years in The Gambia, a Malaria Endemic Region. Protocol 257049/015 (Malaria-015) April 17 2002. Data on File at GlaxoSmithKline Biologicals.

GSK data on file: A Phase I Double Blind, Randomized, Controlled, Staggered Study to Evaluate the Safety, Reactogenicity and Immunogenicity of 1/5, 1/2 and Full Doses of GlaxoSmithKline Biologicals' RTS,S/AS02 Candidate Malaria Vaccine, Administered IM According to a 0, 1, 3-month Vaccination Schedule in Semi-Immune Children aged 1

to 5 Years in The Gambia, a Malaria Endemic Region. Protocol 257049/020 (Malaria-020) April 18 2002. Data on File at GlaxoSmithKline Biologicals.

GSK data on file: A Phase I Double Blind, Randomized, Controlled, Staggered Study to Evaluate the Safety, Reactogenicity and Immunogenicity of GlaxoSmithKline Biologicals' RTS,S/AS02 Candidate Malaria Vaccine, Administered IM According to a 0, 1, 2-month Vaccination Schedule in Children aged 1 through 4 years in Mozambique, a Malaria Endemic Region. Protocol 257049/025 (Malaria-025) December 14, 2001. Data on File at GlaxoSmithKline Biologicals.

GSK data on file: A double-blind randomised controlled phase IIb study to evaluate the safety, immunogenicity and efficacy of GlaxoSmithKline Biologicals' candidate malaria vaccine RTS,S/AS02A, administered IM according to a 0, 1 and 2 month vaccination schedule in toddlers and children aged 1 to 4 years in a malaria-endemic region of Mozambique. Protocol 257049/026 (Malaria-026) 17 January 2003. Data on File at GlaxoSmithKline Biologicals.

GSK data on file: A Phase I/II randomized double-blind bridging study to evaluate the safety and immunogenicity of GlaxoSmithKline Biologicals' candidate vaccines RTS,S/AS02D (0.5 mL dose) and RTS,S/AS02A (0.25 mL dose) administered IM according to a 0, 1, 2 month vaccination schedule in children aged 3 to 5 years living in a malaria-endemic region of Mozambique. Protocol 257049/034 (Malaria-034) 03 October 2003. Data on File at GlaxoSmithKline Biologicals.

Iriso R. 'Killer' canines: the morbidity and mortality of ebino in northern Uganda *Tropical Medicine and International Health*. 2000; 5 (10); 706–710.

Kester KE, McKinney DA, Tornieporth N, Ockenhouse CF, Heppner DG, et al. Efficacy of recombinant circumsporozoite protein vaccine regimens against experimental *Plasmodium falciparum* malaria. *Journal of Infectious Diseases*. 2001; 15:183(4):640-7.

~~Rowe J, Macaubas C, Monger TM, Holt BJ, Harvey J, Poolman JT, et al. Antigen-specific responses to diphtheria-tetanus-acellular pertussis vaccine in human infants are initially Th2 polarized. *Infect Immun* 2000;68(7):3873-7.~~ ***(Amended 12 April 2005)***

Ryder RW, Oxtoby MJ, Mvula M, Batter V, Baende E et al. Safety and immunogenicity of bacille Calmette-Guerin, diphterhia-tetanus-pertussis, and oral polio vaccines in newborn children in Zaire infected with human immunodeficiency virus type 1. Journal of Pediatrics 1993 122; 5(1): 697-702.

Schellenberg D, Menendez C, Kahigwa E, Aponte J, Vidal J et al. Intermittent treatment for malaria and anaemia control at time of routine vaccinations in Tanzanian infants: a randomised, placebo-controlled trial. *The Lancet*. 2001; 357: 1471-1477.

Smith PG, Morrow RH. *Methods for Field Trials of Interventions against Tropical Diseases: A Toolbox.* Oxford University Press 1991: 152-155; 168-175.

Snow, RW, Schellenberg, JR, Peshu, D, Forster, CR, Newton, PA et al. Periodicity and space-time clustring of severe childhood malaria on the coast of Kenya. *Trans. R. Soc. Trop. Med. Hyg.* 1990: 87 (4): 386-390.

Snow, RW, de Azevedo Bastos, I, Lowe, EW, Kabiru, CG, Nevill, CG et al. Severe childhood malaria in two areas of markedly different falciparum transmission in east Africa. *Acta Trop.* 1994: 57 (4): 289-300, 1994.

Stoute JA, Slaoui M, Heppner DG, Momin P, Kester KE, Desmons P et al. A Preliminary Evaluation of a Recombinant Circumsporozoite Protein Vaccine against *Plasmodium falciparum* Malaria. N Eng J Med 1997; 336: 86-91

Vryheid RE, Kane MA, Muller N, Schatz GC, Bezabeh S. Infant and Adolescent Hepatitis B Immunization up to 1999: A Global Overview.*Vaccine* 2001;19: 1026-1037.

World Health Organization. Hepatitis B Fact Sheet WHO/204. www.who.int/inf-fs/en/fact204.html Accessed 11 April 2003.

Appendix A World Medical Association Declaration of Helsinki

**Recommendations guiding physicians
in biomedical research involving human subjects**

Adopted by the 18th World Medical Assembly
Helsinki, Finland, June 1964
and amended by the
29th World Medical Assembly
Tokyo, Japan, October 1975
35th World Medical Assembly
Venice, Italy, October 1983
41st World Medical Assembly
Hong Kong, September 1989
and the
48th General Assembly
Somerset West, Republic of South Africa, October 1996

INTRODUCTION

It is the mission of the physician to safeguard the health of the people. His or her knowledge and conscience are dedicated to the fulfilment of this mission.

The Declaration of Geneva of the World Medical Association binds the physician with the words, "The health of my patient will be my first consideration," and the International Code of Medical Ethics declares that, "A physician shall act only in the patient's interest when providing medical care which might have the effect of weakening the physical and mental condition of the patient."

The purpose of biomedical research involving human subjects must be to improve diagnostic, therapeutic and prophylactic procedures and the understanding of the etiology and pathogenesis of disease.

In current medical practice most diagnostic, therapeutic or prophylactic procedures involve hazards. This applies especially to biomedical research.

Medical progress is based on research which ultimately must rest in part on experimentation involving human subjects.

In the field of biomedical research a fundamental distinction must be recognized between medical research in which the aim is essentially diagnostic or therapeutic for a patient, and medical research, the essential object of which is purely scientific and without implying direct diagnostic or therapeutic value to the person subjected to the research.

Special caution must be exercised in the conduct of research which may affect the environment, and the welfare of animals used for research must be respected.

Because it is essential that the results of laboratory experiments be applied to human beings to further scientific knowledge and to help suffering humanity, the World Medical

Association has prepared the following recommendations as a guide to every physician in biomedical research involving human subjects. They should be kept under review in the future. It must be stressed that the standards as drafted are only a guide to physicians all over the world. Physicians are not relieved from criminal, civil and ethical responsibilities under the laws of their own countries.

I. BASIC PRINCIPLES

1. Biomedical research involving human subjects must conform to generally accepted scientific principles and should be based on adequately performed laboratory and animal experimentation and on a thorough knowledge of the scientific literature.
2. The design and performance of each experimental procedure involving human subjects should be clearly formulated in an experimental protocol which should be transmitted for consideration, comment and guidance to a specially appointed committee independent of the investigator and the sponsor provided that this independent committee is in conformity with the laws and regulations of the country in which the research experiment is performed.
3. Biomedical research involving human subjects should be conducted only by scientifically qualified persons and under the supervision of a clinically competent medical person. The responsibility for the human subject must always rest with a medically qualified person and never rest on the subject of research, even though the subject has given his or her consent.
4. Biomedical research involving human subjects cannot legitimately be carried out unless the importance of the objective is in proportion to the inherent risk to the subject.
5. Every biomedical research project involving human subjects should be preceded by careful assessment of predictable risks in comparison with foreseeable benefits to the subject or to others. Concern for the interests of the subject must always prevail over the interests of science and society.
6. The right of the research subject to safeguard his or her integrity must always be respected. Every precaution should be taken to respect the privacy of the subject and to minimize the impact of the study on the subject's physical and mental integrity and on the personality of the subject.
7. Physicians should abstain from engaging in research projects involving human subjects unless they are satisfied that the hazards involved are believed to be predictable. Physicians should cease any investigation if the hazards are found to outweigh the potential benefits.
8. In publication of the results of his or her research, the physician is obliged to preserve the accuracy of the results. Reports of experimentation not in accordance with the principles laid down in this Declaration should not be accepted for publication.
9. In any research on human beings, each potential subject must be adequately informed of the aims, methods, anticipated benefits and potential hazards of the study and the discomfort it may entail. He or she should be informed that he or she is

at liberty to abstain from participation in the study and that he or she is free to withdraw his or her consent to participation at any time. The physician should then obtain the subject's freely-given informed consent, preferably in writing.

10. When obtaining informed consent for the research project the physician should be particularly cautious if the subject is in a dependent relationship to him or her or may consent under duress. In that case the informed consent should be obtained by a physician who is not engaged in the investigation and who is completely independent of this official relationship.
11. In case of legal incompetence, informed consent should be obtained from the legal guardian in accordance with national legislation. Where physical or mental incapacity makes it impossible to obtain informed consent, or when the subject is a minor, permission from the responsible relative replaces that of the subject in accordance with national legislation.
    Whenever the minor child is in fact able to give a consent, the minor's consent must be obtained in addition to the consent of the minor's legal guardian.
12. The research protocol should always contain a statement of the ethical considerations involved and should indicate that the principles enunciated in the present Declaration are complied with.

II. MEDICAL RESEARCH COMBINED WITH PROFESSIONAL CARE

(Clinical research)

1. In the treatment of the sick person, the physician must be free to use a new diagnostic and therapeutic measure, if in his or her judgement it offers hope of saving life, re-establishing health or alleviating suffering.
2. The potential benefits, hazards and discomfort of a new method should be weighed against the advantages of the best current diagnostic and therapeutic methods.
3. In any medical study, every patient—including those of a control group, if any—should be assured of the best proven diagnostic and therapeutic method. This does not exclude the use of inert placebo in studies where no proven diagnostic or therapeutic method exists.
4. The refusal of the patient to participate in a study must never interfere with the physician–patient relationship.
5. If the physician considers it essential not to obtain informed consent, the specific reasons for this proposal should be stated in the experimental protocol for transmission to the independent committee (I, 2).
6. The Physician can combine medical research with professional care, the objective being the acquisition of new medical knowledge, only to the extent that medical research is justified by its potential diagnostic or therapeutic value for the patient.

III. NON-THERAPEUTIC BIOMEDICAL RESEARCH INVOLVING HUMAN SUBJECTS

(Non-clinical biomedical research)

1. In the purely scientific application of medical research carried out on a human being, it is the duty of the physician to remain the protector of the life and health of that person on whom biomedical research is being carried out.
2. The subjects should be volunteers—either healthy persons or patients for whom the experimental design is not related to the patient's illness.
3. The investigator or the investigating team should discontinue the research if in his/her or their judgement it may, if continued, be harmful to the individual.

In research on man, the interest of science and society should never take precedence over considerations related to the well being of the subject.

Appendix B Administrative Matters

I. Responsibilities of the Investigator

- To ensure that he/she has sufficient time to conduct and complete the study and has adequate staff and appropriate facilities and equipment which are available for the duration of the study and to ensure that other studies do not divert essential subjects or facilities away from the study at hand.
- To submit an up-to-date curriculum vitae and other credentials (e.g., medical license number in the United States) to GSK Biologicals and—where required—to relevant authorities.
- To acquire the normal ranges for laboratory tests performed locally and, if required by local regulations, obtain the Laboratory License or Certification.
- To ensure that no clinical samples (including serum samples) are retained on site or elsewhere without the approval of GSK Biologicals and the express written informed consent of the subject and/or the subject’s legally authorized representative.
- To perform no other biological assays at the investigator site except those described in the protocol or its amendment(s).
- To prepare and maintain adequate case histories designed to record observations and other data pertinent to the study.
- To conduct the study in compliance with the protocol and appendices.
- To co-operate with a representative of GSK Biologicals in the monitoring process of the study and in resolution of queries about the data.

II. Protocol Amendments and Administrative changes

- No changes to the study protocol will be allowed unless discussed in detail with the GSK Biologicals' Clinical Development Manager/Medical Monitor and filed as an amendment/administrative change to this protocol.
- Any amendment/administrative change to the protocol will be adhered to by the participating center(s) and will apply to all subjects. Written IRB/IEC approval of protocol amendments is required prior to implementation; administrative changes are submitted to IRBs/IECs for information only.
- Any amendment/administrative change to the protocol will be adhered to by the participating center(s) and will apply to all subjects. Written IRB/IEC approval of protocol amendments/administrative changes is required prior to implementation.

III. Sponsor’s Termination of Study

GSK Biologicals reserves the right to temporarily suspend or prematurely discontinue this study either at a single site or at all sites at any time for reasons including, but not limited to, safety or ethical issues or severe non-compliance. Reasons for suspension or early termination will be documented in the study file at GSK Biologicals.

If GSK Biologicals determines that suspension or early termination is needed, GSK Biologicals will discuss this with the Investigator (including the reasons for taking such action). When feasible, GSK Biologicals will provide advance notification to the investigator of the impending action prior to it taking effect.

GSK Biologicals will promptly inform, via written communication, all investigators and/or institutions conducting the study, if the study is suspended or terminated for safety reasons, and will also inform the regulatory authorities of the suspension or termination of the study and the reason(s) for the action. If required by applicable regulations, the investigator must inform the IEC/IRB promptly and provide the reason for the suspension or termination.

If the study is prematurely discontinued, all study data must be returned to GSK. In addition, arrangements will be made for all unused investigational product(s) in accordance with the applicable GSK procedures for the study. Financial compensation to investigators and/or institutions will be in accordance with the agreement established between the investigator and/or institutions and GSK.

IV. Case Report Form Instructions

Prior to screening the first potential participant, the investigator will provide the Site Monitor with a list (Site Staff Signature Sheet) showing the signature and hand-written initials of all individuals authorized to make or change entries on CRFs (already defined). If the authorized individuals should change during the study, the investigator is to inform GSK Biologicals of the specific change(s).

CRFs (and subject diary cards, if applicable), will be supplied by GSK Biologicals for recording all data. It is the responsibility of the investigator or co-investigator to ensure that CRFs (and subject diary cards) are legible and completely filled in with a black ink fountain or ballpoint pen.

Errors must be corrected by drawing a single line through the incorrect entry and writing in the new value/data positioned as close to the original as possible. The correction must then be initialed, dated and justified, where necessary, by the authorized individual making the change. The original entry must not be obliterated, overwritten or erased when a correction is made.

When a subject completes a visit, it is anticipated that relevant sections of the CRF will be completed by the investigator (or designated staff as documented in the Site Staff Signature Sheet) as soon as possible after the last data becoming available. Similarly, when a subject completes a study, it is anticipated that all relevant CRF pages will be completed promptly after the last data becoming available. This also applies to forms for potential study participants who were screened but not randomized to a study group.

As soon as the subject has completed/withdrawn from the study and the CRF is completed, the principal investigator or designated physician(s) under his/her supervision will sign the study conclusion pages of the CRF to confirm that they have reviewed the

data and that the data are complete and accurate. In all cases the investigator remains accountable for the study data collected.

An original (top copy) CRF or log sheets must be submitted for all subjects who have undergone protocol specific procedures, whether or not the subject completed the study.

While completed CRFs are reviewed by a GSK Biologicals professional monitor at the study site, errors detected by subsequent in-house CRF review may necessitate clarification or correction of errors with documentation and approval by the investigator or appropriately qualified staff as documented on the Site Staff Signature Sheet. In all cases, the investigator remains accountable for the study data. Wherever possible the investigator should assist in the clarification or correction of errors detected after study finalization promptly after being brought to the attention of the investigator (preferably within 48 hours).

Any questions or comments related to the CRF should be directed to the assigned Site Monitor.

V. Monitoring by GSK Biologicals

Monitoring visits by a professional representative of the sponsor will be scheduled to take place as close as possible to entry of the first subject, during the study at appropriate intervals and after the last subject has completed the study. It is anticipated that monitoring visits will occur at a frequency defined before study start.

These visits are for the purpose of confirming that GSK Biologicals' sponsored studies are being conducted in compliance with the relevant Good Clinical Practice regulations/ guidelines, verifying adherence to the protocol and the completeness and accuracy of data entered on the CRF pages and Vaccine Inventory Forms. The monitor will verify CRF entries by comparing them with the source data/documents that will be made available by the investigator for this purpose. Data to be recorded directly into the CRF pages screens will be specified in writing preferably in the source documentation agreement form that is contained in both the monitor's and investigator's study file. The investigator must ensure provision of reasonable time, space and adequate qualified personnel for monitoring visits.

VI. Archiving of Data

Following closure of the study, the investigator must maintain all site study records in a safe and secure location. The records must be maintained to allow easy and timely retrieval, when needed (e.g., audit or inspection), and, whenever feasible, to allow any subsequent review of data in conjunction with assessment of the facility, supporting systems, and staff. Where permitted by applicable laws/regulations or institutional policy, some or all of these records can be maintained in a validated format other than hard copy (e.g., microfiche, scanned, electronic for studies with an eCRF, for example); however, caution needs to be exercised before such action is taken. The investigator must assure that all reproductions are legible and are a true and accurate copy of the original, and meet accessibility and retrieval standards, including re-generating a hard copy, if

required. Furthermore, the investigator must ensure there is an acceptable back-up of these reproductions and that an acceptable quality control process exists for making these reproductions.

GSK will inform the investigator/ institution of the time period for retaining these records to comply with all applicable regulatory requirements. However, the investigator/ institution should seek the written approval of the sponsor before proceeding with the disposal of these records. The minimum retention time will meet the strictest standard applicable to that site for the study, as dictated by ICH GCP E6 Section 4.9, any institutional requirements or applicable laws or regulations, or GSK standards/procedures; otherwise, the minimum retention period will default to 15 years.

The investigator/ institution must notify GSK of any changes in the archival arrangements, including, but not limited to, the following: archival at an off-site facility, transfer of ownership of the records in the event the investigator leaves the site.

VII. Audits

For the purpose of compliance with Good Clinical Practice and Regulatory Agency Guidelines it may be necessary for GSK Biologicals or a Drug Regulatory Agency to conduct a site audit. This may occur at any time from start to after conclusion of the study.

When an investigator signs the protocol, he agrees to permit drug regulatory agencies and GSK Biologicals audits, providing direct access to source data/ documents. Furthermore, if an investigator refuses an inspection, his data will not be accepted in support of a New Drug Registration and/or Application, Biologics Licensing Application.

GSK Biologicals has a substantial investment in clinical studies. Having the highest quality data and studies are essential aspects of vaccine development. GSK Biologicals has a Regulatory Compliance staff who audit investigational sites. Regulatory Compliance assesses the quality of data with regard to accuracy, adequacy and consistency. In addition, Regulatory Compliance assures that GSK Biologicals sponsored studies are in accordance with GCP and that relevant regulations/guidelines are being followed.

To accomplish these functions, Regulatory Compliance selects investigational sites to audit. These audits usually take 1 to 2 days. The GSK Biologicals' audits entail review of source documents supporting the adequacy and accuracy of CRFs, review of documentation required to be maintained, and checks on vaccine accountability. The GSK Biologicals' audit therefore helps prepare an investigator for a possible regulatory agency inspection as well as assuring GSK Biologicals of the validity of the database across investigational sites.

The Inspector will be especially interested in the following items:

- Log of visits from the sponsor's representatives
- IRB/IEC approval

- Vaccine accountability
- Approved study protocol and amendments
- Informed consent of the subjects (written consent [or witnessed oral if applicable])
- Medical records and other source documents supportive of CRF data
- Reports to the IRB/IEC and the sponsor
- Record retention

GSK Biologicals will gladly help investigators prepare for an inspection.

VIII. Ownership, Confidentiality and Publication

Ownership:

All information provided by GSK and all data and information generated by the site as part of the study (other than a subject's medical records) are the sole property of GSK.

All rights, title, and interests in any inventions, know-how or other intellectual or industrial property rights which are conceived or reduced to practice by site staff during the course of or as a result of the study are the sole property of GSK, and are hereby assigned to GSK.

If a written contract for the conduct of the study which includes ownership provisions inconsistent with this statement is executed between GSK and the study site, that contract's ownership provisions shall apply rather than this statement.

Confidentiality:

All information provided by GSK and all data and information generated by the site as part of the study (other than a subject's medical records) will be kept confidential by the investigator and other site staff. This information and data will not be used by the investigator or other site personnel for any purpose other than conducting the study. These restrictions do not apply to: (1) information which becomes publicly available through no fault of the investigator or site staff; (2) information which it is necessary to disclose in confidence to an IEC or IRB solely for the evaluation of the study; (3) information which it is necessary to disclose in order to provide appropriate medical care to a study subject; or (4) study results which may be published as described in the next paragraph. If a written contract for the conduct of the study which includes confidentiality provisions inconsistent with this statement is executed, that contract's confidentiality provisions shall apply rather than this statement.

Publication:

For multicenter studies, the first publication or disclosure of study results shall be a complete, joint multicenter publication or disclosure coordinated by GSK. Thereafter, any secondary publications will reference the original publication(s).

Prior to submitting for publication, presentation, use for instructional purposes, or otherwise disclosing the study results generated by the site (collectively, a "Publication"), the investigator shall provide GSK and MVI at PATH with a copy of the proposed Publication and allow GSK and MVI at PATH a period of at least thirty (30) days [or, for abstracts, at least five (5) working days] to review the proposed Publication. Proposed Publications shall not include either GSK confidential information other than the study results or personal data on any subject, such as name or initials.

At GSK's request, the submission or other disclosure of a proposed Publication will be delayed a sufficient time to allow GSK to seek patent or similar protection of any inventions, know-how or other intellectual or industrial property rights disclosed in the proposed Publication.

If a written contract for the conduct of the study, which includes publication provisions inconsistent with this statement is executed, that contract's publication provisions shall apply rather than this statement.

Any publication or presentation shall state the following in an appropriate location: 'Funded in part by PATH'sMalaria Vaccine Initiative.

Appendix C Overview of the Recruitment Plan

Prior to enrollment a specific information policy towards the targeted population will be implemented. This will consist of a step by step approach, starting with the administrative and senior leaders of the community and the Organization of Mozambican Women (OMW) as well as health personnel from the Manhiça District and the parents of children in the community. The sessions will explain the problem of malaria to this community, the current strategies for its control, as well as the limitations of these strategies. The need and the difficulties of developing a vaccine against malaria will be discussed, as well as an outline of the proposed trials, including the rationale, the background data available and the study objectives. Particular attention will be paid to study procedures including screening of mothers for hepatitis B and HIV, immunization and blood collection. In that respect a full discussion on the purpose of blood collection and the associated risks will be carried out.

Study personnel will attend antenatal clinics in the study area. Women in the third trimester of pregnancy presenting to the antenatal clinics will be asked to consider enrolling their child in the vaccine trial. The study personnel will explain the trial to the women and give them a copy of the SIS for them to take home and review.

As a trial procedure all women who are considering enrolling their infant in the study will be screened for HBsAg and HIV. Written informed consent will be taken before any blood is taken for testing. Further details of the testing procedures are provided in the Study Protocol in Section 5.1.10.2. Women who test positive for either HBsAg or HIV will not be eligible to enroll their children in the trial.

The management of infants born to HBsAg positive women and HIV positive women and their children is provided in the Study Protocol in Section 5.1.10.2.1 and 5.1.10.2.2.

The site will compile a list of children whose mothers have expressed an interest in enrolling them in the study and who have been tested for HBsAg and HIV and found to be negative. When these infants are presented at an EPI clinic in Ilha Josina ***Health Center or Taninga Health Center,*** study personnel will seek individual informed consent for each child from the parent(s)/guardian(s) in privacy. Parent(s)/guardian(s) will again be informed about the study objectives and procedures including immunization and blood collection and they will be encouraged to ask and clarify questions about the trial. The parent(s)/guardian(s) understanding of the Informed Consent form will be verified by use of an oral assessment questionnaire (outlined in CISM SOPs). The parent(s)/guardian(s) and the witness will sign the Informed Consent form. Infants whose parent(s)/guardian(s) consent for them to enter the will be screened according to the procedures outlined in Section 5.1.10.3 of the Study Protocol ***(Amended 29 June 2005)***.

Appendix D Handling of Biological Samples Collected by the Investigator

Instructions for Handling of Serum Samples

When materials are provided by GSK Biologicals, it is MANDATORY that all clinical samples (including serum samples) will be collected and stored using exclusively those materials in the appropriate manner. The use of other materials could result in the exclusion of the subject from the ATP analysis. The investigator must ensure that his/her personnel and the laboratory(ies) under his/her supervision comply with this requirement. However, when GSK Biologicals does not provide material for collecting and storing clinical samples, then appropriate materials from the investigator's site are to be used.

1. Collection

The whole blood ~~(by capillary route)~~ should be collected observing appropriate aseptic conditions. It is recommended that Vacutainer® tubes WITH integrated serum separator (e.g. Becton-Dickinson Vacutainer® SST or Corvac® Sherwood Medical) be used to minimize the risk of hemolysis and to avoid blood cell contamination of the serum when transferring to standard serum tubes ***(Amended 12 April 2005)***.

2. Serum separation

These guidelines aim to ensure high quality serum by minimizing the risk of hemolysis, blood cell contamination of the serum or serum adverse cell toxicity at testing.

- For separation of serum using Vacutainer® tubes, the instructions provided by the manufacturer should be followed. Siliconized tubes should never be used (cell toxicity). Often the manufacturer's instruction states that the relative centrifugal acceleration known also as "G" must be "between 1000 and 1300 G" with tubes spinning for ten minutes. Error in calculation of centrifuge speed can occur when laboratory personnel confuse "G" acceleration with "RPM" (revolutions per minute). The speed of centrifugation must be calculated using the "G" rate provided in the manufacturer's instructions and the radius of the centrifuge head. After measuring the radius of the centrifuge machine, a speed/acceleration nomograph must be employed to determine the centrifuge speed in "RPM".
- Following separation, the serum should be aseptically transferred to the appropriate standard tubes using a sterile disposable pipette. The serum should be transferred as gently as possible to avoid blood cell contamination.
- The tube should not be overfilled (max. 3/4 of the total volume) to allow room for expansion upon freezing.
- The tube should be identified by the appropriate label provided by GSK Biologicals (see point 3).

3. Labeling

- The standard labels provided by GSK Biologicals should be used to label each serum sample.

- If necessary, any hand-written additions to the labels should be made using indelible ink.
- The label should be attached to the tube as follows (see diagram):
  - first attach the paper part of the label to the tube
  - then wrap the label around the tube so that the transparent, plastic part of the label overlaps with the label text and bar code and shields them.

  This will ensure optimal label attachment.

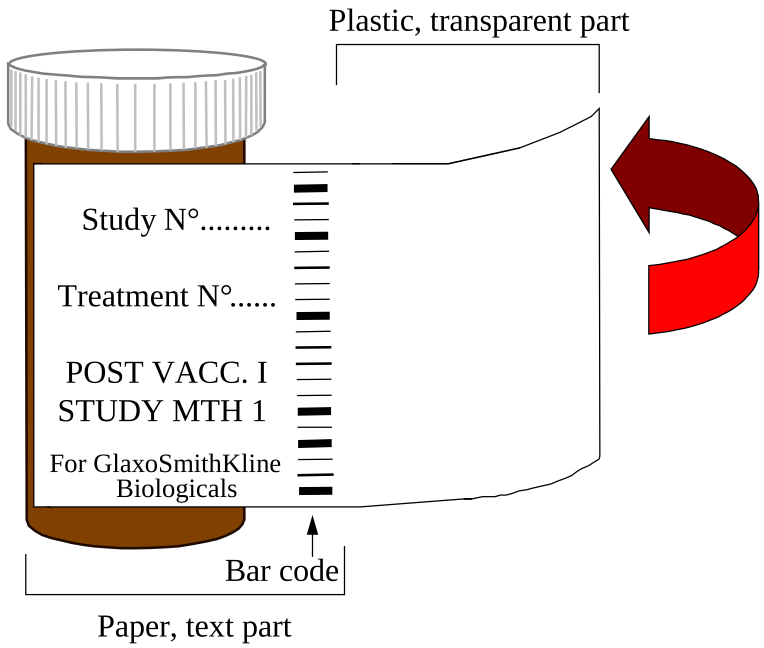

Labels should not be attached to caps.

4. Sorting and storage

- Tubes should be placed in the GSK Biologicals' cardboard boxes in numerical order from left to right, starting from the lower left hand corner, beginning with the pre-vaccination samples series, then with the post-vaccination sample series.
- The tubes of serum should be stored in a vertical position at approximately -20°C (alternatively at approximately -70°/80°C is also acceptable) until shipment to GSK Biologicals. The storage temperature should be checked regularly and documented. Wherever possible, a backup facility for storage of serum samples should be available.
- A standard Serum Listing Form, specifying the samples being shipped for individual subjects at each time point, should be prepared for each shipment. A copy of this list should be retained at the study site, while the original should be sealed in a plastic envelope and shipped with the serum samples.

- Once flight details are known, a standard Specimen Transfer Form must be completed and faxed to GSK Biologicals to the number provided below. A copy of the Specimen Transfer Form must be in the parcel[1]

GLAXOSMITHKLINE BIOLIGICALS
Attention Biospecimen Reception
Clinical Immunology
R & D Department/Building 44
Rue de l'Institut, 89
B-1330 Rixensart – Belgium

Telephone	+32-2-656 8949 or +32-2-656 6130
	or +32-2-656 8549 or +32-2-656 6108
Fax	+32-2-656 6052
E-mail	rix.ugbiospecimen-reception@gskbio.com

[1] The Serum Listing Form and the Specimen Transfer Form are standard documents used in GSK Biologicals' clinical trials. These documents are provided by GSK Biologicals' Clinical Trials' monitor at study initiation.

Appendix E Shipment of Biological Samples

Instructions for Shipment of Serum or Plasma Samples

Serum/plasma samples should be sent to GSK Biologicals at regular intervals. The frequency of shipment of samples should be decided upon by the Site Monitor, Central Study Coordinator and the investigator prior to the study start.

Serum/plasma samples should always be sent by air, preferably on a Monday, Tuesday or Wednesday, unless otherwise requested by the sponsor.

Serum/plasma samples must be placed with dry ice (maximum -20°C) in a container complying with International Air Transport Association (IATA) requirements. The completed standard serum listing form should always accompany the shipment.

The container must be clearly identified with the labels provided by GSK Biologicals specifying the shipment address and the storage temperature (-20°C).

The airway bill should contain the instruction for storage of samples at maximum -20°C.

A ‘proforma’ invoice, stating a value for customs purposes only, should be prepared and attached to the container. This document should contain the instruction for storage of samples at maximum -20°C.

Details of the shipment, including:

* number of samples
* airway bill
* flight number
* flight departure and arrival times

should be sent by fax or email two days before shipment, to:

GLAXOSMITHKLINE BIOLOGICALS,
Attention Biospecimen Reception
Clinical Immunology
R & D Department/Building 44
Rue de l’Institut, 89
B-1330 Rixensart – Belgium

Telephone +32-2-656 8949 or +32-2-656 6130
or +32-2-656 8549 or +32-2-656 6108
Fax +32-2-656 6052
E-mail rix.ugbiospecimen-reception@gskbio.com

The central study coordinator, Sabine Corachan and the local monitor, ~~Andreas Pakendorf,~~ ***Adelheid Davis*** should be informed 2 days before any shipment

Sabine Corachan, Rixensart, Belgium	~~Andreas Pakendorf &~~ Adelheid Davis, Bryanston, South Africa
Telephone: +32.2.656.92.33	Telephone: +27.11.745.63.24
Fax: +32.2.656.61.60	Fax: +27.11.745.74.95
email: sabine.corachan@gskbio.com	email: ~~andreas.m.pakendorf@gsk.com &~~ adelheid.a.davis@gsk.com

(Amended 29 June 2005).

Appendix F Laboratory Assays

Serology testing

Serological responses will be measured principally by evaluating antibody responses to HBs and to CSP repeats (anti R32LR). Serum for antibody determination will be collected at the time points defined in the flowchart in Sections 5.4.

Antibody levels against CS will be measured at GSK Biologicals (or a designated laboratory) by standard ELISA methodology using plate adsorbed R32LR antigen with a standard reference antibody as a control according to SOPs from the laboratory. Results will be reported in EU/mL.

Antibody to hepatitis B surface antigen will be measured at GSK Biologicals using a commercially available ELISA immunoassay (AUSAB EIA test kit from Abbott) or equivalent according to the assay instructions. Results will be reported in mIU/mL.

Total antibodies against the Hib polysaccharide PRP will be measured by ELISA using an in-house assay of GSK Biologicals. The cut-off is 0.15 µg/mL.

Specific antibodies against diphtheria and tetanus toxoid will be measured by ELISA techniques by GSK Biologicals. The cut-off of the tests is 0.1 IU/mL.

Anti-BPT antibody titers will be determined by ELISA using the IgG EIA test kit Lab Systems by GSK Biologicals, and expressed in EL.U/mL with an assay cut-off of 15 EL.U/mL.

Determination of parasitemia

Estimates of asexual *P. falciparum* parasite density will be made at CISM according to laboratory SOPs. Two slides will be air dried, stained with Giemsa and read on a light microscope with a x50 oil immersion lens and x10 eyepieces. Parasite density will be assessed by counting the number of asexual stage parasites per 200 leukocytes. Slides will be declared negative only after 200 leukocytes have been read. Parasite numbers will be converted to a count/µl by assuming a standard leukocyte count of 8000/µL. All slides will be read twice independently, and a third time if the ratio of densities from the first two is greater than 1.5 or smaller than 0.67 or if there is a discrepancy in positivity. If less than 30 parasites are counted a third reading by a different reader will be done if the difference in the number of parasites is greater than 10. The definitive result will be based on the majority verdict for positivity and the geometric mean of the positive densities for positive slides.

Determination of parasite genotyping by polymerase chain reaction

Each time a malaria smear is obtained during the surveillance period for primary and secondary endpoints, whether for routine collections or to evaluate clinical symptoms, a few drops of blood will be collected onto filter paper for PCR analysis according to the SOP for this procedure. This collection will be noted on the CRF appropriate to the situation. Samples will be collected, stored individually in plastic bags according to SOP,

and transferred to a separate laboratory where PCR analysis will be performed. DNA will be extracted subsequently using standard methods. To determine the effect of RTS,S on parasite subpopulations, the distribution of variable alleles of vaccine and non-vaccine type in the CSP gene will be studied in parasites obtained from subjects vaccinated with the RTS,S vaccine or control vaccine using PCR and specific oligonucleotide or sequencing techniques.

CMI testing

Cell-mediated immune responses specific to CS will be assessed by Intracellular Cytokine Staining. This technology allows the quantification of T-cells specific to a given antigen.

Briefly, whole blood taken from the subject at various time points, is stimulated by pools of peptides representing the aminoacid sequence of the CS antigen. The antigen-specific CD4+ and CD8+ T cells producing IFN-gamma ***IL-2 and TNF alpha*** are enumerated by flow cytometry following conventional imunofluorescence oncomit of cellular phenotype and intracellular cytokines production. The data are expressed as frequency of CD4+ or CD8+ T cells expressing the cytokine ***(Amended 29 June 2005)***.

Biochemical and hematological analyses

Hematological and biochemical testing will be done at CISM in Mozambique, following laboratory SOPs.

~~**Analysis of determinants of vaccine response**~~

~~Genomic DNA will be extracted from whole blood specimens using standard methods and multiple displacement amplification (MDA) technology will be used to amplify whole genome DNA to ensure that there is an adequate amount for all genotyping. The genetic assessment will focus on variations in genes affecting immunological responses to infection with malaria as well as those affecting Th1 and Th2 responses. A combination of several polymorphisms in one gene or across different genes (i.e. a haplotype) may be required for a particular response to a vaccine. In genes for which such haplotypes have been described and related to phenotype, haplotype analysis will be done due to the higher informative value of these analyses compared with separate studies on single polymorphisms. For genes in which this is not the case, the data for potential haplotypes and their relationship with phenotype will be I. Genotyping will be done using high throughput methodologies including the Sequenom multiplexing, denaturing high performance liquid chromatography, Taqman® or restriction enzyme digestions, and all genotyping results will be routinely confirmed by sequencing in a subset of samples.~~

(Amended 12 April 2005)

Appendix G Vaccine supplies, packaging and accountability

It is NOT permitted to use any of the supplies provided by GSK Biologicals for purposes other than those specified in the protocol. Unused supplies will be collected by GSK Biologicals on completion of the study. Used vaccine vials/pre-filled syringes/containers can be disposed on site according to local biosafety standard for disposal of biological waste material.

1. Vaccine supplies

GSK Biologicals will supply the following amounts of numbered doses of study vaccines, sufficient to administer 3 dose(s) to all subjects as described in the present protocol.

- 330 doses of the candidate vaccine RTS,S/AS02D (330 doses of RTS,S vaccine in monodose vials and 330 doses of AS02D adjuvant in pre-filled syringes).
- 330 doses of the vaccine Engerix-B.
- 660 doses of the vaccine TETRActHib (660 doses of D.T.COQ/D.T.P. vaccine in monodose vials and 660 doses of Act-Hib *Hemophilus influenzae* type b polysaccharide conjugated with tetanus protein as a powder contained in a vial. One full monodose of D.T.COQ/D.T.P. should be mixed with the vial of Act-Hib to provide one dose of TETRActHib).

An additional 5% of their respective amounts of RTS,S/AS02D, Engerix-B and TETRActHib will be supplied for replacement in case of breakage, bad storage conditions or any other reason that would make the vaccine unusable (i.e., given by mistake to another subject).

All pre-filled syringes and vials must be accounted for on the form provided.

2. Vaccine packaging

The vaccines will be packed in labeled boxes. The box label will contain, as a minimum, the following information: study number, abbreviated title, treatment number, lot number (or numbers, when double-blind), instructions for vaccine administration.

3. Vaccine accountability

The investigator or pharmacist must sign a statement that he/she has received the clinical supplies for the study. At all times the figures on supplied, used and remaining vaccine doses should match. At the end of the study, it must be possible to reconcile delivery records with those of used and unused stocks. An explanation must be given of any discrepancies.

After approval from GSK Biologicals, used vaccine vials/syringes should be destroyed at the study site using locally approved biosafety procedures and documentation unless otherwise described in the protocol. If no adequate biosafety procedures are available at the study site, the used vaccine vials/syringes are to be returned to an appropriate GSK

Biologicals site for destruction in accordance with current GSK SOP WWD-1102. Unused vaccine vials/syringes will be disposed at the local GSK Biologicals site in accordance with GSK SOP WWD-1102. If no processes for destruction of unused vaccines are in place in the local GSK Biologicals site; the unused vials/syringes must be returned to GSK Biologicals in Rixensart, Belgium.

4. Transfers of clinical vaccines or products from country medical department or dispatch center to study sites or between sites

Storage temperatures must be maintained during transport and deviations must be reported to Logistics and Packaging for guidance. All transfers of clinical vaccines or products must be documented using the Clinical Supply Transfer Form. If the duration of the transfer is less than four hours, a transportable fridge or any suitable container (e.g. Styrofoam container) with a maximum of eight refrigerated cold packs (cooling elements) must be used in order to maintain the vaccines at 2°to 8°C during transport. If the duration is more than four hours, a transportable fridge or any suitable container (e.g. Styrofoam container) with a minimum of eight cold packs (cooling elements) must be used as well as a temperature monitoring system that must be placed as close as possible to the doses and checked upon reception at the final destination. Never place frozen cold packs or dry ice inside vaccine/product boxes for vaccine that must be kept at +4°C in order to avoid cold-chain deviation (e.g. frozen vaccines). Exceptions to these instructions are detailed in product-specific transport guidelines.

5. Labels for sample identification

The investigator will receive labels from GSK Biologicals to identify samples taken from each subject at each time point. Each label will contain the following information: study number, treatment number, sampling time point (e.g., post vaccination 3), timing (e.g., study Month 7).

6. Other supplies provided by GSK Biologicals

In addition to the vaccines, the study documentation and the sample labels, the investigator will receive the following supplies:

- tubes with screw caps for serum samples,
- racks for the tubes of serum.

Appendix H Amendments and Administrative Changes to the Protocol

**GlaxoSmithKline Biologicals**<br>Clinical Research & Development<br>**Protocol Amendment Approval**	
**eTrack study number**	103967
**eTrack abbreviated title**	Malaria-038
**IND number**	BB-IND 10514
**Protocol title:**	A Phase I/IIb randomized, double-blind, controlled study of the safety, immunogenicity and proof-of-concept of RTS,S/AS02D, a candidate malaria vaccine in infants living in a malaria-endemic region
**Amendment number:**	Amendment 1
**Amendment date:**	17 January 2005
**Co-ordinating author:**	Conor Cahill, Scientific Writer

Rationale/background for changes:
It has been decided that the exclusion criterion relating to weight of children at screening did not adequately reflect the average birthweights of the local community.

The minimum weight for children at screening in the trial is now expressed as a weight for age z-score (children should have a weight for age z-score of greater than -2 at screening). This corresponds to a weight of ≥ 3.9 kg for 2 month old boys and ≥ 3.6 kg for 2 month old girls. In order to help study personnel carrying out the screening visit, the suitable kg weights for 2 month old boys and girls are also cited in the exclusion criteria.

Amended text has been included in *bold italics* in the following section(s):
<u>**Section 4.4:**</u>
~~Weight ≤ 2.5 kg at screening~~. ***Moderate malnutrition defined as weight for age z-score less than -2; this corresponds to a weight of ≥ 3.9 kg for 2 month old boys and ≥ 3.6 kg for 2 month old girls.***
<u>**References:**</u>
Center for Disease Control. National Health and Nutrition Examination Survey. http://www.cdc.gov/nchs/about/major/nhanes/growthcharts/zscore/zscore.htm. Accessed 17 January 2005

GlaxoSmithKline Biologicals<br>Clinical Research & Development<br>**Protocol Amendment Approval**	
**eTrack study number**	103967
**eTrack abbreviated title**	Malaria-038
**IND number**	BB-IND 10514
**Protocol title:**	A Phase I/IIb randomized, double-blind, controlled study of the safety, immunogenicity and proof-of-concept of RTS,S/AS02D, a candidate malaria vaccine in infants living in a malaria-endemic region
**Amendment number:**	Amendment 2
**Amendment date:**	12 April 2005
**Co-ordinating author:**	Conor Cahill, Scientific Writer

Rationale/background for changes:

The CISM, GSK Biologicals and MVI teams have decided to remove assessment of Determinants of Vaccine Response from this study. All references to the assessment have been removed throughout the protocol.

In order to ensure that there is sufficient blood available to carry out Cell-Mediated Immunity assessment at protocol-defined timepoints it is necessary to raise the amount of blood to be collected at these timepoints from 1 mL to 2 mL. The sampling methodology is not specified per-protocol but is clarified in the informed consent documents.

A further elimination criterion has been added in order to ensure that children who fail to thrive will not receive experimental vaccine.

In order to assess the pertussis response it is necessary to account for maternally acquired immunity. Assessment of antibodies to pertussis at screening was omitted in previous versions of the protocol. This has been corrected in this amendment.

Text and a table have been added to help clarify the time periods between which different classes of oncomitant medications/treatments must be recorded.

A number of typographical errors that have been noticed since the previous amendment was finalized have been corrected in this version

Amended text has been included in *bold italics* in the following sections (Deletions are indicated with strikethrough, thus [~~capillary~~]):

Cover Sheet

Centro de Investigação em Saúde de Manhiça (CISM), Mozambique and Hospital Clinic (University of Barcelona), Spain
Contributing Authors

- ***Montse Renom, Project Manager***

Synopsis

- Two weeks prior to Dose 3 of ~~TETRActHib~~ ***RTS,S/AS02D***, children will be treated with sulfadoxine-pyrimethamine and Amodiaquine for presumptive clearance of parasitemia.

Section 1.5: Rationale for studying the determinants of vaccine response

~~Molecular techniques provide the means to address the problems of individual and group variations in specific vaccine responses, and to gain further insight into the general mechanisms of response to vaccines. These technologies have recently undergone substantial development and are now able to successfully dissect the mechanisms in human disease.~~

~~This study of the response to malaria vaccination will examine two types of genes. The first group would be candidate genes known to be important in inflammatory or immunological responses to infection with malaria and these should be investigated with respect to the potential effects of variations in these genes. Candidate genes specific for malaria vaccination responses would include genes that: (1) are known to be directly involved in infection with malaria; (2) have common polymorphisms that affect the amount or function of the protein produced by the gene; or (3) are related to a specific marker of infection with malaria. A list of potential candidate genes is provided below (Table 19). To date, there have been no reports examining the role of these or other polymorphisms on response or non-response to malaria vaccines. The second group of genes are those along the immunological pathways that are most likely to be important in protection from infection and the most obvious of these are the Th1/Th2 immune response pathways. Indeed, at the early time of life that vaccination is given, there is a general delay in establishment of Th1 responses and the initial response to vaccine antigens is initially strongly Th2 biased [Rowe, 2000]. A list of genes that are directly or indirectly involved in Th1/Th2 responses is provided below (Table 19).~~

Table 19 Candidate genes for determinants of vaccine response study

Candidate genes for malaria infection	Genes involved directly or indirectly with Th1/Th2 immunity
Intercellular adhesion molecule 1 (ICAM-1)	Gly241Arg, Glu469Lys
IL-10	1082G/A, 819C/T, 592C/A
CD36	14T/C, 53G/T, 1439G/C
CD14	159C/T, 1359G/T, 1145A/G
Inducible nitric oxide synthase (iNOS)	1173C/T
IL-4	1098T/G, 589C/T, 33C/T
Complement receptor 1 (CR1)	3650A/G
IL-4R alpha	Ile50Val, Gln551Arg, Ser478Pro
Mannose-binding protein (MBP)	Gly54Asp, Gly57Glu
IL-13	1111C/T, Arg130Gln, 4738G/A
Tumor necrosis factor alpha (TNF alpha)	308G/A
TLR4	2026A/T, 1607T/C, Asp299Gly, Thr399Ile
Toll-like Receptor (TLR2)	Arg677Trp, Arg753Gln
IL-1beta	511C/T, 3953C/T
IL-6	174G/C, 572G/C
IL-12B	1188A/C
IFN gamma receptor 1 (IFN gamma R1)	56T/C

Section 3: Study Design Overview

- Two weeks prior to Dose 3 of TETRActHib ***RTS,S/AS02D***, children will be treated with sulfadoxine-pyrimethamine and Amodiaquine for presumptive clearance of parasitemia.

Section 4.5: Elimination criteria during the study

- ***Failure to thrive***

Section 5.1.17: Active detection of infection

Surveillance will continue with Clinic Visit 10 on Day 104 (Month 3½) and then field worker visits every two weeks (one visit every 14 days) for the following 2½ months (six ***four*** visits).

If the child is well and afebrile, a capillary blood-sample will be taken and examined for malaria parasitemia and parasite genotyping.

There the child will be reviewed by a clinician or medical agent or assistant, who will complete the CISM clinic morbidity surveillance questionnaire and the child will be managed according to the procedures detailed above (see Section 5.1.14) and a capillary blood sample will be taken for determination of parasitemia and parasite genotyping.

Section 5.3: Outline of study procedures

Table 20 List of study procedures

	SCREEN	DOUBLE-BLIND PHASE																	SINGLE-BLIND PHASE	
		VACCINATION													ADI					
**Study Months**		**0**					**1**				**2**				**3**		**4-5**	**6**	**7-13**	**14**
**Study days**	-14 to 0	0		14		21	30		44		60		74		90	104		180		
**Age (approx. weeks)**	6 to 8	8		10		11	12		14		16		18		20	22		~~30~~ ***32***		70
**Clinic Visit**	1	2		3		4	5		6		7		8		9	10		11		12
**Field Worker Visit Code #**			*21-26*		*27-31*			*32-37*		*38-43*		*44-49*		*50-55*			*56-59*		*60-66*	
~~Determinants of vaccine response~~						~~•~~														
Antibodies to Pertussis	•														•					

Section 5.4: Detailed description of study stages/visits

Clinic Visit 1: Screening
Day -14 to Day 0

- ~~Capillary~~ Blood sample to collect a minimum ~~1~~ ***2*** mL blood for analysis of:
  - hematology (complete blood count)
  - biochemistry (oncomitan, ALT and bilirubin)
  - serology (antibodies to CS**,** ~~and~~ antibodies to HBs ***and pertussis***)
  - cell-mediated immunity

Clinic Visit 4: Collection of blood sample
Day 20

- ~~Capillary~~ Blood sample to collect a minimum 1 mL blood for analysis of
  - hematology (complete blood count)
  - biochemistry (oncomitan, ALT and bilirubin)
  - ~~determinants of vaccine response~~

Clinic Visit 8: Dose 3 of RTS,S/AS02D or Engerix-B
Check for clearance of malaria parasitemia
Day 74

- ~~Capillary~~ Blood sample to collect a minimum 1 mL blood for:

Clinic Visit 9: First Clinic Visit for ADI
Day 90

- ~~Capillary~~ Blood sample to collect a minimum 1 mL whole blood for analysis of:

Clinic Visit 10: Second Clinic Visit for ADI
Day 104, Month 3½

- ~~Capillary~~ Blood sample to collect a minimum ~~1~~ ***2*** mL whole blood for analysis of:

Monthly field-worker home visits (for ADI) 56 to 59
Month 4, 4½, 5, 5½

- ~~Capillary~~ Blood sample to collect a minimum 1 mL blood for:

Clinic Visit 11: Final Visit for ADI
Day 180, Month 6

- ~~Capillary~~ Blood sample to collect a minimum ~~1~~ ***2*** mL whole blood for analysis of:

Clinic Visit 12: Final Study Visit
Month 14

- ~~Capillary~~ Blood sample to collect a minimum 1 mL whole blood for analysis of:

<u>Section 5.5.2: Laboratory assays</u>

Laboratory tests at screening: Clinic Visit 1
Day -14 to Day 0

Collection of a minimum ~~1~~ ***2*** mL whole blood for baseline analysis of:

Laboratory tests during the study: Clinic Visit 4
Day 20

~~Capillary~~ Blood sample to collect a minimum 1 mL blood for analysis of:

- hematology (complete blood count)
- biochemistry (oncomitan, ALT and bilirubin)
- ~~determinants of vaccine response~~

Clinic Visit 8: Dose 3 of RTS,S/AS02D or Engerix-B
Check for clearance of malaria parasitemia
Day 74

~~Capillary~~ Blood sample to collect a minimum 1 mL blood for analysis of:

Clinic Visit 9: First Clinic Visit for ADI
Day 90

~~Capillary~~ Blood sample to collect a minimum 1 mL blood for analysis of:

Clinic Visit 10
Month 3½

~~Capillary~~ Blood sample to collect a minimum ~~1~~ ***2*** mL blood for analysis of:

Monthly field-worker home visits (for ADI) 56 to 59
Month 4, 4½, 5, 5½

~~Capillary~~ Blood sample to collect a minimum 1 mL blood for analysis of:

Clinic Visit 11: Final Visit for ADI
Month 6

~~Capillary~~ Blood sample to collect a minimum ~~1~~ ***2*** mL blood for analysis of:

Clinic Visit 12: Final Study Visit
Month 14

~~Capillary~~ Blood sample to collect a minimum 1 mL blood for analysis of:

Table 22 Summary of laboratory immunology tests to be performed

Assay	Marker	Assay method	Test Kit/ Manufacturer	Assay unit	Assay cut-off	Laboratory
Anti-CS antibodies	R32LR	ELISA	In-house ELISA	EU/mL	0.5	Leroux-Roels Laboratory, Ghent, Belgium
Anti-HBs antibodies		EIA	AUSAB EIA ABBOTT†	mIU/mL	10**	GSK Bio*, Rixensart
Anti-Pertussis antibodies		ELISA	IgG EIA (ICN-FLOW)	EL.U/mL	15	GSK Bio*, Rixensart
Anti-Diphtheria antibodies		ELISA	In-house ELISA	IU/mL	0.1	GSK Bio*, Rixensart
Anti-PRP antibodies		ELISA	In-house ELISA	µg/mL	0.15	GSK Bio*, Rixensart
Anti-Tetanus antibodies		ELISA	In-house ELISA	IU/mL	0.1	GSK Bio*, Rixensart
CS-specific CMI		ICS	In-house ICS	% cytokine positive cells		CISM or GSK Bio*
~~Determinants of vaccine response~~		~~high throughput genotyping~~	~~TBD~~	~~TBD~~		~~CISM*~~

* or designated validated laboratory
** seroprotective level
† or equivalent
CMI: cell-mediated immunity
ELISA: Enzyme-linked Immunoabsorbent Assay
EIA: Enzyme Immunoassay
ICS: intra-cellular cytokine staining

~~The determinants of vaccine response will be analyzed by high throughput genotyping including Sequenom multiplexing, denaturing high performance liquid chromatography, Taqman®, restriction enzyme digestions, and sequencing. This will be done at the CISM laboratory, the Center for International Health Laboratory, University of Barcelona, or a designated validated laboratory.~~

Section 5.5.3: Serology Plan

Serum for antibody determination will be collected by ~~capillary~~ blood sample (as specified at each visit in Sections 5.4 and 5.5). Samples for safety will be analyzed at the time they are collected

Table 23 Summary of blood sampling timepoints/*immunological assays*

Blood sampling timepoint			Test	No. subjects	Laboratory	Priority Rank
**Timing**	**Timepoint**	**Clinic Visit No.**				
Pre Dose 1	Screening	1	Anti-HBs antibodies	~~200~~ ***220***	GSK Bio*	***3*** ~~2~~
			Anti-CS antibodies	~~200~~ ***220***	GSK Bio*	***2*** ~~1~~
			Anti-Pertussis Antibodies	***200***	***GSK Bio****	***1***
			CMI	~~200~~ ***220***	CISM	***4*** ~~3~~
~~Post Dose 1~~	~~Month 0~~	4	~~Determinants of Vaccine Response~~	~~200~~	~~CISM*~~	~~1~~
Post Dose 3	Month 3	9	Anti-PRP antibodies	~~200~~ ***220***	GSK Bio*	1
			Anti-Pertussis antibodies	~~200~~ ***220***	GSK Bio*	2
			Anti-Diphtheria antibodies	~~200~~ ***220***	GSK Bio*	3
			Anti-Tetanus antibodies	~~200~~ ***220***	GSK Bio*	4
Post Dose 3	Month 3½	10	Anti-HBs antibodies	~~200~~ ***220***	GSK Bio*	2
			Anti-CS antibodies	~~200~~ ***220***	GSK Bio*	1
			CMI	~~200~~ ***220***	~~GSK Bio*~~ ***CISM***	3
Post Dose 3	Month 6	11	Anti-CS antibodies	~~200~~ ***220***	GSK Bio*	1
			CMI	~~200~~ ***220***	CISM	2
Post Dose 3	Month 14	12	Anti-HBs antibodies	~~200~~ ***220***	GSK Bio*	1
			Anti-CS antibodies	~~200~~ ***220***	GSK Bio*	2

*or designated laboratory
CMI: cell-mediated immunity
(Amended 12 April 2005)

Section 6.2.3 Engerix-B (0.5 mL dose)

The vaccine should be administered soon afterwards intramuscularly ~~in the deltoid region~~, using a 25G needle with length of 1 inch (25 mm) in the left antero-lateral thigh (storage at 2°C to 8°C)

Section 6.9: Concomitant medication/treatment

All antimalarial drugs administered during the period starting at 31 days post Dose 3 of RTS,S/AS02D and ending with the last visit for ADI are to be recorded with generic name of the medication (trade names are allowed for combination drugs, i.e., multi-component drugs), medical indication, total daily dose, route of administration, start and end dates of treatment.

Any treatments and/or medications which are listed as elimination criteria in Section 4.5, e.g., any immunoglobulins, other blood products and any immune modifying drugs administered within three months preceding the first dose or at any time during the study period are to be recorded with generic name of the medication (trade names are allowed for combination drugs only), medical indication, total daily dose, route of administration, start and end dates of treatment. Refer to Sections 4.4 and 4.5. ***The time periods between***

which each type of oncomitant medication/treatment should be recorded is summarized in Table 24.

Table 24 Summary of time periods between which different classes of oncomitant medication/treatment must be recorded

3 months prior to Dose 1 → Dose 1	*All treatments listed as elimination criteria in Section 4.5**
Dose 1 → 30 Days post Dose 3	*All antipyretic, analgesic, antibiotic, antimalarial and any treatments listed as elimination criteria in Section 4.5**
31 Days post Dose 3 → last visit for ADI	*All antimalarial drugs and treatments listed as elimination criteria in Section 4.5**
Last visit for ADI → Final Study Visit	*All treatments listed as elimination criteria in Section 4.5**

** e.g. any immunoglobulins, other blood products and any immune modifying drugs*

Section 10.9.3.2: Calculation of the time at risk

For the efficacy endpoint (time to first malaria infection), the time at risk will be counted in days, and expressed as child years at risk (days/365.25).

For each subject in the Total Cohort, time at risk begins 14 days after Dose 3 for those that received Dose 3 or 2½ months after Dose 1 (Day ~~75~~ ***90***) otherwise. For the According to Protocol cohort, time at risk begins 14 days after Dose 3.

References

~~Rowe J, Macaubas C, Monger TM, Holt BJ, Harvey J, Poolman JT, et al. Antigen-specific responses to diphtheria-tetanus-acellular pertussis vaccine in human infants are initially Th2 polarized. *Infect Immun* 2000;68(7):3873-7.~~

Appendix D: Handling of Biological Samples Collected by the Investigator

1. Collection

The whole blood ~~(by capillary route)~~ should be collected observing appropriate aseptic conditions. It is recommended that Vacutainer® tubes WITH integrated serum separator (e.g. Becton-Dickinson Vacutainer® SST or Corvac® Sherwood Medical) be used to minimize the risk of hemolysis and to avoid blood cell contamination of the serum when transferring to standard serum tubes.

Appendix F: Laboratory Assays

~~**Analysis of determinants of vaccine response**~~

~~Genomic DNA will be extracted from whole blood specimens using standard methods and multiple displacement amplification (MDA) technology will be used to amplify whole genome DNA to ensure that there is an adequate amount for all genotyping. The genetic assessment will focus on variations in genes affecting immunological responses to infection with malaria as well as those affecting Th1 and Th2 responses. A combination of several polymorphisms in one gene or across different genes (i.e. a haplotype) may be required for a particular response to a vaccine. In genes for which~~

~~such haplotypes have been described and related to phenotype, haplotype analysis will be done due to the higher informative value of these analyses compared with separate studies on single polymorphisms. For genes in which this is not the case, the data for potential haplotypes and their relationship with phenotype will be I. Genotyping will be done using high throughput methodologies including the Sequenom multiplexing, denaturing high performance liquid chromatography, Taqman® or restriction enzyme digestions, and all genotyping results will be routinely confirmed by sequencing in a subset of samples.~~

GlaxoSmithKline Biologicals

Clinical Research & Development

Protocol Amendment Approval

**eTrack study number**	103967
**eTrack abbreviated title**	Malaria-038
**IND number**	BB-IND 10514
**Protocol title:**	A Phase I/IIb randomized, double-blind, controlled study of the safety, immunogenicity and proof-of-concept of RTS,S/AS02D, a candidate malaria vaccine in infants living in a malaria-endemic region
**Amendment number:**	Amendment 3
**Amendment date:**	23 June 2005
**Co-ordinating author:**	Conor Cahill, Scientific Writer

Rationale/background for changes:

In order to expedite the recruitment for this trial, a second health center will be used to recruit mothers and vaccinate infants in addition to the health center at Ilha Josina. This second health center, Taninga, is similar to that at Ilha Josina. All facilites available at Ilha Josina are also available at Taninga and all staff are trained to the same standard. It is proposed to carry out all procedures in exactly the same manner as for those mothers and infants recruited at Ilha Josina. A detailed description of the facilities available at the Taninga Health Center have been added to the protocol with this amendment

At the request of the Food and Drug Administration, a rationale for the proposed interim analysis at 1 month post final dose of vaccine has been added.

An error in the safety monitoring plan has been corrected. The text describing the laboratory values that would be made available to the DSMB was not logical. The text has been corrected in order to ensure the original intent of providing the maximum possible amount of laboratory data to the DSMB.

An error in the ‘Process if the trial is suspended’ has been corrected. It has been clarified that the DSMB may suspend the trial temporarily. However should it be necessary to stop the trial permanently, the responsibility lies with the Sponsor, GSK.

Details of the proposed CMI testing have been clarified

One of the GSK local study monitors (Andreas Pakendorf) will not be taking part in the monitoring of this trial and has been removed from the document as a study contact.

One of the GSK authors was inadvertently left off the authorship list on the cover page. They have now been added. In addition, a number of new staff members at CISM/Hospital Clinic, Barcelona have been added to the list of authors
**Amended text has been included in *bold italics* in the following section(s):**
**Cover Pages:**<br><br>• ***Sabine Corachan, Central Study Coordinator***<br>• ***Arnoldo Barbosa Immunologist***<br>• ***Anna Berenguera***<br>• ***Denise Naniche, Immunologist***
**Local GSK Study Monitor**<br><br>~~Andreas Pakendorf &~~ Adelheid Davis,<br>GlaxoSmithKline,<br>Private Bag X173,<br>Bryanston 2021,<br>South Africa.<br><br>Tel: +27.11.745.63.24<br>Fax: +27.11.745.74.95<br>email: ~~andreas.m.pakendorf@gsk.com &~~ adelheid.a.davis@gsk.com
**Study Synopsis:**<br><br>• Recording of serious adverse events will be throughout the study period. They will be captured through the morbidity surveillance system at Ilha Jossina Health Center***, Taninga Health Center*** and Manhiça District Hospital. In addition all enrolled children will be visited at home monthly by field workers until study conclusion to ensure complete identification of all SAEs.
**Section 1.7:** ***Rationale for Interim Analysis***<br><br>***This study is the first administration of RTS,S/AS02D to infants. The interim analysis scheduled to be carried out 1 month post Dose 3 of RTS,S/AS02D or Engerix-B will provide safety and immunogenicity data prior to the final study report becoming available. The resulting safety and immunogenicity data will be used by clinical development staff at GSK Biologicals and MVI to check the assumptions on which the future clinical development of the candidate RTS,S/AS02D vaccine is based, and allow for any necessary amendments.***
**Section 3:** Recording of serious adverse events will be throughout the study period. They will be captured through the morbidity surveillance system at Ilha Jossina Health Center***, Taninga Health Center*** and Manhiça District Hospital. In addition all enrolled children will be visited at home monthly by field workers until study conclusion to ensure complete identification of all SAEs.
**Section 4.1.2:** Ilha Josina is located 50 km to the north of Manhiça, at the confluence of two rivers. ***Taninga is located 34 km to the north east of Manhiça, on the banks of the Incomati River.***

Section 4.1.5.2: Ilha Josina Health Center is the closest health center for ~~all~~ ***most*** study children. It does not have inpatient facilities. Currently (October 2004) in addition to the government staff of 1 nurse and 1 midwife it is strengthened by study personnel: 1 Medical Agent (Agente de medicina), 1 microscopist and 2 field workers. Children requiring inpatient management will be transferred to Manhiça District Hospital.

~~The~~ ***Ilha Josina*** Health Center operates during normal office hours, but 24 hour assistance is available at the Health Center. A nurse and midwife live beside Ilha Josina Health Center and are contactable 24 hours a day. If subjects present to ~~the~~ ***Ilha Josina*** Health Center 'out of hours' the nurse or midwife can contact Manhiça District Hospital by telephone or radio and request an ambulance to pick up the patient and transfer them to Manhiça District Hospital.

Section 4.1.5.3: ***Taninga Health Center***

Taninga Health Center does not have inpatient facilities. The quality of care available is equivalent to that available at Ilha Josina Health Center: Currently (June 2005) in addition to the government staff of 1 nurse and 1 midwife it is strengthened by study personnel of 1 midwifery assistant, 1 Medical Agent (Agente de medicina), 1 microscopist and 2 field workers. Children requiring inpatient management will be transferred to Manhiça District Hospital.

Taninga Health Center operates during normal office hours, but 24 hour assistance is available at the Health Center. A nurse and midwife live beside Taninga Health Center and are contactable 24 hours a day. If subjects present to Taninga Health Center 'out of hours' the nurse or midwife can contact Manhiça District Hospital by telephone or radio and request an ambulance to pick up the patient and transfer them to Manhiça District Hospital.

Section 4.1.5.5: The Expanded Program on Immunization (EPI) provides vaccination free of charge to the population. In the study area, clinics are operated every weekday at the Ilha Josina Health Center ***and at the Taninga Health Center***.

Section 5.1.6.3: In the event that the trial is suspended on the recommendation of the DSMB the sponsor (GSK Biologicals) will evaluate the information. If the sponsor concurs with the DSMB's recommendation to suspend the trial, GSK Biologicals will inform the FDA that the trial has been stopped ~~permanently~~ ***temporarily***. If the sponsor's recommendation is to continue, then a report will be submitted to the FDA detailing the rationale used in reaching this decision. The agreement of the FDA will be obtained prior to restarting the trial.

Section 5.1.11: Vaccinations will take place at the EPI clinic of Ilha Josina Health Center ***and Taninga Health Center***. All vaccinations will be given by a qualified person; a nurse or a doctor or a 'medical agent of preventative medicine'. A staff member experienced in the resuscitation of children will be available at all vaccination sessions. Facilities and equipment will be available to give emergency treatment in the case of an anaphylactic reaction following administration of vaccines. All children will be observed for an hour after the administration of vaccine to evaluate and treat any acute adverse events.

**Section 5.1.14.1:** Morbidity surveillance has been in place at Manhiça District Hospital since 1996 and in Ilha Josina ***and Taninga*** since 2001 (a detailed review of the level of health care service is given in Section 4.1.5). These ~~two~~ ***three*** health facilities are the source of primary care for this area. The surveillance system will provide a comprehensive recording of all outpatient attendances, the investigational results, diagnosis and management. Prior to the start of the study, all parent(s)/guardian(s) of study children will be educated on the appropriate action they should take if their child becomes unwell at any time during the study period. They will be asked not to medicate their child at home, but to seek medical care at ~~either~~ Ilha Josina Health Center***, Taninga Health Center*** or Manhiça District Hospital.<br><br>Study staff (a nurse, medical agent or field worker) will be available 24 hours per day at Manhiça District Hospital to receive and identify study participants when they present and to ensure complete investigation and documentation of the attendance. At Ilha Josina Health Center ***and Taninga Health Center*** staff are available 24 hours a day to arrange the transport of study participants to Manhiça District Hospital (refer to Section 4.1.5.2).
**Section 5.1.14.2:** If any child is reported to be unwell at the time of a visit, the field worker will advise the parent(s)/guardian(s) to seek care at either Ilha Josina Health Center***, Taninga Health Center*** or Manhiça District Hospital.. In the event that a child is seriously ill the field worker will inform the Principal Investigator or designate and transport arranged to Ilha Josina Health Center***, Taninga Health Center*** or Manhiça District Hospital, if judged appropriate by the responsible clinician.
**Section 5.1.15:** Ilha Josina Health Center ***and Taninga Health Center*** provide~~s~~ primary health care between 8 am to 4 pm Monday to Friday. Medical attention is available on a 24 hour basis at Manhiça District Hospital. Subjects presenting 'out of hours' to Ilha Josina Health Center ***or Taninga Health Center*** will be provided with transport to Manhiça District Hospital. A detailed description of the facilities available at Manhiça District Hospital are provided in Section 4.1.5.1. A detailed description of the facilities available at Ilha Josina Health Center is provided in Section 4.1.5.2. ***A detailed description of the facilities available at Taninga Health Center is provided in Section 4.1.5.3.***
**Section 5.1.16:** If a child presenting to Ilha Josina Health Center***, Taninga Health Center*** or Manhiça District Hospital is reported to have had fever within the preceding 24 hours or has a documented fever (defined as axillary temperature ≥ 37.5ºC) then blood will be collected for determination of malaria parasites and hemoglobin by the CISM laboratory staff. Duplicate blood slides will be taken and labeled with the same unique laboratory ID number, which will also be recorded on the CISM clinic morbidity surveillance questionnaire. Both slides will be taken to the CISM Laboratory and stained with Giemsa Stain. One will be read immediately (within 2 hours) and the result reported to guide diagnosis and management. The second slide will be kept for a later reading, according to CISM SOPs, and will determine parasite density for data analysis. Both slides will be stored at CISM. In addition, blood samples will be taken and stored for parasite genotyping.<br><br>The clinical case definition for malaria currently used at Ilha Josina Health Center, ***Taninga Health Center*** and Manhiça District Hospital is a history of fever within the previous 24 hours or documented fever (temperature ≥ 37.5°C) at the time of presentation plus any level of asexual *P. falciparum* parasitemia. This will not be changed for the trial and all cases meeting this case definition will receive treatment for malaria.

**Section 5.1.17:** For the surveillance period for malaria infection, a field worker visit will consist of visiting the child at home and completing a brief surveillance for infection morbidity questionnaire, which will include the reporting of malaria symptoms and a record of axillary temperature. If the child is well and afebrile, blood-sample will be taken and examined for malaria parasitemia and parasite genotyping. These blood slides will be Giemsa-stained and read to determine the presence of parasites at Ilha Josina Health Facility ***or Taninga Health Center***. Treatment for children not considered to be clinically ill will be sent by a field worker to all blood-slide positive children within 36 hours of the original blood slide being taken.<br><br>In the event that a child is reported as having a history of fever within the preceding 24 hours or has a documented axillary temperature of ≥37.5°C, no blood slides will be taken by the field worker but transport will be arranged by a project vehicle to Ilha Josina Health Facility ***or Taninga Health Center***. The surveillance for infection morbidity questionnaire will record that the child was transferred to Ilha Josina Health Facility ***or Taninga Health Center, as appropriate***. There the child will be reviewed by a clinician or medical agent or assistant, who will complete the CISM clinic morbidity surveillance questionnaire and the child will be managed according to the procedures detailed above (see Section 5.1.14) and a blood sample will be taken for determination of parasitemia and parasite genotyping. If parasitemia is detected in the blood slides taken on arrival at Ilha Josina Health Facility ***or Taninga Health Center***, the child will be treated as appropriate.
**Section 8.7.2:**<br><br>**Local GSK Study Monitor**<br><br>~~Andreas Pakendorf &~~ Adelheid Davis,<br>GlaxoSmithKline,<br>Private Bag X173,<br>Bryanston 2021,<br>South Africa.<br><br>Tel: +27.11.745.63.24<br>Fax: +27.11.745.74.95<br>email: ~~andreas.m.pakendorf@gsk.com &~~ adelheid.a.davis@gsk.com
**Appendix C:** The site will compile a list of children whose mothers have expressed an interest in enrolling them in the study and who have been tested for HBsAg and HIV and found to be negative. When these infants are presented at an EPI clinic in Ilha Josina ***Health Center or Taninga Health Center,*** study personnel will seek individual informed consent for each child from the parent(s)/guardian(s) in privacy. Parent(s)/guardian(s) will again be informed about the study objectives and procedures including immunization and blood collection and they will be encouraged to ask and clarify questions about the trial. The parent(s)/guardian(s) understanding of the Informed Consent form will be verified by use of an oral assessment questionnaire (outlined in CISM SOPs). The parent(s)/guardian(s) and the witness will sign the Informed Consent form. Infants whose parent(s)/guardian(s) consent for them to enter the will be screened according to the procedures outlined in Section 5.1.10.3 of the Study Protocol.
**Appendix E:**<br>The central study coordinator, Sabine Corachan and the local monitor, ~~Andreas Pakendorf,~~ ***Adelheid Davis*** should be informed 2 days before any shipment<br><br>Sabine Corachan, Rixensart, Belgium<br><br>Telephone: +32.2.656.92.33<br>Fax: +32.2.656.61.60<br>email: sabine.corachan@gskbio.com<br><br>~~Andreas Pakendorf &~~ Adelheid Davis, Bryanston, South Africa<br>Telephone: +27.11.745.63.24<br>Fax: +27.11.745.74.95<br>email: ~~andreas.m.pakendorf@gsk.com &~~ adelheid.a.davis@gsk.com

Appendix F: Briefly, whole blood taken from the subject at various time points, is stimulated by pools of peptides representing the aminoacid sequence of the CS antigen. The antigen-specific CD4+ and CD8+ T cells producing IFN-gamma ***IL-2 and TNF alpha*** are enumerated by flow cytometry following conventional imunofluorescence labelling of cellular phenotype and intracellular cytokines production. The data are expressed as frequency of CD4+ or CD8+ T cells expressing the cytokine.
