# Supplementary material for: Safety, Immunogenicity and Duration of Protection of the RTS,S/AS02D Malaria Vaccine: One Year Follow-Up of a Randomized Controlled Phase I/IIb Trial
Source: PLoS One. 2010 Nov 4;5(11):e13838. doi: 10.1371/journal.pone.0013838 (PMC2973956; doi:10.1371/journal.pone.0013838)
Supplement: Checklist S1 — CONSORT Checklist (7.60 MB RTF) [file pone.0013838.s002.rtf]

CONSORT 2010 checklist of information to include when reporting a randomised trial*

Section/Topic	Item No	Checklist item	Found in section	
Title and abstract	
	1a	Identification as a randomised trial in the title	Title 
	
	1b	Structured summary of trial design, methods, results, and conclusions (for specific guidance see CONSORT for abstracts)	ABSTRACT
	
Introduction	
Background and objectives	2a	Scientific background and explanation of rationale	INTRODUCTION  	
	2b	Specific objectives or hypotheses	INTRODUCTION
	
Methods	
Trial design	3a	Description of trial design (such as parallel, factorial) including allocation ratio	METHODS (Study Design)
	
	3b	Important changes to methods after trial commencement (such as eligibility criteria), with reasons	n/a	
Participants	4a	Eligibility criteria for participants	Study design
Eligibility criteria are summarised in the current manuscript. The complete list is described in the study protocol.	
	4b	Settings and locations where the data were collected	METHODS (Study site)
 	
Interventions	5	The interventions for each group with sufficient details to allow replication, including how and when they were actually administered	Study design 
Vaccination procedures are only briefly described in the current manuscript, but were described in detail in the article reporting the primary analysis (Aponte, 2007).	
Outcomes	6a	Completely defined pre-specified primary and secondary outcome measures, including how and when they were assessed	METHODS  
	
	6b	Any changes to trial outcomes after the trial commenced, with reasons	n/a	
Sample size	7a	How sample size was determined	METHODS (Study design)	
	7b	When applicable, explanation of any interim analyses and stopping guidelines	n/a	
Randomisation:				
 Sequence generation	8a	Method used to generate the random allocation sequence	Randomization methods are not specified in the current manuscript. They were described in the article reporting the primary  (Aponte JJ, 2007)	
	8b	Type of randomisation; details of any restriction (such as blocking and block size)	Described in the primary report (Aponte JJ, 2007).	
 Allocation concealment mechanism	9	Mechanism used to implement the random allocation sequence (such as sequentially numbered containers), describing any steps taken to conceal the sequence until interventions were assigned	Described in the primary report (Aponte JJ, 2007). 	
 Implementation	10	Who generated the random allocation sequence, who enrolled participants, and who assigned participants to interventions	Specified in the study protocol, not in the current manuscript 	
Blinding	11a	If done, who was blinded after assignment to interventions (for example, participants, care providers, those assessing outcomes) and how	Described in the primary report (Aponte JJ, 2007). 	
	11b	If relevant, description of the similarity of interventions	n/a	
Statistical methods	12a	Statistical methods used to compare groups for primary and secondary outcomes	STATISTICAL METHODS
	
	12b	Methods for additional analyses, such as subgroup analyses and adjusted analyses	STATISTICAL METHODS
	
Results	
Participant flow (a diagram is strongly recommended)	13a	For each group, the numbers of participants who were randomly assigned, received intended treatment, and were analysed for the primary outcome	RESULTS 
	
	13b	For each group, losses and exclusions after randomisation, together with reasons	RESULTS	
Recruitment	14a	Dates defining the periods of recruitment and follow-up	METHODS (study site)
	
	14b	Why the trial ended or was stopped	n/a	
Baseline data	15	A table showing baseline demographic and clinical characteristics for each group	Baseline characteristics were reported in the article presenting the primary analysis (Aponte JJ, 2007)	
Numbers analysed	16	For each group, number of participants (denominator) included in each analysis and whether the analysis was by original assigned groups	RESULTS (Tables 1, 2, 3 and 4) 	
Outcomes and estimation	17a	For each primary and secondary outcome, results for each group, and the estimated effect size and its precision (such as 95% confidence interval)	RESULTS (Tables 3 and 4)	
	17b	For binary outcomes, presentation of both absolute and relative effect sizes is recommended		
Ancillary analyses	18	Results of any other analyses performed, including subgroup analyses and adjusted analyses, distinguishing pre-specified from exploratory		
Harms	19	All important harms or unintended effects in each group (for specific guidance see CONSORT for harms)	RESULTS 	
Discussion	
Limitations	20	Trial limitations, addressing sources of potential bias, imprecision, and, if relevant, multiplicity of analyses	DISCUSSION
	
Generalisability	21	Generalisability (external validity, applicability) of the trial findings	DISCUSSION
	
Interpretation	22	Interpretation consistent with results, balancing benefits and harms, and considering other relevant evidence	DISCUSSION
	
Other information		
Registration	23	Registration number and name of trial registry	ABSTRACT
	
Protocol	24	Where the full trial protocol can be accessed, if available	Supplementary files	
Funding	25	Sources of funding and other support (such as supply of drugs), role of funders	FUNDING STATMENT
	

*We strongly recommend reading this statement in conjunction with the CONSORT 2010 Explanation and Elaboration for important clarifications on all the items. If relevant, we also recommend reading CONSORT extensions for cluster randomised trials, non-inferiority and equivalence trials, non-pharmacological treatments, herbal interventions, and pragmatic trials. Additional extensions are forthcoming: for those and for up to date references relevant to this checklist, see www.consort-statement.org.
